# Efficient CO_2_ electroreduction on facet-selective copper films with high conversion rate

**DOI:** 10.1038/s41467-021-26053-w

**Published:** 2021-09-30

**Authors:** Gong Zhang, Zhi-Jian Zhao, Dongfang Cheng, Huimin Li, Jia Yu, Qingzhen Wang, Hui Gao, Jinyu Guo, Huaiyuan Wang, Geoffrey A. Ozin, Tuo Wang, Jinlong Gong

**Affiliations:** 1 School of Chemical Engineering and Technology, Tianjin University, Tianjin, 300072 China; 2 Key Laboratory for Green Chemical Technology of Ministry of Education, Tianjin University, Tianjin, 300072 China; 3 Collaborative Innovation Center of Chemical Science and Engineering (Tianjin), Tianjin, 300072 China; 4 Department of Chemical Engineering, Stanford University, Stanford, CA USA; 5 Department of Chemistry, University of Toronto, 80 St. George Street, Toronto, ON Canada; 6 Joint School of National University of Singapore and Tianjin University, International Campus of Tianjin University, Binhai New City, Fuzhou 350207 China

**Keywords:** Electrocatalysis, Solar fuels, Electrochemistry

## Abstract

Tuning the facet exposure of Cu could promote the multi-carbon (C2+) products formation in electrocatalytic CO_2_ reduction. Here we report the design and realization of a dynamic deposition-etch-bombardment method for Cu(100) facets control without using capping agents and polymer binders. The synthesized Cu(100)-rich films lead to a high Faradaic efficiency of 86.5% and a full-cell electricity conversion efficiency of 36.5% towards C2+ products in a flow cell. By further scaling up the electrode into a 25 cm^2^ membrane electrode assembly system, the overall current can ramp up to 12 A while achieving a single-pass yield of 13.2% for C2+ products. An insight into the influence of Cu facets exposure on intermediates is provided by in situ spectroscopic methods supported by theoretical calculations. The collected information will enable the precise design of CO_2_ reduction reactions to obtain desired products, a step towards future industrial CO_2_ refineries.

## Abstract (web summary)

Regulation of Cu facets to promote electrocatalytic CO_2_ reduction is interesting and challenging. Here the authors describe a deposition-etch-bombardment synthetic approach to prepare Cu(100)-rich thin film electrodes for CO_2_ electroreduction with over 50% ethylene Faradaic efficiency at a total current of 12 A.

## Introduction

The renewable electricity-powered CO_2_ reduction has been considered as one promising route to the product of chemical feedstocks, which might close the carbon loop^1,2^. Multi-carbon (C2+) products such as ethylene, ethanol, n-propanol, etc. are important raw materials in the chemical industry or can be used directly as fuels, thus the efficient CO_2_ reduction to C2+ products system is essential to the production of high-value commodity chemicals with a net negative carbon emissions footprint. Among various CO_2_ reduction catalyst materials, Cu has been studied extensively as it can produce C2+ products with appreciable selectivity^3^. Among numerous ways to promote C2+ formation^4–8^, adjusting the facet of Cu-based catalysts is an effective method since the dimerization reaction is facet sensitive^9^. According to the theoretical calculations, the Cu(100) facet can significantly lower the dimerization energy barrier^10^. Thus, it is an effective approach to improve the selectivity toward C2+ by designing Cu catalysts with Cu(100) as the dominant exposed facet^10^. Colloidal chemistry is an inexpensive, simple, and widely used method for facet exposure control. It produces catalysts with preferential exposure of specific facets by using capping agents to manipulate surface energies, which changes the growth rates of different facets to alter the shape of nanocatalysts^11,12^. Recently, colloidally synthesized Cu nanocubes rich in Cu(100) facets were shown to achieve a Faradaic efficiency (F.E.) of ~57% towards ethylene^13^. However, the corresponding electricity conversion efficiency (E.C.E.) and single-pass yield have been rarely reported. Some studies also propose that the intermediates along the CO_2_ reduction pathways can control the formation of specific facets, where the adsorption of the intermediates plays a role analogous to that of capping agents^14,15^. Nonetheless, these approaches require specific chemicals, such as capping agents, to be selectively adsorbed on particular facets to reduce the surface energy^11,12^. However, the effectiveness of the colloidal method might compromise to some extent since low-index facets of fcc transition metals often possess similar surface energies (1.25 J cm^−2^ for Cu(111) and 1.43 J cm^−2^ for Cu(100))^15,16^. Besides, residual capping agents left on the catalyst surface could block catalytically active sites^17,18^. Moreover, the colloidally synthesized catalyst needs to be dispersed in a solution containing polymer binders (such as Nafion) to form a well-mixed ink before being drop-casted on the conductive support to form an electrode, which is not always compatible with the catalytic electrode system due to agglomeration and the peeling-off of catalysts especially when scaling up the electrodes^19,20^. At the same time, due to the existence of the cross-linked network formed by polymer binders^21^, the contact between the colloidally synthesized catalyst and the conductive substrate is weak^22,23^, and plenty of active sites are further encapsulated^20^, which will lead to slow electron transfer and low E.C.E. Thus, it is highly desirable to develop a novel approach to replace colloidal synthesis to prepare Cu electrodes with dominant (100) facets in one-step without using capping agents and polymer binders to achieve a high E.C.E. and a single-pass yield.

In this work, we describe the design and realization of a dynamic deposition-etch-bombardment process to produce Cu(100)-rich films as the CO_2_ reduction electrode in one-step, which could break the limitation of using capping agents while avoiding the issue of catalyst loading faced by conventional methods. This Cu(100)-rich film yields a full-cell electricity conversion efficiency of 40.5% towards C2+ products in the 4 cm^2^-membrane electrode assemble (MEA) system. This facile one-step method also makes it easier for scaling up electrodes, with a 25 cm^2^ electrode exhibiting a 13.2% single-pass yield of C2+ product at a total current of 12 A in the MEA system. Thanks to the controllable structure, the influence of facets on the adsorption and activation of intermediates is further revealed by in situ spectroscopy and density functional theory (DFT) calculation.

## Results

To amplify, when Cu atoms with high kinetic energy bombard the deposited Cu film, the high local temperature causes dynamic recrystallization^24,25^. Facets with the relatively loose atomic arrangement, like Cu(100) (Supplementary Fig. 1c), would receive less damage and remain at lower temperatures thereby acting as recrystallization centers^26,27^. As a result, the Cu(100) will preferentially grow, replacing the more densely packed Cu(111) facet (Supplementary Fig. 1b). Therefore, by controlling the kinetic energy of bombarding copper atoms, the exposure ratio of Cu(100) facets could be adjusted.

**Fig. 1:**
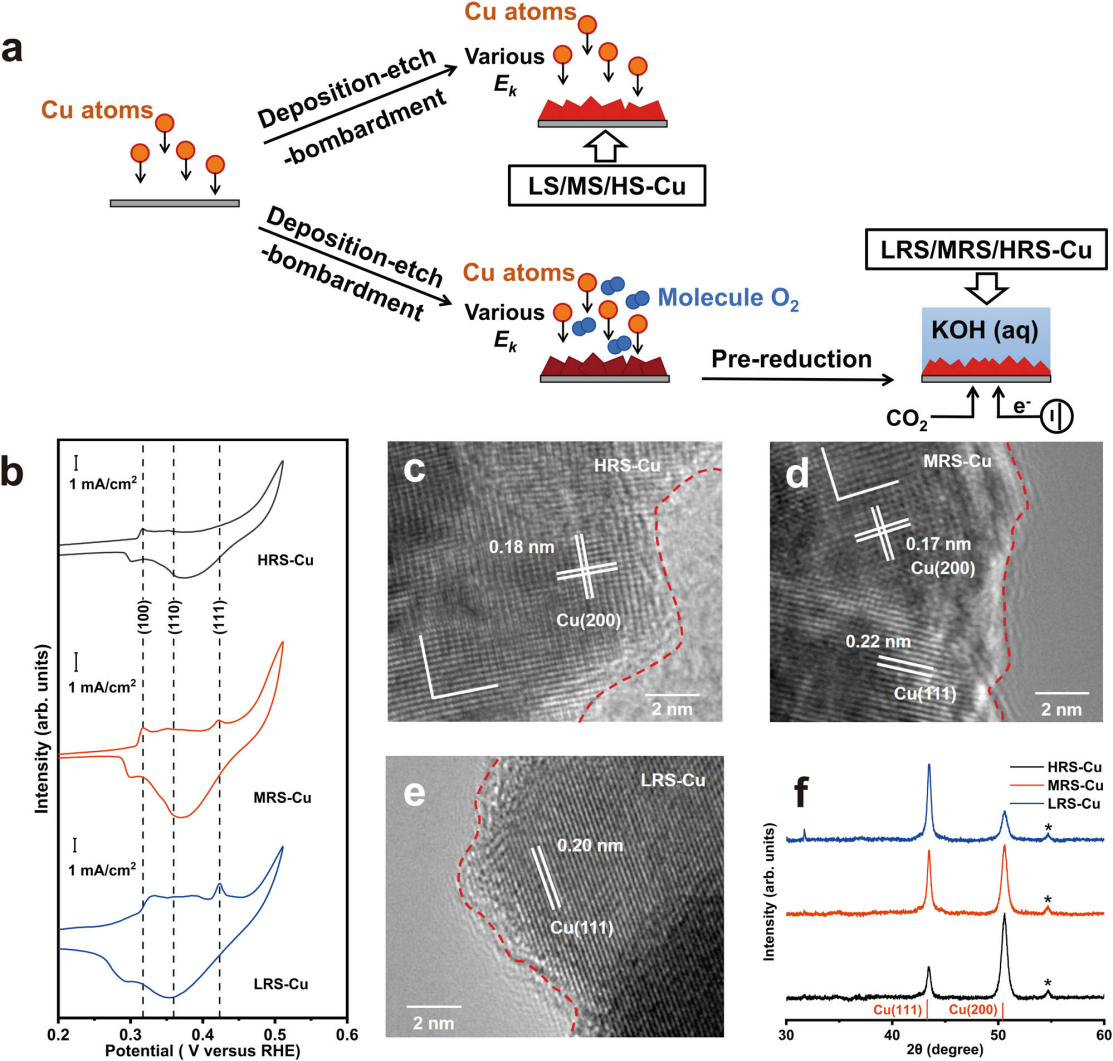
characterization of various Cu films. **a** A schematic illustration of the synthesis process for LS/MS/HS-Cu and LRS/MRS/HRS-Cu. **b** Voltammograms of resulting electrodes collected immediately after pre-reduction. Typical TEM images of **c** HRS-Cu, **d** MRS-Cu, and **e** LRS-Cu. **f** Typical XRD patterns of resulting electrodes. As visual aids, the red dash line indicates the surfaces of the resulting electrodes. The precatalysts of HRS/MRS/LRS-Cu were pre-reduced under −0.5 V versus the RHE for 1 h, and the other conditions of prereduction are the same as that of the CO_2_ reduction. The labeling of Cu(200) is used for easier comparison with XRD, in which only (200), the second-order diffraction of (100) could be detected. The peaks marked with an asterisk in XRD patterns originate from carbon-based GDL substrate.

To realize the simultaneous deposition, etching, and bombardment, high-energy radio frequency (RF) sputtering was adopted to prepare Cu(100)-rich films (details in the “Methods”), which enables the direct deposition of catalysts on carbon-based gas diffusion layers (GDLs) as electrodes in one-step (Supplementary Fig. 1a). Three typical types of sputtered Cu films with different Cu(100) proportions were obtained by adjusting the RF power to control the kinetic energy (E_k_) of the bombarding Cu atoms (Fig. 1a, details in “Methods”), resulting in low-power, medium-power, and high-power sputtered Cu films (denoted as LS-Cu, MS-Cu, HS-Cu, respectively). The obtained HS-Cu film is prone to expose the Cu(100) facet, as evidenced by transmission electron microscopy (TEM, Supplementary Fig. 2a) and X-ray diffraction (XRD, Supplementary Fig. 3a), while LS-Cu tends to form Cu(111) facet (Supplementary Figs. 2c and 3a). The energy of Cu atoms generated by thermal evaporation^2^ or low-rate sputtering process^4,28^ is usually not high enough, rendering Cu films that favor the Cu(111) facet exposure similar to LS-Cu. As different Cu facets feature distinctive OH^−^ electrochemical adsorption behaviors^29,30^, surface structures of these samples were probed by using the OH^−^ electroadsorption technique (the features were labeled by comparing the CVs to those of single crystals shown in Supplementary Fig. 4)^31^. The cyclic voltammograms (CVs) of OH_ads_ peaks (Supplementary Fig. 3b) reveal that the presence of Cu(111) is suppressed in the HS-Cu film. Therefore, this deposition-etch-bombardment process successfully realizes the replacement of Cu(100) to Cu(111) facets, adjusting the facet exposure of Cu.

However, these obtained Cu films (i.e. LS-Cu, MS-Cu, HS-Cu) lack a large electrochemically active surface area (ECSA, Supplementary Fig. 26 and Supplementary Table 1) to realize high-rate CO_2_ electrolysis^32^. To increase the ECSA, an oxidation−reduction procedure was included, during which the preferential exposure of Cu(100) could be maintained. Specifically, molecular O_2_ (with a constant partial pressure of 0.67 Pa) was introduced during this deposition-etch-bombardment process to obtain Cu_2_O as precatalysts, which could be further reduced during the pre-reduction process (−0.5 V vs. the RHE) under the same CO_2_ reduction conditions to metallic Cu with the Cu(100) facets retained (Fig. 1a, details in “Methods”). The same crystallographic relationship exists between the oxidation and reduction processes for Cu(100)/(111) and Cu_2_O(100)/(111)^10,33^, since oxygen atoms could pack into the interlayer sites of different planes of Cu, causing a lattice expansion while retaining the underlying fcc crystal structure with the inclusion of oxygen^33^. Therefore, the introduction of an oxidation-reduction step will be able to increase the ECSA while retaining the same Cu(100) exposure. Correspondingly, samples obtained by reactive sputtering followed by reduction are referred to as low-power, medium-power, and high-power reactively sputtered Cu films followed by reduction (denoted as LRS-Cu, MRS-Cu, and HRS-Cu). Typical XRD patterns (Supplementary Fig. 5a) show that all the obtained precatalysts are mainly Cu_2_O, which is confirmed by X-ray photoelectron spectroscopy (XPS), Auger spectroscopy (Supplementary Fig. 5b, c), and Raman spectroscopy (Supplementary Fig. 4d). The same type of Cu facets distribution could be maintained for these electrodes regardless of the oxidation-reduction process according to TEM (Fig. 1c–e, more images of different samples are also provided in Supplementary Figs. 7–9) and XRD (Fig. 1f), consistent with previous reports^10,34^. The different proportions of Cu(100) facet on these samples (i.e. HRS-Cu, MRS-Cu, and LRS-Cu) are also evidenced by the electrochemical OH^−^ adsorption peaks on Cu(100) and Cu(111) at potentials of ~0.33 and 0.43 V versus the RHE (Fig. 1b). Meanwhile, large ECSA is obtained for these samples (Supplementary Table 1). Thus, this one-step surfactant-free route indeed leads to nanostructured Cu films with preferred Cu(100) exposure and high surface area that could be further used as CO_2_ reduction electrodes.

To further explore the importance of the high-energy atom bombardment proposed above, another control electrode was prepared. Cu_2_O precatalyst (Supplementary Fig. 10) was obtained through wet chemistry without using capping agents (denoted as W-Cu). Then this control Cu_2_O precatalyst was airbrushed onto GDEs and pre-reduced to Cu under CO_2_ reduction conditions to form a control electrode (details in “Methods”). The typical TEM image shows that the lattice fringes corresponding to Cu(111) are widely distributed in this control electrode (Supplementary Fig. 11a)^32^. These observations are in good agreement with the XRD pattern (Supplementary Fig. 11b) and CV of OH_ads_ peaks (Supplementary Fig. 11c). It is also worth noting that LRS-Cu, MRS-Cu, HRS-Cu, and W-Cu all consist of metallic Cu only without residual oxides after the pre-reduction, as confirmed by in-situ Raman spectroscopy (Supplementary Fig. 12, details in “Methods”). Thus, this control experiment reveals that the lack of high-energy atom bombardment during the synthesis process would result in the dominant exposure of Cu(111) facets, similar to the scenario of lacking capping agents that are widely used in the synthesis of Cu nanocubes^34,35^.

**Fig. 2:**
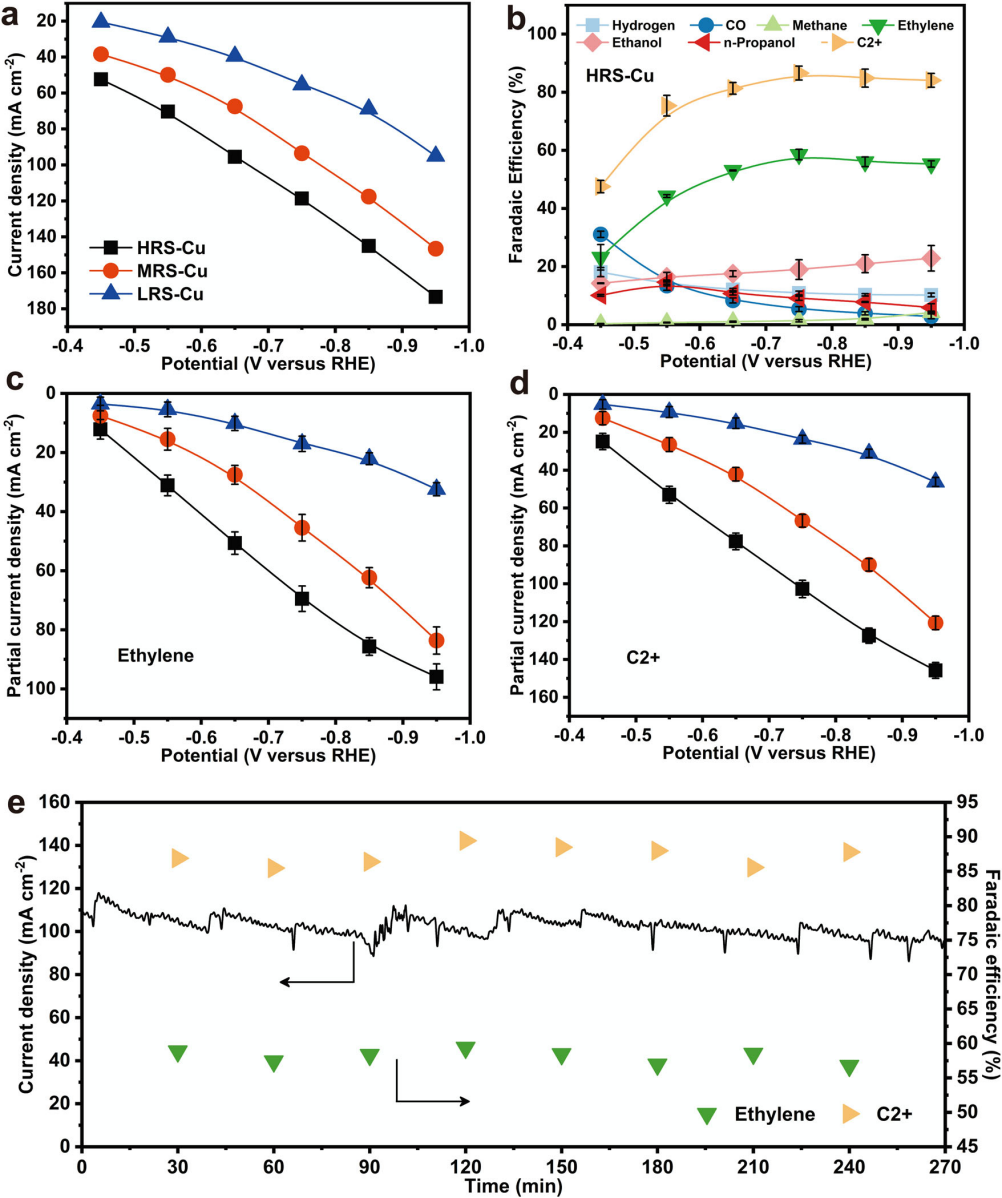
Catalytic performance of various Cu films. **a** Current−voltage (I–V) curves on various electrodes measured in CO_2_-flowed 2 M KOH electrolytes. **b** Faradaic efficiencies of CO_2_ reduction products on the HRS-Cu sample as a function of different applied potentials. **c** Ethylene and **d** C2+ products partial current densities obtained on various samples at different applied potentials. **e** Stability test over a span of 4.5 h (270 min) of CO_2_− electrolysis in 2 M KOH (aq.) at −0.75 V versus the RHE. The oscillation of the current density is due to the repeated release of O_2_ bubbles in the anode side of the flow cell. C2+ products include ethylene, ethanol, and n-propanol. Error bars represent the standard deviation from at least three independent measurements.

Before performance testing, it was confirmed that different samples (LRS-Cu, MRS-Cu, HRS-Cu, and W-Cu) possess similar mass loadings (Supplementary Table 2), catalyst layer thickness (Supplementary Fig. 13) and morphology (Supplementary Fig. 14) of Cu, which ensures a fair comparison to explore the activity difference among various samples^36–39^. The activities were evaluated at different potentials using 2 M KOH (aq.) as the electrolyte (Fig. 2a and Supplementary Figs. 16–19, details in “Methods”) in a flow cell electrolyzer (Supplementary Fig. 15) for CO_2_ reduction with an effective electrode geometric area of ~0.64 cm^2^ for both cathode and anode. As for HRS-Cu, the products detected in significant quantities were ethylene, ethanol, n-propanol, and CO. At more negative potentials, a small amount of methane was produced, and the remaining charge was attributed to the competing hydrogen evolution reaction (Fig. 2b). Compared with the control samples, the HRS-Cu exhibits a maximum F.E. of 58.6% for ethylene, 86.6% for C2+ products (containing ethylene). Consistently, the HRS-Cu sample also exhibits the largest ethylene and C2+ products partial current densities (Fig. 2c, d and Supplementary Fig. 17c, d) among the four samples at all applied potentials. Moreover, at the applied potential of −0.85 V versus the RHE, the C2+-to-C1 ratio of the HRS-Cu electrode reaches about 15.2, which largely outperforms that of its counterparts (Supplementary Fig. 18). Due to the close contact between the catalytic Cu film and the GDL substrate, the reaction system could exhibit higher E.C.E. The corresponding full-cell E.C.E. towards ethylene and C2+ products of this HRS-Cu-based reaction system reaches 24.8 and 36.5%, respectively, exceeding the efficiency of many other reaction systems reported so far (see below). The stability of the HRS-Cu was also examined under a constant applied potential of −0.75 V versus the RHE for 4.5 h (270 min), where ethylene and C2+ F.E.s remained stable over the test duration (Fig. 2e and Supplementary Fig. 19). Although the surface of the Cu-based catalysts may undergo reconstruction during the reaction to obtain Cu(100)^40^, the HRS-Cu, MRS-Cu, and LRS-Cu samples after use show similar surface exposures as before the reaction (Supplementary Figs. 20–22), indicating that the reconstruction process could not significantly change the main facet exposure, and thus surface reconstruction alone cannot guarantee electrodes similar to the HRS-Cu (showing a predominant Cu(100) exposure without undergoing reconstruction). The mass of the HRS-Cu sample after the reaction is also almost the same as that before the reaction (Supplementary Table 3), which further indicates that the firm conjunction between the substrate and the active composition. If the hydrophobicity of GDLs can be further improved^2^ and the reaction rate of carbonate formation can be reduced^41^, the stability of the sample in the flow cell will be longer.

To examine the influence of Cu(100) exposure over C2+ products formation in CO_2_ reduction, Tafel analyses (Supplementary Fig. 23) were conducted on various samples. It is clear that C2+ products formation depends strongly on the surface structure. With the increasing exposure of Cu(100) facets, a lower change of the Tafel slope can be observed, which indicates that the Cu(100) surface is beneficial to the formation of C2+ products. However, the Tafel slope of all samples locates at approximately 120−140 mV dec^−1^, implying that they might share the same rate-determining step (details in “Methods”), which is further validated by the results of CO partial pressure dependence experiments (Supplementary Figs. 24, 25). To further compare the reaction rates, the performances are normalized to the ECSA (details in “Methods”). Obviously, the current densities of specific ethylene and C2+ products of HRS-Cu are larger than other electrodes (Supplementary Fig. 27), which indicates the highest intrinsic activities of HRS-Cu.

**Fig. 3:**
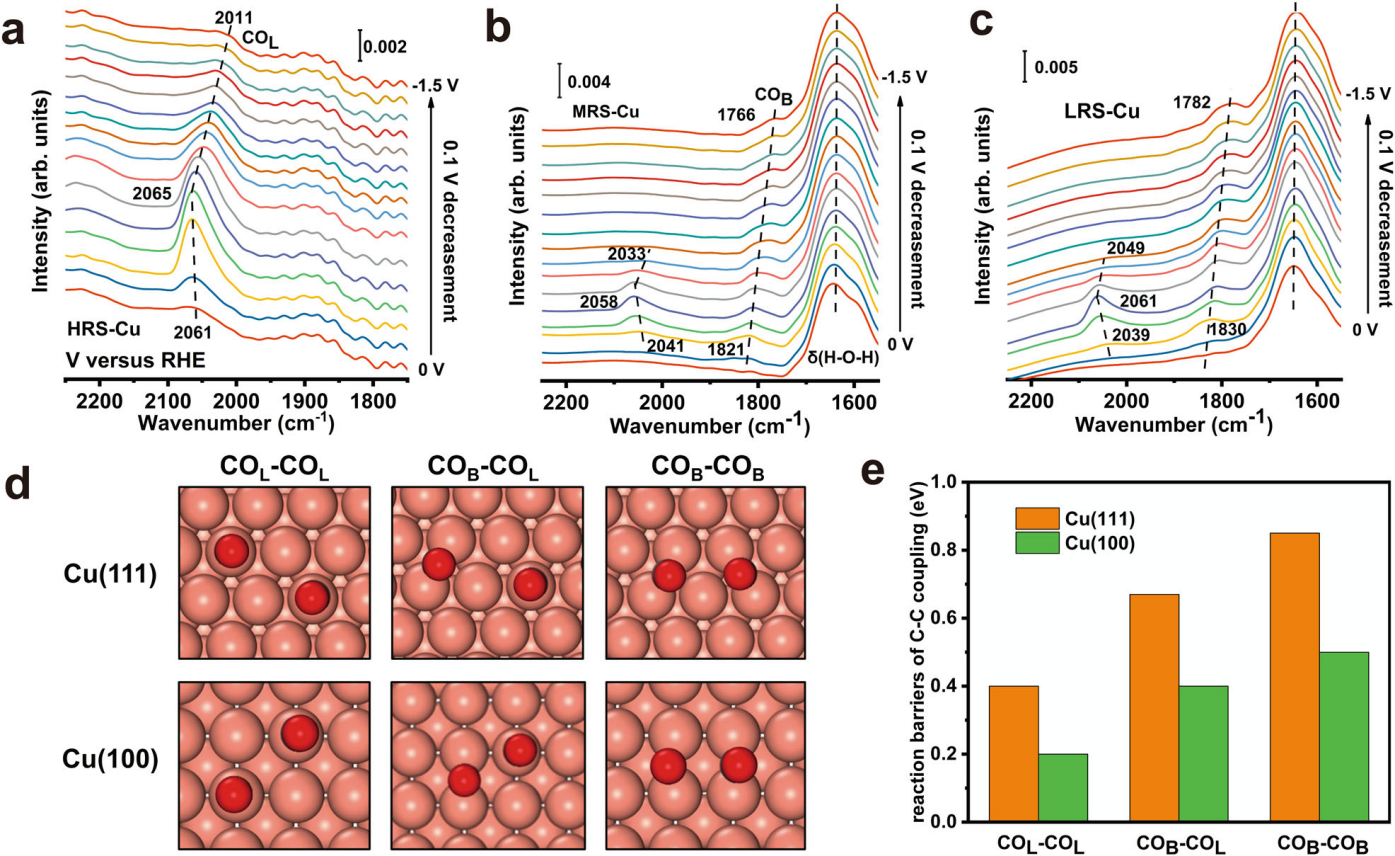
Spectroscopic investigations of various Cu films and DFT calculations on different Cu facets. In situ ATR-SEIRAS spectra of **a** HRS-Cu; **b** MRS-Cu; **c** LRS-Cu; **d** adsorption geometry for different C−C coupling precursors (i.e., CO_L_−CO_L_, CO_B_−CO_L_, CO_B_−CO_B_), where solvent molecules are not presented to show the adsorbate configurations; **e** reaction barriers for C−C coupling from different precursors on Cu(100) and Cu(111) facets.

Understanding the behaviors of adsorbates is essential to the inquiry into the nature of the catalytic activity. Thus, in-situ attenuated total reflectance (ATR)-surface-enhanced infrared absorption spectroscopy (ATR-SEIRAS, details in “Methods”) was applied to investigate the effect of facets exposure on the adsorption of intermediates. When the potential is swept from −0.1 to −1.5 V versus the RHE, a positive band centered at ~2050 cm^−1^ which corresponds to the linear-bond CO (CO_L_, a reactive adsorbed species) is observed on all electrodes (Fig. 3a–c and Supplementary Fig. 28)^42–44^. Meanwhile, a small positive band centered at ~1800 cm^−1^, which corresponds to the bridge-bond CO (CO_B_, an unreactive adsorbed species)^44^ is also observed on the surface of other samples except the HRS-Cu. These results of the ATR-SEIRAS are also corroborated by the corresponding Raman spectra, where the HRS-Cu surface was covered by abundant CO_L_^45,46^, while the LRS-Cu surface exhibited a pronounced appearance of CO_B_ (Supplementary Fig. 30). The appearance of CO_B_ might result from the interconversion of CO_L_. According to the literature, when the *CO (*denotes the adsorbed species) coverage of the electrode decreases, a fraction of CO_L_ would convert to CO_B_^44^. Therefore, the increase of CO_B_ ratio reveals the reduction of surface *CO coverage. By calculating the CO_B_ to CO_L_ ratio of different samples, the *CO coverage might decrease as the exposure of Cu(100) facets decreases (Supplementary Fig. 29). Normally, *H and *CO occupy most of the surface sites, so they are in direct competition with each other for surface sites. Meanwhile, the surface coverage of *CO eventually influences the distribution of products derived from CO_2_ reduction. It has been previously speculated that when the surface cannot maintain a high *CO coverage, the corresponding *H coverage will increase, inhibiting the dimerization of *CO to produce C2+ products, thereby shifting the selectivity to C1 products and H_2_^47–49^, which is also demonstrated by the results of our CO partial pressure dependence experiments (Supplementary Figs. 24, 25). Meanwhile, the results of CO_2_ reduction performance support the above speculation. The LRS-Cu and the W-Cu with a lower exposure of Cu(100) facets exhibit the highest F.E. of methane, meanwhile, the methane F.E. increases rapidly as the potential becomes negative (Supplementary Fig. 31). Thus, these ATR-SEIRAS results prove that Cu(100) facet might be a type of strong *CO adsorption site, and a higher exposure of Cu(100) facets is beneficial to the increase of strong adsorption sites on the surface that maintains a higher *CO coverage on the surface, thereby leading to the catalyst with a higher C−C coupling performance, which is also supported by other experimental and computational studies^50–52^.

Since the ATR-SEIRAS cannot directly provide the adsorption energy and activation barrier of the intermediate, DFT calculations on Cu(111) and Cu(100) (3×3) models were performed to further understand the facet exposure effect (Supplementary Fig. 32, details in “Methods”). Based on the Tafel analysis and CO partial pressure dependence study (Supplementary Figs. 23, 24), the energetics of *OCCO formation is chosen as the main consideration. Previous work has found that the solvent and cation effects can stabilize *COCO, hence an appropriate electrochemical interface was built up to explore the mechanism of this dimerization procedure (Fig. 3d and Supplementary Figs. 34–36)^53^.

From the DFT calculation results, the binding of *CO is the weakest on the Cu(111) facet (Supplementary Fig. 33), which is not suitable to build up a sufficient coverage of *CO on the surface to promote the kinetics of C−C coupling. Therefore, LRS-Cu and W-Cu would shorten the *CO stay and reduce the *CO coverage as compared to HRS-Cu, leading to a higher CO F.E. (Supplementary Fig. 16). In addition, the barriers of various C−C coupling processes are always higher on the Cu(111) facet than on Cu(100) facet, implying that *CO dimerization is the most sluggish step on the Cu(111) facet (Fig. 3e). Thus, LRS-Cu and W-Cu are less active in catalyzing the reduction of CO_2_ to C2+ products. These theoretical calculations are in good agreement with our ATR-SEIRAS and performance test results.

**Fig. 4:**
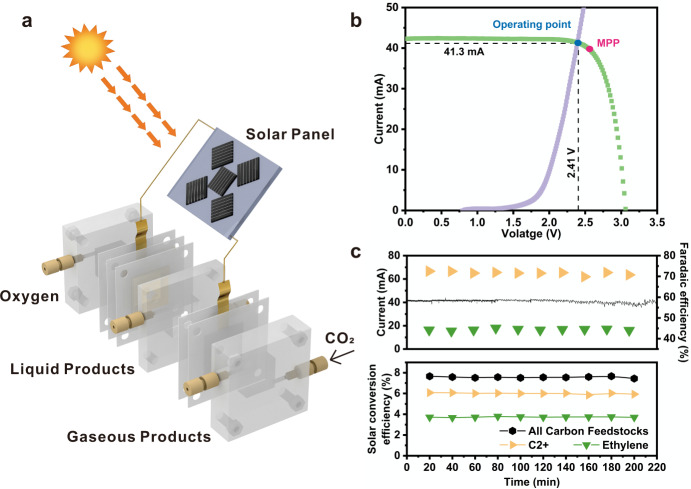
Solar-powered CO_2_ reduction. **a** Schematic of the PV-EC system. **b** Photovoltaic and electrocatalytic I-V behaviors. The photovoltaic performance is shown under light (green) with the MPP marked by a red dot. The measured operating current of the CO_2_ electrolysis system (cathode, anode, and anion exchange membrane) at different voltages has been marked by the purple curve. The observed long-term operating point is marked by a blue dot, with the black dashed lines showing the corresponding current and voltage. **c** Faradaic efficiency towards ethylene and C2+ products, solar current, and solar conversion efficiency as a function of reaction time. Carbon feedstocks include CO, methane, ethylene, ethanol, and n-propanol. C2+ products include ethylene, ethanol, and n-propanol.

Due to the impressive E.C.E of this HRS-Cu-based reaction system, a PV-EC system (Fig. 4a) was further constructed to demonstrate the photosynthesis of C2+ products. Using the same test conditions (electrolyzer, cathode, anode, and electrolyte, details in “Methods”) for measuring the CO_2_ reduction performance on the cathode side (as in Fig. 2), it is found that an overall cell voltage of about 2.5 V is required (Fig. 4b) to obtain an operating current density varying from 60 to 70 mA cm^−2^ (Fig. 2a), which could be translated to a cathodic potential of ~−0.55 V versus the RHE that yields ~45 and ~72% F.E. for ethylene and C2+ products (containing ethylene), respectively (Fig. 2b). The deviation of these F.E.s from the maximized F.E.s for ethylene and C2+ products is because of the matching between electrolyzer and the solar panel towards the maximum power point (MPP) of the solar panel. Considering that the effective area of the HRS-Cu sample is ~0.64 cm^2^, the operating current varies from 38.4 to 44.8 mA. The widely available p−n^+^ solar cells, with an open circuit potential of ~0.6 V and a short-circuit current density of ~36 mA cm^−2^, were selected and cut into ~1.14 cm^2^ pieces. By connecting five of them in series, the solar panel would provide the suitable voltage and current for the CO_2_ reduction electrolyzer near the MPP of the solar panel (i.e., the PV system). I−V curve of the obtained solar panel was measured under simulated AM 1.5G 1-sun illumination (Fig. 4b). This curve crosses the I−V curve of the electrolyzer at the point (i.e., the operating point) where its cathodic current and voltage are ~41.3 mA and ~2.41 V, respectively, which matches well with the MPP output metrics of the solar panel (39.8 mA at 2.53 V), indicating the optimum solar-to-electricity conversion process in our integrated device.

During ~3.7 h (220 min) of electrolysis powered by 1-sun solar illumination, the cathodic current of the electrolyzer was stable at ~41.3 mA and the F.E. of ethylene and C2+ products stabilized at ~45 and ~72%, respectively (Fig. 4c and Supplementary Figs. 37–39). Although this selectivity is already deviated from the maximum F.E.s owing to MPP matching, such a PV-EC system still yields a solar-to-ethylene efficiency of ~4.0% and a solar-to-C2+ products efficiency of ~6.0% (Fig. 4c) under simulated 1 Sun illumination, which exceeds the efficiency of general natural photosynthesis for producing carbohydrates (3−6%)^54^. Such efficiency is also able to match a recent state-of-the-art perovskite solar cells-powered Cu−Ag bimetallic reaction system^55^, while a longer stability and larger CO_2_ reduction current are obtained in this system. The performance of this work also exceeds that of most previously reported copper-based PV-EC systems (Supplementary Table 4), which provides a benchmark for solar conversion efficiency while using feasibly available Si-based solar cells and earth-abundant low-cost electrode materials.

**Fig. 5:**
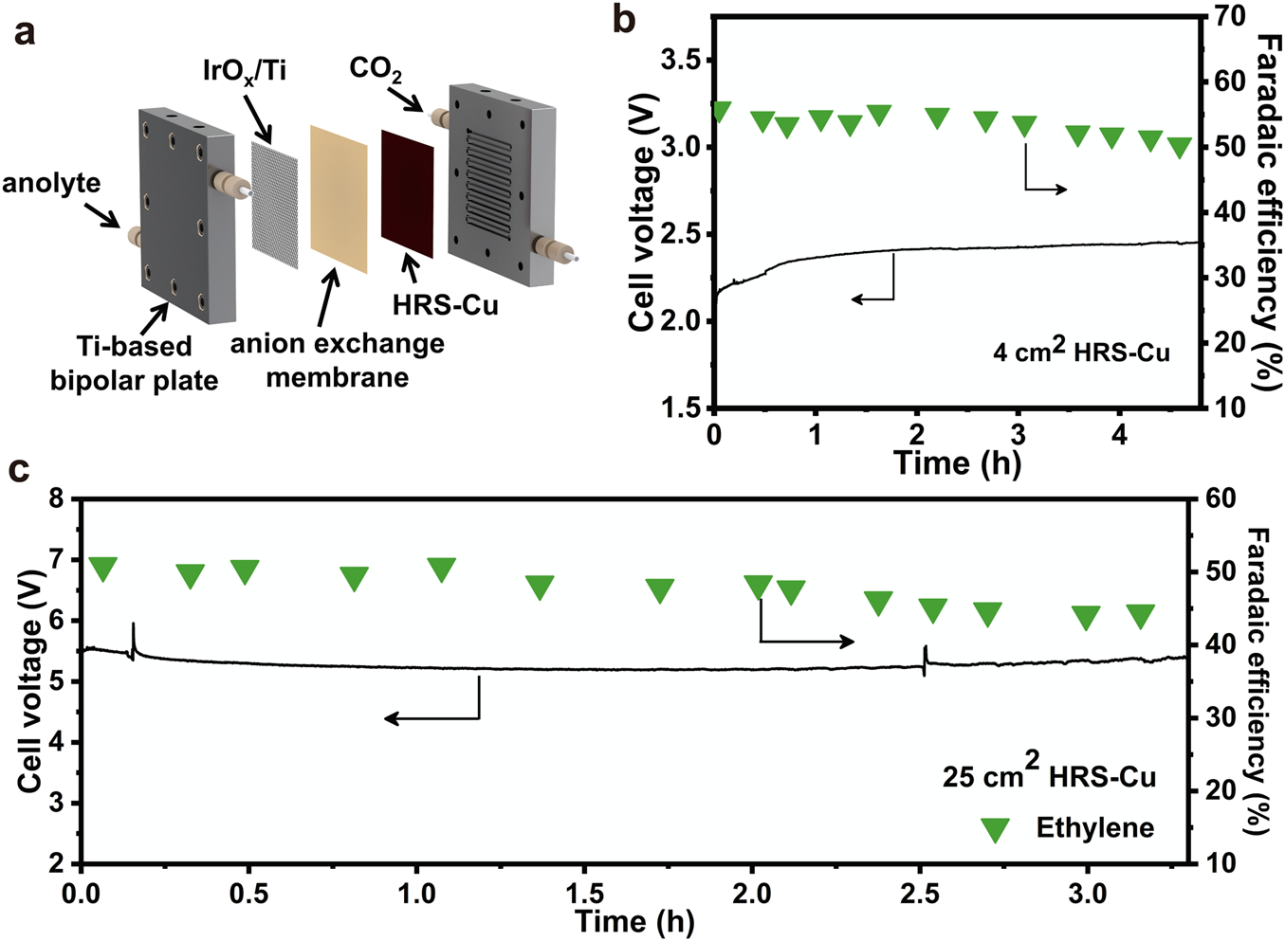
Scaling up the CO_2_ reduction system. **a** Schematic of the MEA system. The gaskets are not shown. **b** Stability test over a span of ~4.5 h of CO_2_-electrolysis in a 4 cm^2^-MEA system at the total current density of 120 mA cm^−2^. **c** Stability test over a span of ~3.5 h of CO_2_-electrolysis in a 25 cm^2^-MEA system at the total current of 12 A. The decrease in ethylene F.E. and the increase in cell voltage may be attributed to the formation of carbonate on the cathode side and the cathode water flooding.

The membrane electrode assemble system (MEA) was further adopted to scale up the CO_2_ reduction system (Supplementary Fig. 40, details in “Methods”). On the cathode side, humidified CO_2_ gas was supplied (Fig. 5a), which reduces the direct contact between the catalyst and the aqueous electrolyte, while reducing the ohmic resistance of the electrolyte^56,57^. At the same time, the one-step deposition-etch-bombardment process proposed in this work is based on a widely used vacuum deposition process similar to the photovoltaic industries, thus its marginal cost for producing large-area electrodes can be largely reduced with mass production. In addition, this method does not require additional catalyst loading steps and does not use additional chemicals, accelerating the continuous preparation of large-area electrodes and avoiding potential electrode contamination. Therefore, the combination of a MEA reaction system and this preparation method is particularly suitable for the scale-up of CO_2_ reduction.

The electrode area was enlarged to 4 cm^2^, and the CO_2_ reduction performance test was performed in the MEA system (the area of cathodic flow fields is 4 cm^2^, referred as to the 4 cm^2^-MEA) at a current density of 120 mA cm^−2^ corresponding to the maximum ethylene F.E. (58.6%) in the flow cell system (Fig. 5b). The optimal ethylene F.E. reaches 55.8%, while the corresponding full-cell E.C.E of ethylene and C2+ products increase to 26.4 and 40.2%, respectively. After ~4.5 h of operation (Supplementary Fig. 41), the ethylene selectivity (50.4%) still remains above 90% of the initial value (55.8%). Although many works have obtained impressive CO_2_ reduction selectivity, the single-pass yield for C2+ products is generally low (mostly below 3%, Supplementary Table 5). In order to improve the single-pass yield of C2+ products, it is vital to further increase the current density and electrode area without affecting the transport of CO_2_ (details in the “Methods”). Using the one-step method proposed in this work, a HRS-Cu electrode with 25 cm^2^ activity area could be easily fabricated with the deposition-etch-bombardment process (Supplementary Fig. 42). In a 25 cm^2^ MEA system (i.e., the area of cathodic flow fields is 25 cm^2^), the optimal ethylene F.E. of HRS-Cu reached 50.9% and maintained above 45% (Fig. 5c and Supplementary Fig. 43) after ~3.5 h of operation at a total current of 12 A (i.e., 480 mA cm^−2^). The corresponding single-pass yield of CO_2_ towards C2+ products increased to and 13.2%, with ethylene increased to 12.0%. However, a corresponding decrease in E.C.E. was observed (Supplementary Table 5). Therefore, using more efficient anode materials, while controlling the operating conditions of the device such as temperature and pressure, or designing a new flow field structure to enhance CO_2_ mass transfer may be effective ways to further improve the E.C.E.

## Discussion

In conclusion, this work demonstrates a deposition-etch-bombardment process that feasibly increases the exposure of Cu(100) facets in low-cost nanostructured Cu films. This strategy obviates the use of capping agents, achieving precise control of surface structures in a one-step approach. The obtained Cu(100)-rich film functions as a high-performance electrode for CO_2_ reduction towards ethylene and C2+ products. In flow cell, it realized ethylene and C2+ (containing ethylene) F.E.s of 58.6 and 86.6%, respectively. In addition, this deposition-etch-bombardment method bypasses the electrode assembly process, avoids the use of polymer binders, enhances the contact between the catalytic film and the substrate. Due to the above advantages, in flow cell, a corresponding full-cell E.C.E. of 24.8 and 36.5% for ethylene and C2+ products were obtained, respectively, which is a notable advance over existing single metallic Cu-based systems. Moreover, this preparation method is flexible and easy to achieve electrode scale-up. When a 4 cm^2^ electrode was applied to the MEA system, the corresponding full-cell E.C.E. could be increased to 40.5 and 26.8%, respectively, without compromising product selectivity. When the electrode area of the MEA system is increased to 25 cm^2^, the single-pass yield of C2+ products can be further increased to 13.2%, with an ethylene yield of 12.0%. In situ ATR-SEIRAS studies and theoretical calculations provide insights into the role of Cu(100) facets for increasing *CO coverage and reducing the energy barrier of C−C coupling, vital for ethylene and C2+ products formation. The potential of using this Cu(100)-rich film for photosynthesis was also demonstrated using renewable electricity generated by Si solar panels, achieving a solar-to-C2+ products efficiency of ~6.0% under simulated 1 Sun illumination. Future research endeavors may focus on the generation of energetic particles (atoms, molecules, ions, etc.) in other environments such as solutions in ambient conditions, which may further reduce the cost of one-step facet control by this vacuum-based deposition-etch-bombardment method.

## Methods

### Synthesis of LS-Cu, MS-Cu, HS-Cu

The deposition-etch-bombardment process was conducted in a custom-designed radio frequency (RF, 13.56 MHz) magnetron sputtering system (Supplementary Fig. 1a). For LS-Cu, Ar was delivered into the system. The deposition chamber was pumped down by a mechanical pump and a molecular pump that resulted in a base pressure of 2.0 × 10^−4^ Pa. The flow rate of Ar was set as 20 standard cubic centimeters per minute (sccm). During the deposition, the RF power was 40 W, and the working pressure was 4 Pa. The deposition time was 15 min. The target to substrate distance was set to 7 cm. Commercial GDLs were cut into squares (5 × 5 cm) for use as substrates. For MS-Cu, the RF power was set as 100 W and the deposition time was 7.5 min, other conditions were the same as LS-Cu. For HS-Cu, the RF power was set as 200 W and the deposition time was 3.5 min, other conditions were the same as LS-Cu.

### Synthesis of LRS-Cu, MRS-Cu, and HRS-Cu

During the synthesis process of precatalysts of LRS-Cu, MRS-Cu, and HRS-Cu, O_2_ was also introduced to the Ar atmosphere. The flow rates of Ar and O_2_ were 20 and 4 sccm, respectively, to achieve a partial pressure of O_2_ of 0.67 Pa. Other conditions were the same as those of LS-Cu, MS-Cu, and HS-Cu. The obtained precatalysts were pre-reduced at −0.5 V versus the RHE for 1 h under the same conditions as CO_2_ electroreduction. Pre-reduction was carried out with a potentiostat (CompactStat.e20250, IVIUM). After pre-reduction, the final samples (i.e., LRS-Cu, MRS-Cu, and HRS-Cu) were obtained.

### Synthesis of W-Cu

The W-Cu was fabricated through the electrochemical pre-reduction of the Cu_2_O nanorods at −0.5 V versus the RHE for 1 hour under the same conditions as CO_2_ electroreduction. After pre-reduction, the electrode was directly used for CO_2_ reduction. The Cu_2_O nanorods were prepared by annealing the Cu(OH)_2_ nanorods in the Ar atmosphere at 500 °C for 2 h with a heating rate of 10 °C min^−1^. The Cu(OH)_2_ nanorods were prepared by a previous method with some modifications^32^. The obtained Cu_2_O nanorods were airbrushed onto the commercial GDLs at an approximate loading of 0.9 mg/cm^2^, measured through weighing GDLs before and after airbrushing. The catalyst ink was prepared by dispersing 200 mg of Cu_2_O nanorods and 50 μL of Nafion Solution (Sigma-Aldrich) in 750 μL isopropyl alcohol and 250 μL of ultra-purity water (18.2 MΩ cm) and sonicated for 1 h before airbrushing (H-SET, Paasche). Pre-reduction was carried out with a potentiostat (CompactStat.e20250, IVIUM).

### Characterizations

Field-emission scanning electron microscopy (FESEM) (Hitachi S-4800, 3 kV) was used to characterize the morphology and microstructure of the samples. Transmission electron microscopy (TEM), High-resolution TEM (HRTEM) images were obtained at 200 kV (JEOL JEM-2100F). The crystal structure was determined by X-ray Diffractometer (XRD, Bruker D8 Focus) with Cu Kα radiation (λ = 1.54056 Å) at 40 kV and 40 mA. XRD spectra were collected over a 2θ range of 30−60° at a scanning speed of 8°/min. XPS analyses of precatalysts were carried out on a Physical Electronics PHI 1600 ESCA system with an Al Kα X-ray source (1486.6 eV). The binding energy was calibrated using the C 1s photoelectron peak at 284.6 eV as the reference.

### In-situ Raman spectroscopy measurements

In-situ Raman spectroscopy was carried out in a custom-designed flow cell (Supplementary Fig. 12a), which was manufactured by Gaossunion Co., Ltd., Tianjin. The electrode was encased in a PEEK fitting, with an exposed circular geometric surface area of ~1 cm^2^. A platinum wire and an Ag/AgCl electrode (saturated KCl, Gaossunion Co., Ltd., Tianjin) were used as the counter and the reference electrode, respectively. The counter electrode is separated from the working electrode by an anion exchange membrane (FAA-3-PK-75, Fumatech) to avoid cross-contamination. In situ Raman spectroscopy was performed with a Raman microscopy system (LabRAM HR Evolution, Horiba Jobin Yvon). A He−Ne laser (λ = 532 nm) served as the excitation source. All spectra were collected at a constant potential (−0.5 V versus the RHE). Electrochemical measurements were carried out with a potentiostat (CompactStat.e20250, IVIUM).

### In-situ ATR-SEIRAS measurements

In-situ ATR-SEIRAS was performed with an ATR configuration. Au nanofilms were deposited directly on the reflecting plane of a Si prism using a modified electroless chemical deposition method outlined by Xu et al.^58^. The spectroelectrochemical cell was based on the design of Xu et al.^59^ and manufactured by Gaossunion Co., Ltd., Tianjin. In order to reduce the corrosion of Si crystal, 0.1 M KOH was used as the electrolyte. The counter electrode (a graphite rod) was separated from the working and reference electrodes, i.e., the catalyst film and a saturated Ag/AgCl electrode (saturated KCl, Gaossunion Co., Ltd., Tianjin), respectively, with a piece of anion exchange membrane (AEM, FAA-3-PK-75, Fumatech). This cell is integrated into the FTIR (is50, Nicolet) spectrometer with a modified accessory at a 60° incident angle (VeeMax III, PIKE Technology). All spectra were collected with a 4 cm^−1^ resolution. Spectra are presented in absorbance, with positive and negative peaks showing an increase and decrease in signal, respectively. As for LRS-Cu, MRS-Cu and HRS-Cu, they were deposited on the Au nanofilm coated-Si prisms like the process described above, while W-Cu was drop-casted onto the Au nanofilm coated-Si prisms. The background was taken at +0.1 V versus the RHE in Ar saturated electrolyte for each electrode. Electrochemical measurements are carried out with a potentiostat (CompactStat.e20250, IVIUM).

### OH^−^ electroadsorption measurements

In-situ OH_ads_ studies were conducted by flowing Ar in the flow cell (Supplementary Fig. 15). First, CO_2_ electrolysis was conducted at a constant potential of −0.5 V versus the RHE for 1 h by switching the gas feed to CO_2_ and flowing the electrolyte. Immediately after electrolysis, the electrolyte (1 M NaOH (a.q.)) flow rate was stopped to minimize the fluctuation in the voltammogram, and the gas feed was switched to Ar, the electrolyte flow rate was stopped, and then cyclic voltammetry (20 mV/s) was performed. Electrochemical measurements are carried out with a potentiostat (Autolab PGSTAT204, Metrohm).

### ECSA measurements

The ECSA was determined by measuring the double-layer capacitance (C_DL_) of various electrodes in Ar-purged 2 M KOH (aq.) in the flow cell (Supplementary Fig. 15) and the ECSA was measured after CO_2_ electrolysis at a constant potential of −0.5 V versus the RHE for 1 h. Immediately after electrolysis, the gas feed was switched to Ar, and then the electrolyte flow rate was stopped to minimize the fluctuation in the voltammogram. The scan rate was varied from 25 to 125 mV s^−1^ in the non-faradaic potential region and the observed current was plotted as a function of scan rate to obtain the C_DL_. ECSA was determined by normalizing the C_DL_ to that of a Cu foil. Electrochemical measurements are carried out with a potentiostat (CHI 660E, CH Instruments Inc.).

### Electrochemical reduction of CO_2_ in a flow cell

CO_2_ reduction was conducted in a custom-designed three-chamber flow cell manufactured by Gaossunion Co., Ltd. (Supplementary Fig. 15), where the CO_2_ gas was supplied directly to the catalyst layer (cathode, working electrode). The CO_2_ gas flow rate was controlled using a mass flow controller (MC-Series, Alicat Scientific) and set to 10 sccm. However, it is well known that OH^−^ can react with CO_2_ to form HCO_3_^−^or CO_3_^2−^. Therefore, the calculation based on the inlet CO_2_ flow rate will result in overestimated F.E. results^60,61^. For this reason, we used another flowmeter (M-Series, Alicat Scientific) to detect the CO_2_ flow rate at the outlet of the reactor and used this number as the basis for calculating F.E. Aqueous KOH solution (2 M) was used as both the catholyte and the anolyte. Activated Ni foam was used as the anode (counter electrode). Peristaltic pumps (EC200-01, Gaossunion Co., Ltd.) were used to control the flow rate of the electrolytes at ~10 ml min^−1^. An AEM (FAA-3-PK-75, Fumatech) was used to separate the cathode and anode chambers. Electrolysis experiments were conducted using chronoamperometry with a potentiostat (CompactStat.e20250, IVIUM). The cathode potentials were measured against a Hg/HgO reference electrode (1 M KOH, Gaossunion Co., Ltd., Tianjin). For each measurement, products were quantified after the amount of electron flowing through the cathode achieved 50 C and at least three replicates were conducted to obtain an average value with the standard deviation. It should be noted that iR correction was not performed.

### Electrochemical reduction of CO_2_ in the MEA system

The MEA cell (manufactured by Gaossunion Co., Ltd.) consists of a titanium anode (cathode) bipolar plate with serpentine flow field, associated nuts, bolts, and insulating kit. The geometric area of each flow field is 4 or 25 cm^2^ (Supplementary Fig. 40). An AEM membrane (FAA-3-PK-75, Fumatech) was activated in 0.1 M KOH for 24 h, washed with ultra-purity water prior to use. The anode consisted of iridium oxide supported on titanium mesh (IrOx/Ti mesh) was prepared by a dip-coating and thermal decomposition method^62^. The MEA was assembled in a way as illustrated in Fig. 5a. A direct current power supply (UTP1300, UNI-T Group Co., Ltd) was used to apply current to the MEA. A Corrtest CS350M in a galvanostatic mode was used to measure the cell voltage. No iR compensation was applied. Aqueous KHCO_3_ electrolyte (0.1 M) was used as the anolyte and was circulated using a peristaltic pump (EC200-01, Gaossunion Co., Ltd.). The electrolyte flow rate was kept at 10 mL min^–1^. As the current density and electrode area increase, the CO_2_ flow rate should be adjusted upwards to avoid mass transfer limitations of CO_2_ while maintaining optimum selectivity. The flow rate of the CO_2_ gas flowing into the cathode flow field was kept at 20 or 60 sccm by a mass flow controller (MC-Series, Alicat Scientific) for different geometries of the flow field. CO_2_ was flowed through a homemade humidifier (7/8 full of Milli-Q water, room temperature) prior to the MEA. The flow rate of the CO_2_ gas flowing out the cathode flow field was also measured by a flowmeter (M-Series, Alicat Scientific). The liquid products carried by CO_2_ gas are absorbed by low-temperature ultra-purity water obtained from an ice salt bath

### Analysis of CO_2_ reduction products

During electrolysis, gas products were quantified using an on-line gas chromatography system (GC7890B, Agilent Technologies, Inc.). The thermal conductivity detector (TCD) connected to a MolSieve 5A packed column (Agilent Technologies, Inc.) to detect H_2_, O_2_, and N_2_ and a back flame ionization detector (FID) connected to a Porapak Q packed column (Agilent Technologies, Inc.) to detect CO. A methanizer was installed to enable the back FID to detect CO with 1000 times higher sensitivity. A front FID connected to an HP-PLOT Al_2_O_3_ capillary column (Agilent Technologies, Inc.) to detect hydrocarbons (C1~C3). Ar was used as the carrier gas. After passing through the reactor, the gas was allowed to flow directly into the gas sampling loop of the gas chromatography for online gaseous product analysis.

In the performance test using flow cell and the MEA system, the liquid products were collected from the cathode and anode chambers^60^. The liquid products were analyzed by headspace gas chromatography (HS-GC) and ^1^H-NMR. HS-GC measurements were carried out using a BCHP HS-2 Headspace Sampler with GC2060 gas chromatography (Shanghai Ruimin Instrument Co., Ltd.). Typically, 10 mL vials were filled with 3 mL of the liquid sample and sealed. They were heated to 70 °C over 20 min in the headspace sampler and 1 mL of the headspace gas composition was automatically injected into the GC. The sample loop (110 °C) and transfer line (110 °C) were both heated to avoid condensation. Ar was used as the carrier gas. An HP-INNOWax capillary column (Length: 60 m; ID: 0.32 mm; Film: 0.5 μm, Agilent) was used to separate the compounds in the sample. ^1^H-NMR was performed using AVANCE IIITM HD 400 MHz NanoBAY. The water suppression method was used. DMSO (10 mM) and phenol (50 mM) were added as internal standards. For CO_2_ reduction products showing multiple sets of NMR peaks, the set of peaks with the highest intensity were chosen for calibration and quantification.

### Construction of the photovoltaic-electrolyzer (PV-EC) system

The simulated solar illumination was obtained from a 300 W Xenon arc lamp (Microsolar 300 UV, Beijing Perfectlight Technology Co. Ltd.) equipped with an air mass 1.5 global (AM 1.5G) filter, and the power intensity of the incident light was calibrated to 100 mW/cm^2^ using a Si photodiode (FDS100, Thorlabs). The solar panel was based on five p−n^+^ Si solar cells connected in series (effective illuminated area of ~5.7 cm^2^). A Source Measure Unit (2450, Keithely) was wired in series with 0 V applied to monitor the current. The electrolyzer is the flow cell.

### Computational methods

Vienna ab initio simulation package (VASP) was used to carry out calculations with the PBE exchange-correlation functional^63,64^. Van der Waals interactions were accounted for by using the DFT-D3 method^65^. The cut-off energy is 400 eV. The interactions between the atomic cores and electrons were described by the projector augmented wave (PAW) method^66^. All structures were optimized until the force on each atom has been less than 0.02 eV/Å. The transition state search was conducted with the climbing image nudged elastic band (CI-NEB) method, followed by the dimer method to converge the saddle point within 0.05 eV/Å. We access CO dimerization on the three models. A four-layer Cu(111)-(3×3) slab with a (3×3×1) k-point grid and a four-layer Cu(100)-(3×3) slab with a (3×3×1) k-point grid were used as models for DFT calculations. The bottom two layers are fixed while the upper two layers were relaxed during optimization. One layer of water with a simple hydronium ion was chosen to simulate the electrochemical interface^67–69^.

## Supplementary information

Supplementary Information

## Data Availability

The authors declare that all data supporting the results of this study are available within the paper and its supplementary information files or from the corresponding authors upon reasonable request.
