# Supplementary material for: Efficient CO2 electroreduction on facet-selective copper films with high conversion rate
Source: Nat Commun. 2021 Sep 30;12:5745. doi: 10.1038/s41467-021-26053-w (PMC8484611; doi:10.1038/s41467-021-26053-w)
Supplement: Supplementary file 1 — Supplementary Information [file 41467_2021_26053_MOESM1_ESM.docx]

**Supplementary Information**

**Efficient CO_2_ Electroreduction on Facet-selective Copper Films with High Conversion Rate**

Gong Zhang,^1,2,3‡^ Zhi-Jian Zhao,^1,2,3‡^ Dongfang Cheng,^1,2,3^ Huimin Li,^1,2,3^ Jia Yu,^1,2,3^ Qingzhen Wang,^1,2,3^ Hui Gao,^1,2,3^, Jinyu Guo,^4^ Huaiyuan Wang,^1,2,3^ Geoffrey A. Ozin,^5^ Tuo Wang,^1,2,3*^ and Jinlong Gong^1,2,3,6*^

^1^School of Chemical Engineering and Technology, Tianjin University, Tianjin 300072, China

^2^Key Laboratory for Green Chemical Technology of Ministry of Education, Tianjin University, Tianjin 300072, China.

^3^Collaborative Innovation Center of Chemical Science and Engineering (Tianjin), Tianjin 300072, China.

^4^ Department of Chemical Engineering, Stanford University, Stanford, California 94305, United

States

^5^ Department of Chemistry, University of Toronto, 80 St. George Street, Toronto, Ontario M5S 3H6, Canada.

^6^Joint School of National University of Singapore and Tianjin University, International Campus of Tianjin University, Binhai New City, Fuzhou 350207, China.

^‡^These authors contributed equally to this work.

^*^email: [jlgong@tju.edu.cn](mailto:jlgong@tju.edu.cn); wangtuo@tju.edu.cn

**Contents**

1. Supplementary Methods

2. Supplementary Figures and Tables

3. Supplementary References

**Supplementary Methods**

**Materials**

Cu target (99.999%) was supplied by Zhongnuo Advanced Material Technology Co., Ltd, China. NiFe foam was purchased from Tianjin Incole Union Technology Co., Ltd. Commercially available carbon-based gas diffusion layers (GDLs, AvCarb GDS3250) were purchased from Xima Laya Photo-Electric Technology Co., Ltd., China. KOH (GR, 95%), NaOH (AR, 96%), KHCO_3_ (AR, 99.5%), oxalic acid (AR, 98%), isopropanol (HPLC) were purchased from Sigma-Aladdin. Cu(NO)_3_ (98%) was purchased from J&K Scientific Ltd. NaOH (GR) and NH_3_·H_2_O (GR) were both purchased from Tianjin Kemiou Chemical Reagent Co., Ltd. HCl (GR) was purchased from Damao Chemical Reagent Factory. IrCl_3_·xH_2_O (99.8%) was purchased from Alfa Aesar. The reagents were used without any purification process. Ultra-purity water (18.25 MΩ·cm) supplied by a UP Water Purification System was used in the whole experimental process. CO_2_, CO, Ar and O_2_ were all supplied by Air Liquide (≥99.999%).

**Predictions of the possible Tafel slopes and reaction orders of ethylene (C_2_H_4_)**

Different rate-determining steps are summarized in the table below. The symmetry factor β in Tafel slope calculations is assumed to be 0.5. Detailed derivations and analysis are also presented.

Tafel slopes and reaction orders of C_2_H_4_ formation

| RDS | CO reaction order | | Tafel slope |
| --- | --- | --- | --- |
|  | low coverage | high coverage | mV/dec |
| *CO + *CO + *e^−^*→*C_2_O_2_^−^ + * | 2 | 0 | 118 |
| *CO + *CO(g) + *e^−^*→*C_2_O_2_^−^ + * | 2 | 1 | 118 |

1. Langmuir-Hinshelwood Step

Under this assumption, the RDS is the coupling between two *CO.

RDS: *CO + *CO + *e^-^*→*C_2_O_2_^−^ + *

The rate expression can be written as:

*j*_C2H4_ = *k_1_θ_CO_*^2^exp($\frac{\text{-βEF}}{\text{RT}})$ (E3-1)

In addition, in all cases the Tafel slope can be defined as^1, 2^:

Tafel Slope = $\frac{\text{∂η}}{\text{∂}\text{lgj}_{\text{C2H4}}}$ =$\frac{\text{∂(}\text{E}^{\text{rev}}\text{-}\text{E)}}{\text{∂lg}\text{j}_{\text{C2H4}}}$ = −$\frac{\text{2.3}}{\frac{\text{∂ln}\text{j}_{\text{C2H4}}}{\text{∂E}}}$

Where:

$\frac{\text{∂ln}\text{j}_{\text{C2H4}}}{\text{∂E}}$ =$\frac{\text{-}\text{βF}}{\text{RT}}$ , *β* = 0.5

Thus:

Tafel Slope = $\frac{\text{2.3}\text{RT}}{\text{βF}}$ = 118 mV/dec

Assuming a fast equilibrium for CO adsorption on the surface, where:

CO(g) + * → *CO

Then,

*K_CO_* =$\frac{\text{θ}_{\text{CO}}}{\text{P}_{\text{CO}}\text{θ}_{\text{*}}}$ (E3-2)

According to the reports of Bell *et al*., under the reaction conditions, the surface CO coverage of the catalyst is very high (~1), while the coverage of other species is extremely low (~10^−4^)^3^, so we can ignore the coverage of other species. Then:

*θ** ≈ 1 − *θ_CO_* (E3-3)

Combining equations (E1-3) and (E1-2) yields:

*θ_CO_* = $\frac{\text{K}_{\text{CO}}\text{P}_{\text{CO}}}{\text{1}\text{ }\text{+}\text{ }\text{K}_{\text{CO}}\text{P}_{\text{CO}}}$ (E3-4)

*K_CO_P_CO_* = $\frac{\text{θ}_{\text{CO}}}{\text{1}\text{ - }\text{θ}_{\text{CO}}}$ (E3-5)

Then E1-1 can be rewritten as:

*j*_C2H4_ = *k_1_*exp($\frac{\text{-}\text{βEF}}{\text{RT}})\left( \frac{\text{K}_{\text{CO}}\text{P}_{\text{CO}}}{\text{1}\text{ }\text{+}\text{ }\text{K}_{\text{CO}}\text{P}_{\text{CO}}} \right)$^2^ (E3-6)

a) According to equation 1-5, at low *CO coverage (i.e., *θ_CO_*→0) or low CO partial pressure (i.e., *P_CO_*→0), then *K_CO_P_CO_* approaches 0, and the equation E1-6 can be expressed as:

*j*_C2H4_ = *k_1_*exp($\frac{\text{-}\text{βEF}}{\text{RT}})$(*K_CO_P_CO_*)^2^ (E3-7)

Therefore, it means a second-order dependence of the reaction rate on the pressure of CO.

b) At high *CO coverage (i.e., *θ_CO_* → 1) or high CO partial pressure, then *K_CO_P_CO_* >> 1, and the equation E1-6 can be expressed as:

*j*_C2H4_ = *k_1_*exp($\frac{\text{-}\text{βEF}}{\text{RT}})$ (E3-8)

Therefore, it means a zero-order dependence of the reaction rate on the pressure of CO.

2. Eley-Rideal Step

Under this assumption, the RDS is the coupling between *CO and CO(*g*).

RDS: *CO + CO(*g*) + *e^−^*→*C_2_O_2_^−^ + *

The rate expression can be written as:

*j*_C2H4_ = *k_2_θ_CO_P_CO_*exp($\frac{\text{-}\text{βEF}}{\text{RT}})$ (E4-1)

Then,

Tafel Slope = $\frac{\text{2.3}\text{RT}}{\text{βF}}$= 118 mV/dec

Assuming a fast equilibrium for CO adsorption on the surface, where:

CO(g) + * → *CO

Then,

*K_CO_* = $\frac{\text{θ}_{\text{CO}}}{\text{P}_{\text{CO}}\text{θ}_{\text{*}}}$ (E4-2)

Similarly, we have:

*θ** ≈ 1 − *θ_CO_* (E4-3)

*θ_CO_* = $\frac{\text{K}_{\text{CO}}\text{P}_{\text{CO}}}{\text{1}\text{ }\text{+}\text{ }\text{K}_{\text{CO}}\text{P}_{\text{CO}}}$ (E4-4)

*K_CO_P_CO_* = $\frac{\text{θ}_{\text{CO}}}{\text{1}\text{-}\text{θ}_{\text{CO}}}$ (E4-5)

Then E2-1 can be rewritten as:

*j*_C2H4_ = *k_2_*exp($\frac{\text{-}\text{βEF}}{\text{RT}})\frac{\text{K}_{\text{CO}}\text{P}_{\text{CO}}^{\text{2}}}{\text{1}\text{ }\text{+}\text{ }\text{K}_{\text{CO}}\text{P}_{\text{CO}}}$ (E4-6)

a) At low *CO coverage (i.e., *θ_CO_* → 0) or low CO partial pressure (i.e., *P_CO_* → 0), the equation E2-6 can be expressed as:

*j*_C2H4_ = *k_1_*exp($\frac{\text{-}\text{βEF}}{\text{RT}})$*K_CO_P_CO_*^2^ (E4-7)

Therefore, it means a second-order dependence of the reaction rate on the pressure of CO.

b) At high *CO coverage (i.e., *θ_CO_* → 1) or high CO partial pressure, the equation E2-6 can be expressed as:

*j*_C2H4_ = *k_1_*exp($\frac{\text{-}\text{βEF}}{\text{RT}})$*P_CO_* (E4-8)

Therefore, it means a first-order dependence of the reaction rate on the partial pressure of CO.

**Calculations of the full-cell electricity conversion efficiency (E.C.E.)**

E.C.E.is calculated as follows:

E.C.E*.* _=_ $\sum_{\text{X}} \frac{\text{E}^{\text{0}}\left( \text{X} \right)\text{ }\text{×}\text{ }\text{F.E.(X)}}{\text{Cell Voltage}}$

Where *E^0^(X)* is the equilibrium potential for the overall electrochemical reaction for product X at standard conditions (Supplementary Table 6); and F.E.(X) is the Faradaic efficiency (F.E.) of product X. When the applied working electrode potential is −0.75 V vs. RHE, the corresponding cell voltage of flow cell is 2.72 V.

**Calculation of the solar conversion efficiency**

Solar conversion efficiency (*η_._*) is calculated as follows:

*η* _=_ $\frac{\text{E}^{\text{0}}\left( \text{X} \right)\text{ }\text{×}\text{ }\text{I}_{\text{cell}\text{ }}\text{×}\text{ }\text{F.E.(X)}}{\text{P}\text{ }\text{×}\text{ }\text{S}}$

Where *I_cell_* is the current passing through the electrolyzer; *S* is the effective illuminated area; *E^0^(X)* is the equilibrium potential for the overall electrochemical reaction for product X at standard conditions (Supplementary Table 6); F.E.(X) is the Faradaic efficiency of product X.

**Supplemental Figures and Tables**

**
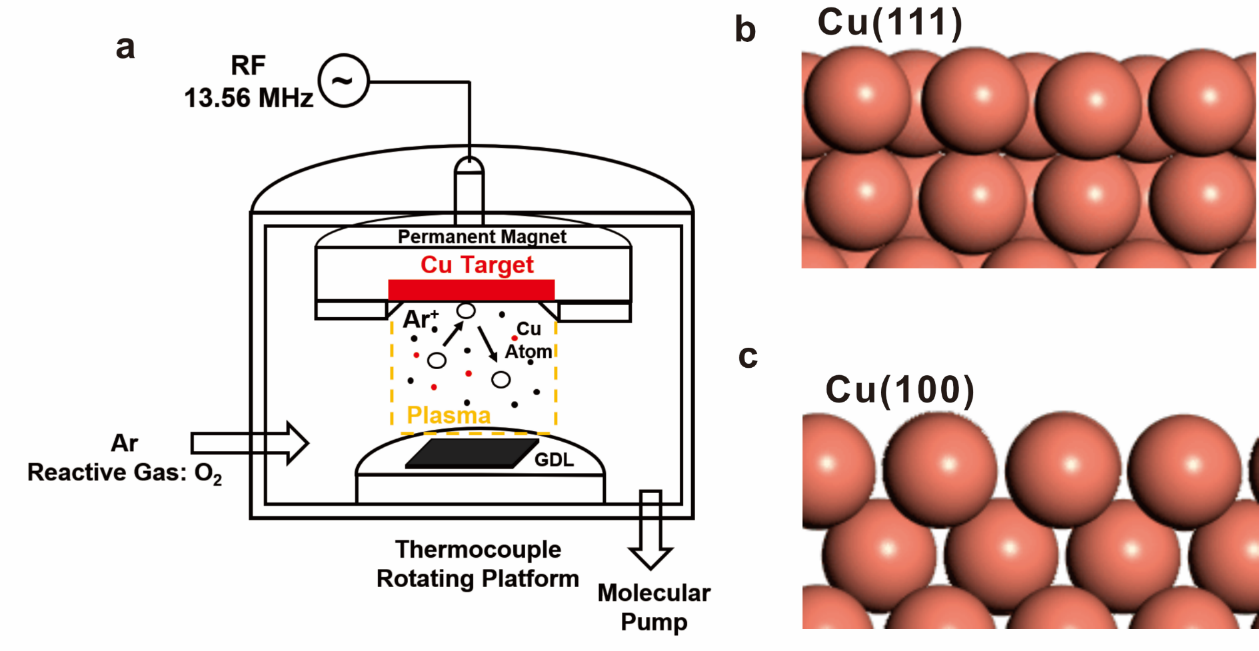
**

**Supplementary Fig. 1.** **Schematic diagram of catalyst preparation process.** **a** Schematic diagram of a reactive magnetron sputtering configuration. Projection of the **b** Cu(111) and **c** Cu(100) facet on the surface normal to the atom incident direction.


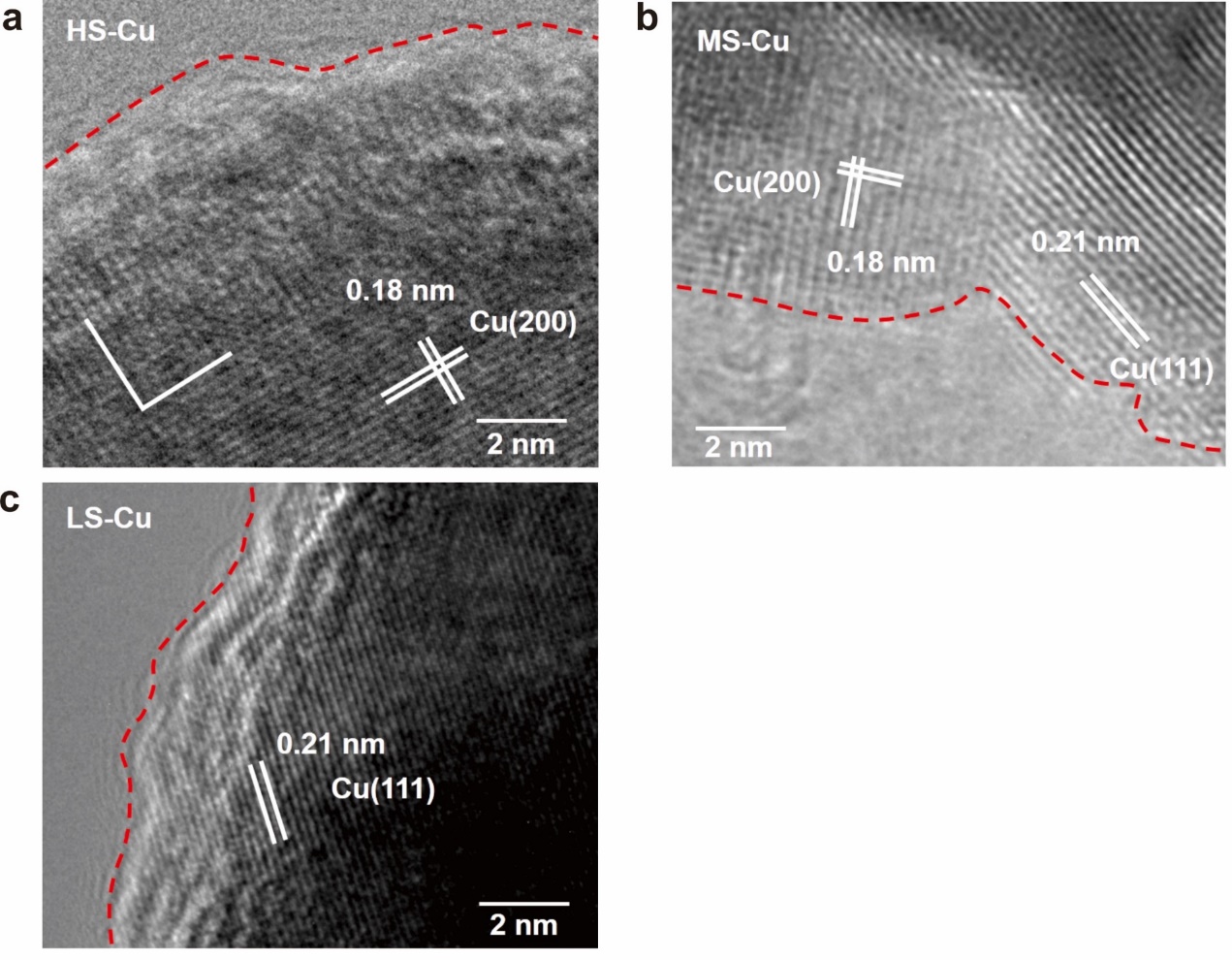


**Supplementary Fig. 2.** **HRTEM images corresponding to different samples.** **a** HS-Cu, **b** MS-Cu and **c** LS-Cu.

The red dash line indicates the rough surface. The labeling of Cu(200) is used for easier comparison with XRD, in which only (200), the second-order diffraction of (100) could be detected.

**
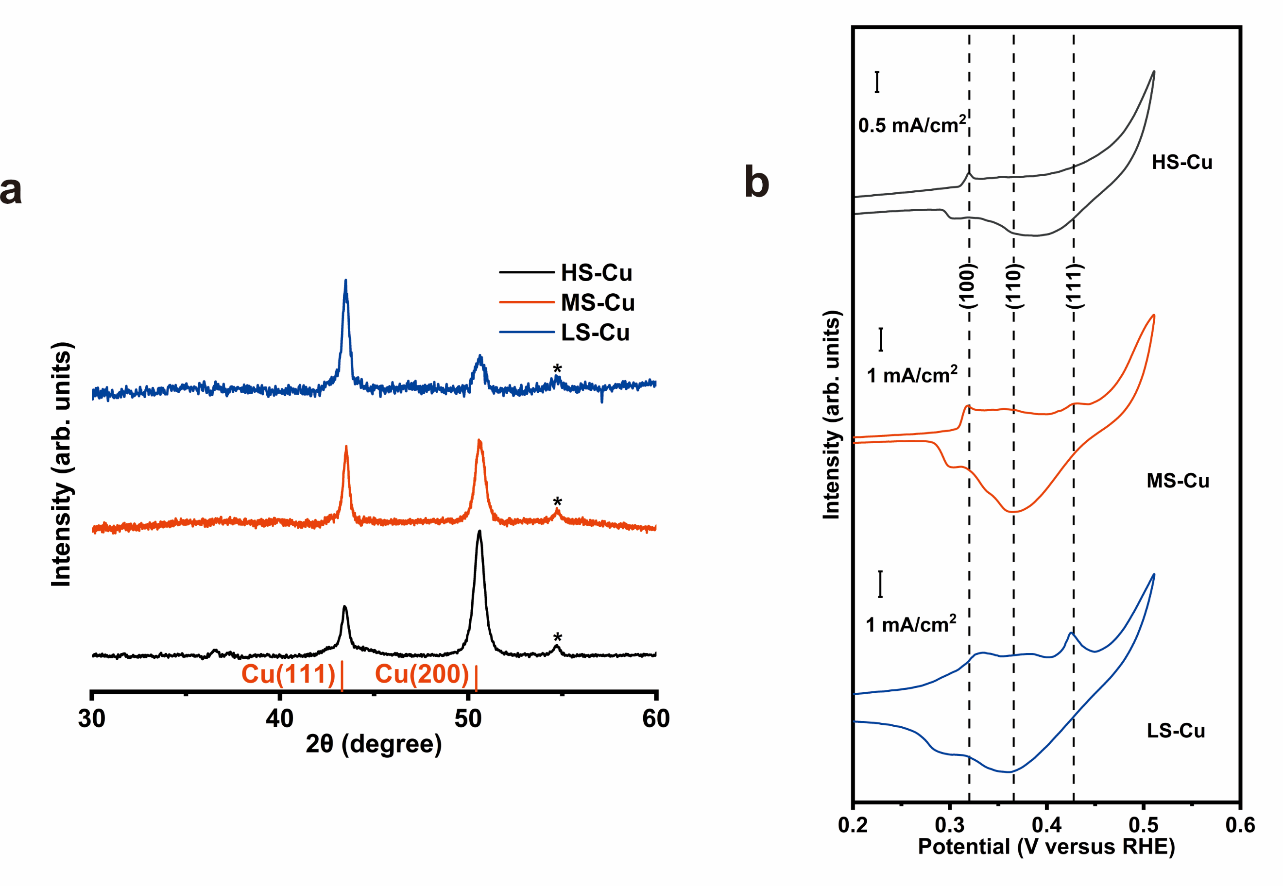
**

**Supplementary Fig. 3.** **Characterization of facet exposure of different samples.** **a** XRD patterns and **b** CVs of the resulting samples.

The peaks marked with an asterisk in the XRD patterns originate from the substrate.


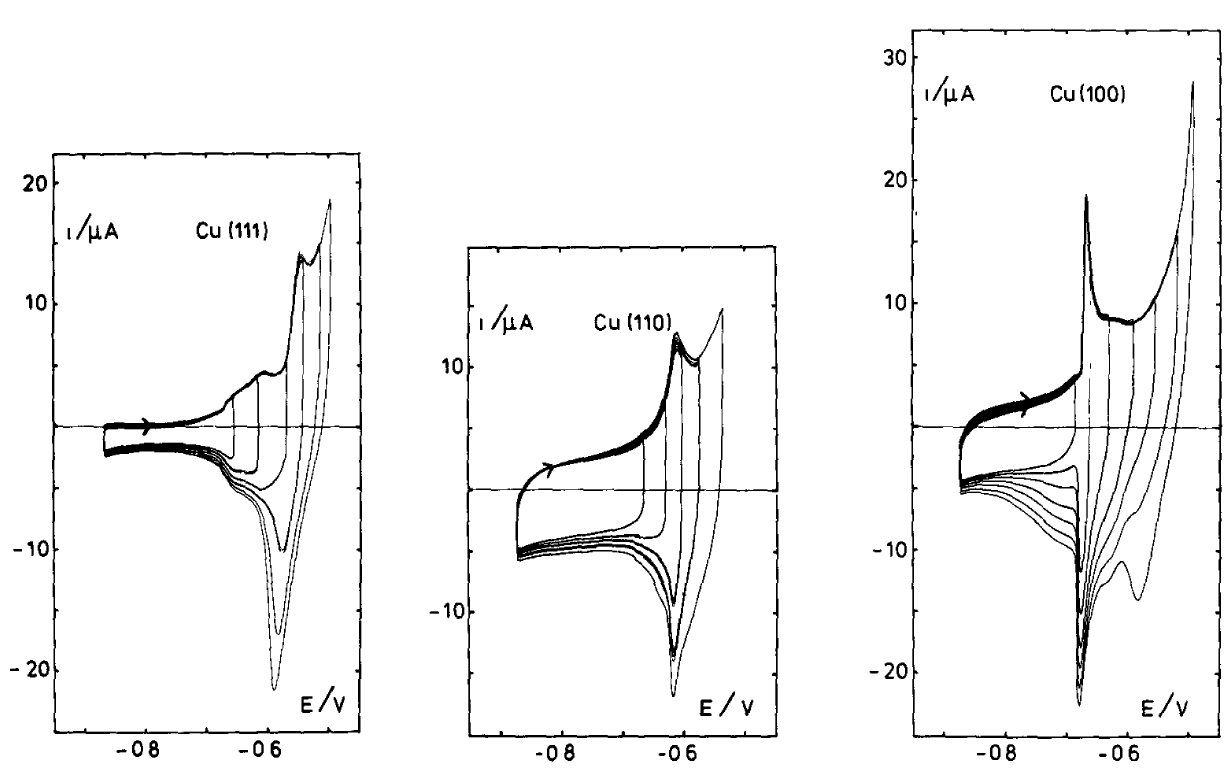


**Supplementary Fig. 4.** **Characteristic CV profiles for different Cu facets.**

The CVs of single-crystal Cu electrodes as reported by Droog and Schlenter.^4^ Recorded at 20 mV/s in 1 M NaOH. The potentials were given against Ag/AgCl.


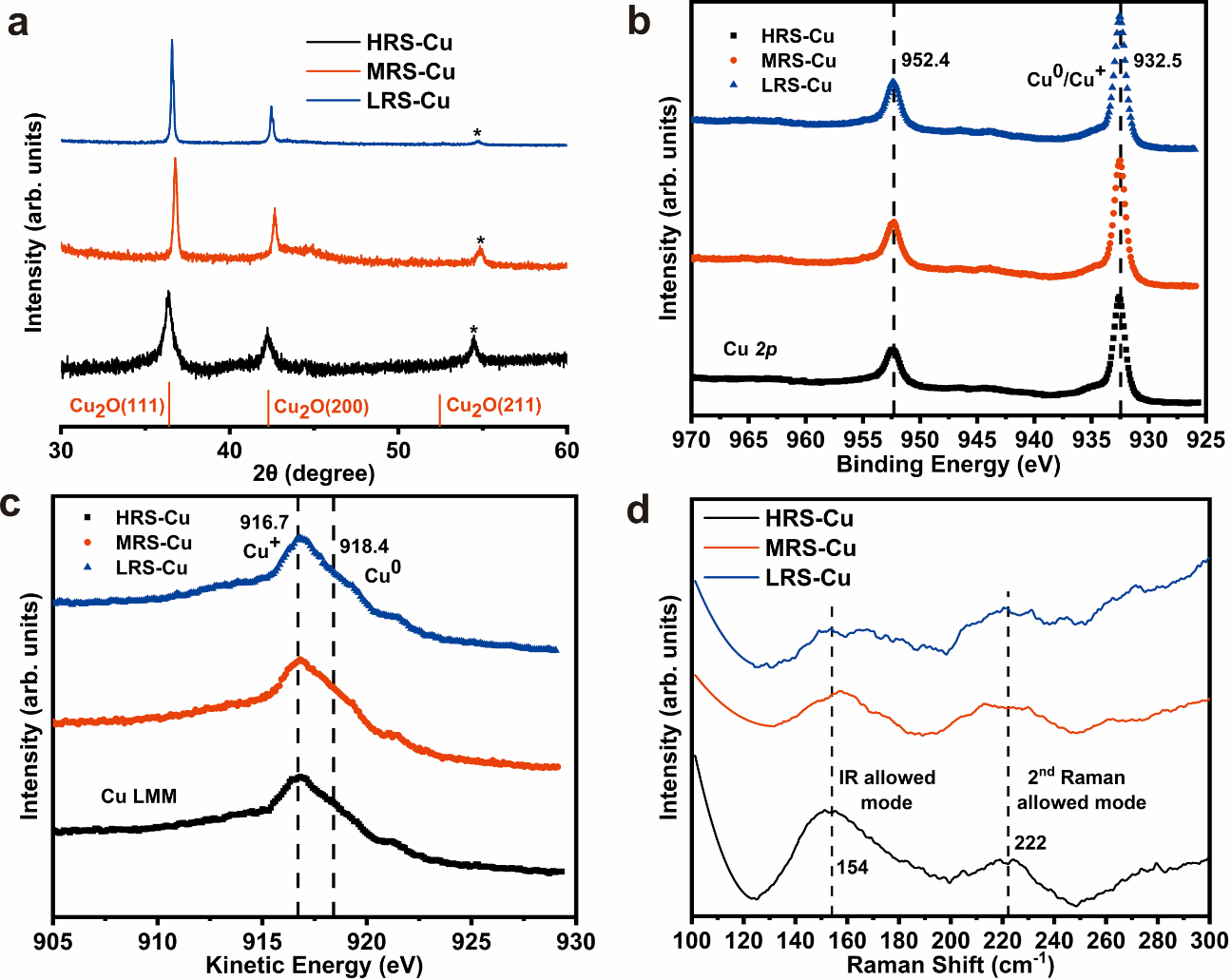


**Supplementary Fig. 5.** **Structural characterization of different precatalysts.** **a** XRD patterns, **b** Cu *2p* spectra, **c** Augar Cu LMM and (d) Raman spectra of precatalysts of the LRS-Cu, MRS-Cu and HRS-Cu.

The peaks marked with an asterisk in the XRD patterns originate from the substrate.

Supplementary Fig. 45 shows that these precatalysts only have the characteristic Raman shifts of Cu_2_O, indicating that the chemical composition of these precatalysts is Cu_2_O.

The precursor of HRS-Cu shows the highest XRD intensity ratio (about 0.5) of Cu_2_O(200)/Cu_2_O(111). According to previous studies,^19^ this phenomenon implies that the precursor of HRS-Cu may be preferentially exposed to Cu_2_O(100) facets, which would favorably produce Cu(100) facets during the reduction process.


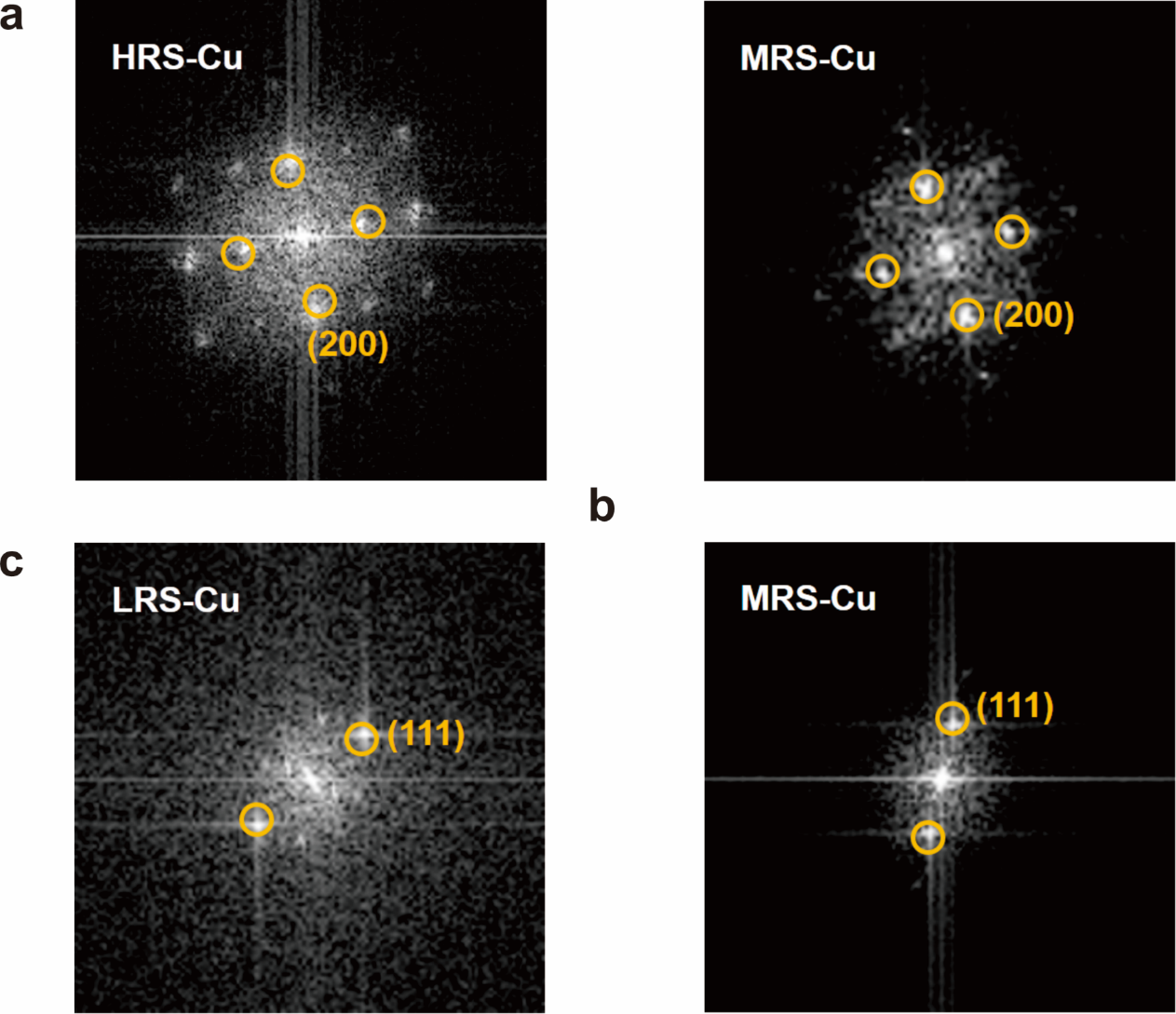


**Supplementary Fig. 6.** **Fast Fourier transforms (FFT) images of the obtained samples**.

The FFT image (related to Fig.1) shows that obtained HRS-Cu and LRS-Cu are mainly composed of one kind of lattice plane, i.e. Cu(200) and Cu(111) respectively, while obtained MRS-Cu is composed of lattice planes of Cu(200) and Cu(111) pairs, which is consistent with XRD results (Fig. 1b). All FFT images are with the same scale bar. FFT images were obtained with Gatan Digital Micrograph 3.9 software with default settings.


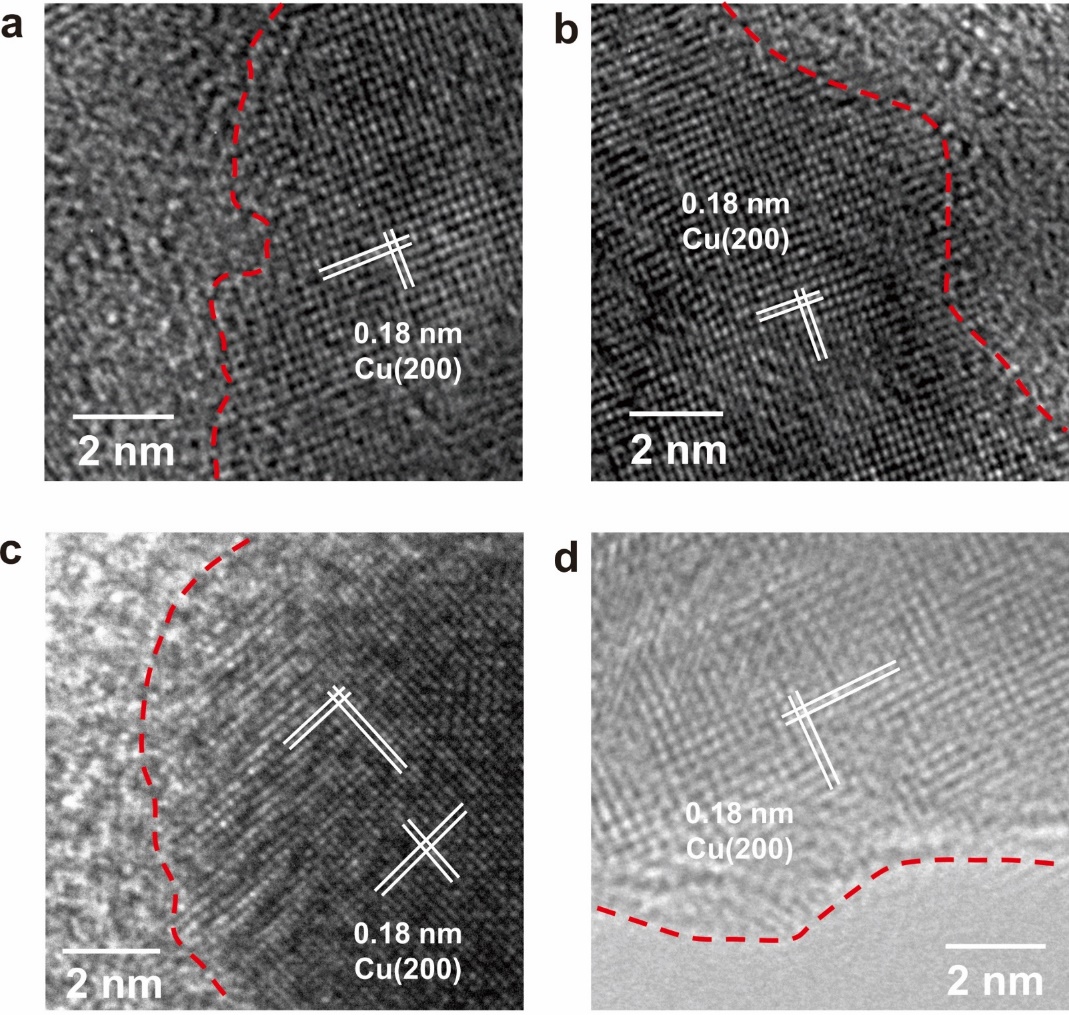


**Supplementary Fig. 7.** **Additional TEM images of obtained HRS-Cu.**

The red dash line indicates the rough surface. The labeling of Cu(200) is used for easier comparison with XRD, in which only (200), the second-order diffraction of (100) could be detected.


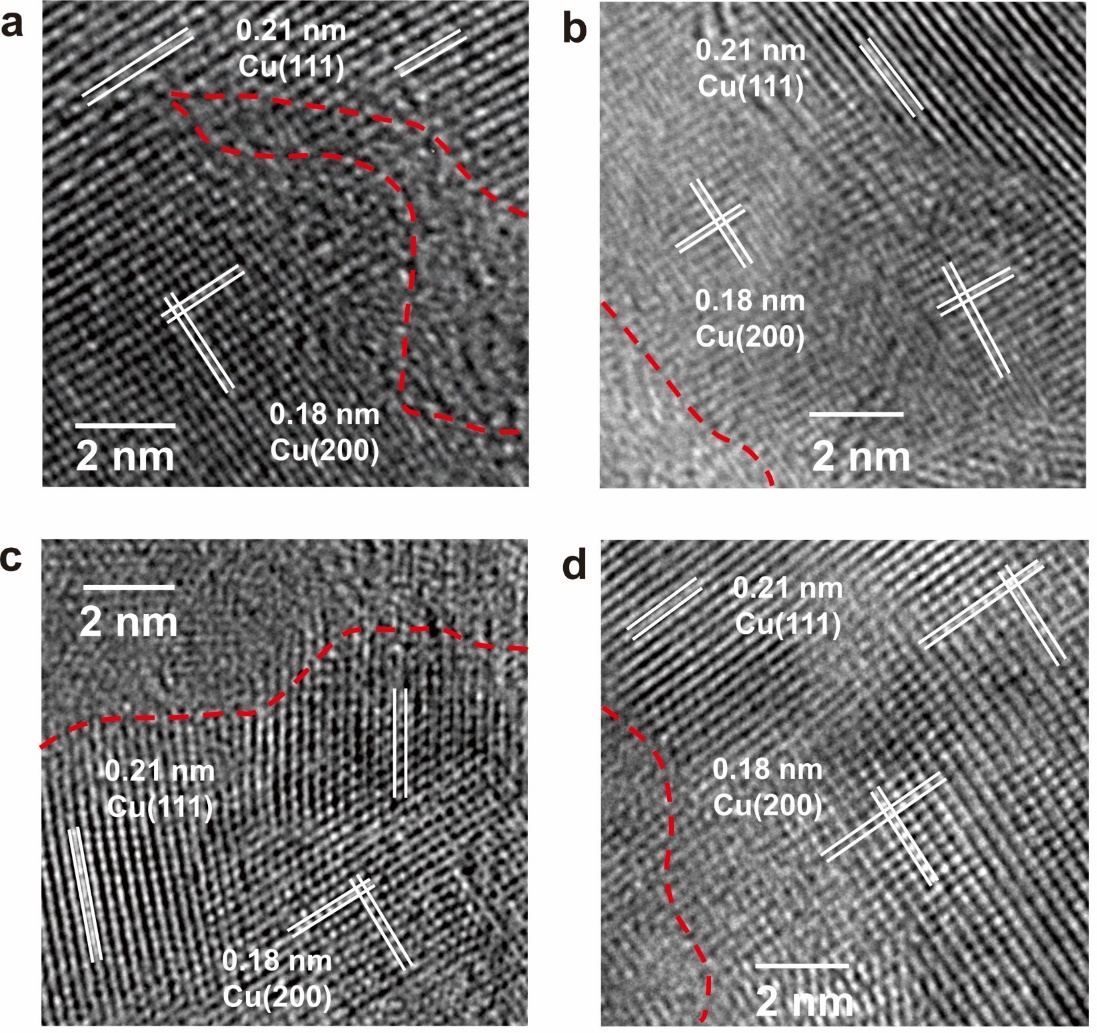


**Supplementary Fig. 8.** **Additional TEM images of obtained MRS-Cu.**

The red dash line indicates the rough surface. The labeling of Cu(200) is used for easier comparison with XRD, in which only (200), the second-order diffraction of (100) could be detected.


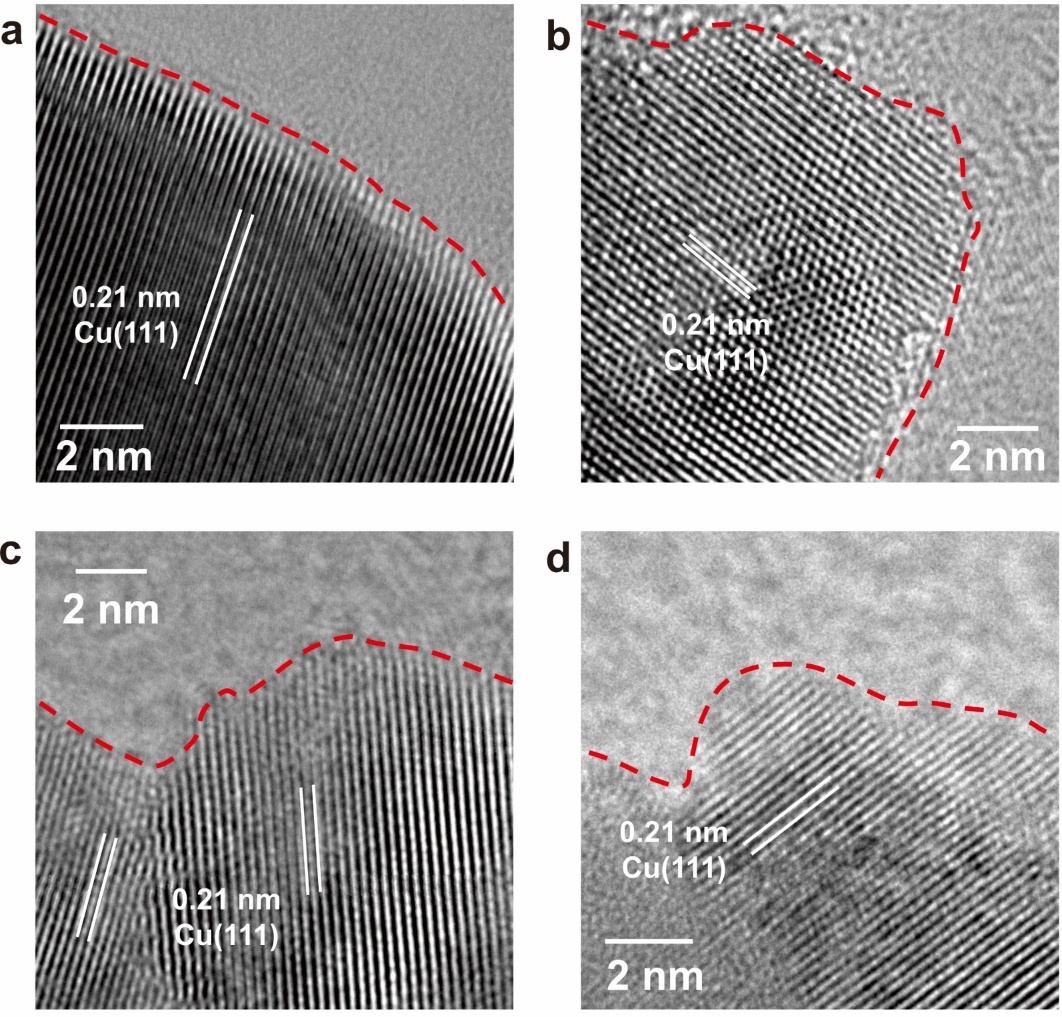


**Supplementary Fig. 9.** **Additional TEM images of obtained LRS-Cu.**

The red dash line indicates the rough surface. The labeling of Cu(200) is used for easier comparison with XRD, in which only (200), the second-order diffraction of (100) could be detected.


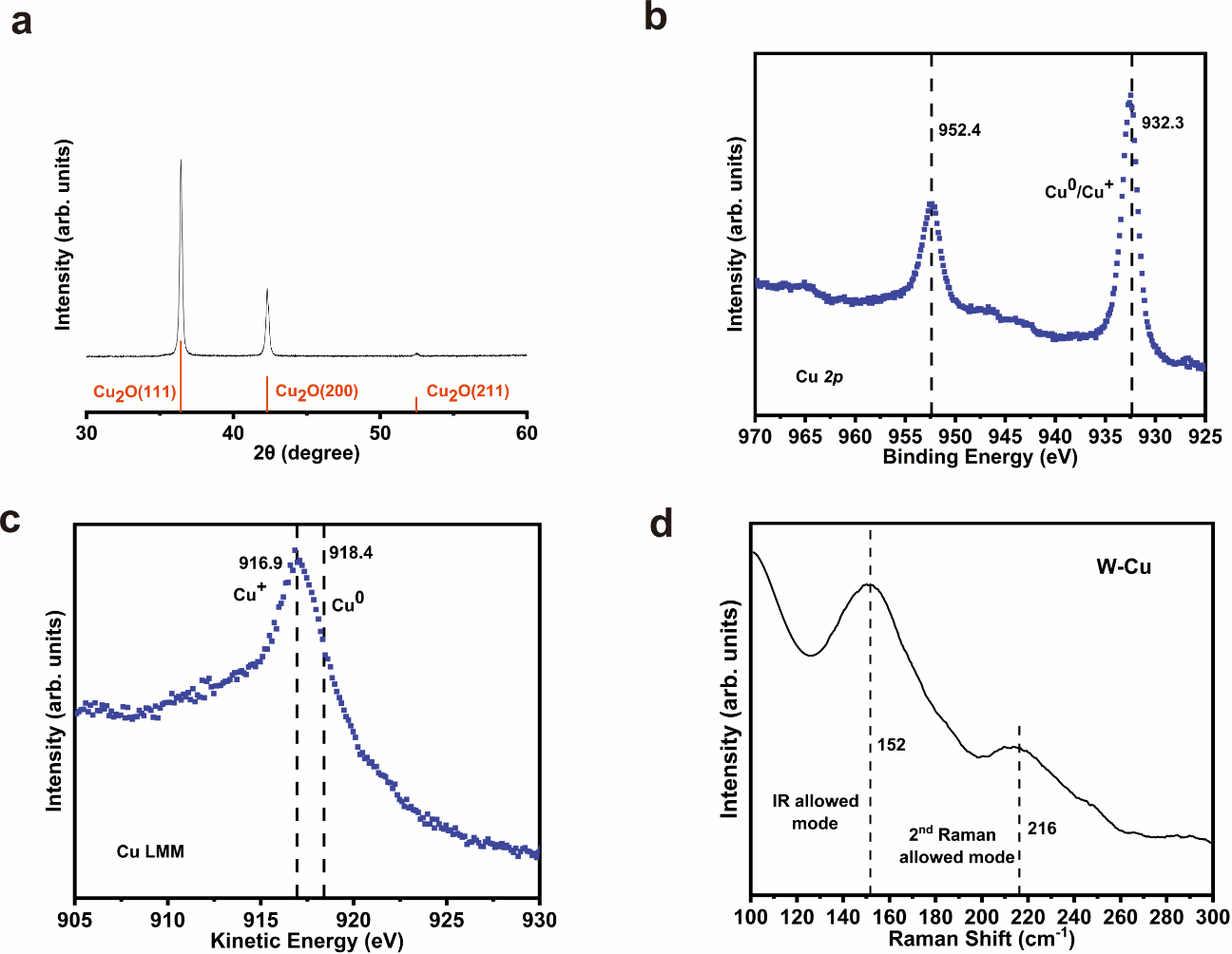


**Supplementary Fig. 10.** **Structural characterization of the precatalyst of the control sample.** **a** XRD pattern, **b** Cu *2p* spectrum, **c** Augar Cu LMM and **d** Raman spectrum of the precatalyst of the W-Cu.

Supplementary Fig. 9d shows that this precatalyst only has the characteristic Raman shifts of Cu_2_O, indicating that the chemical composition of this precatalyst is Cu_2_O.


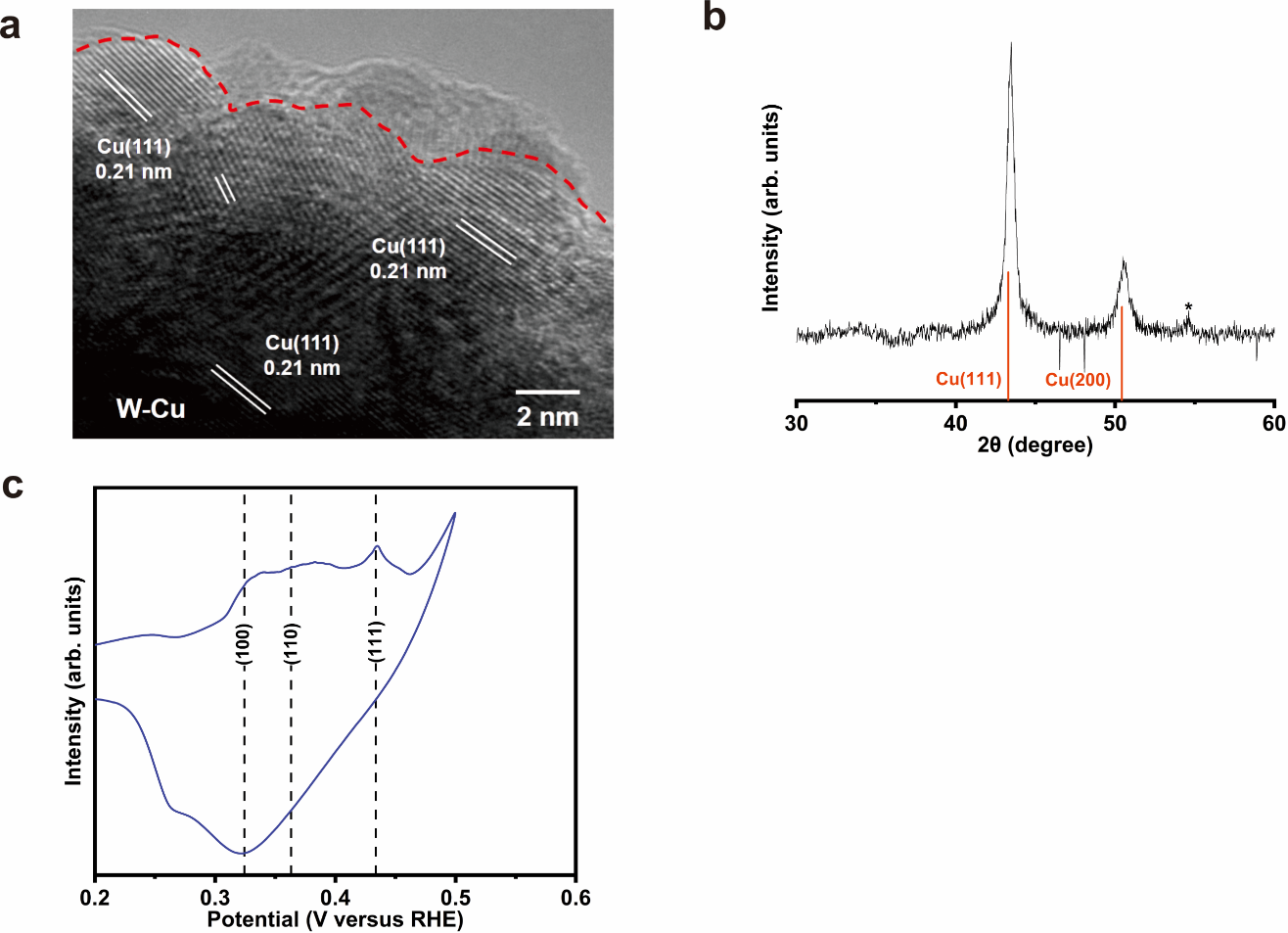


**Supplementary Fig. 11.** **Structural characterization of the obtained control sample.** **a** XRD pattern and **b** CV of the obtained W-Cu.

The red dash line indicates the rough surface. The peak marked with an asterisk in the XRD pattern originates from the substrate.


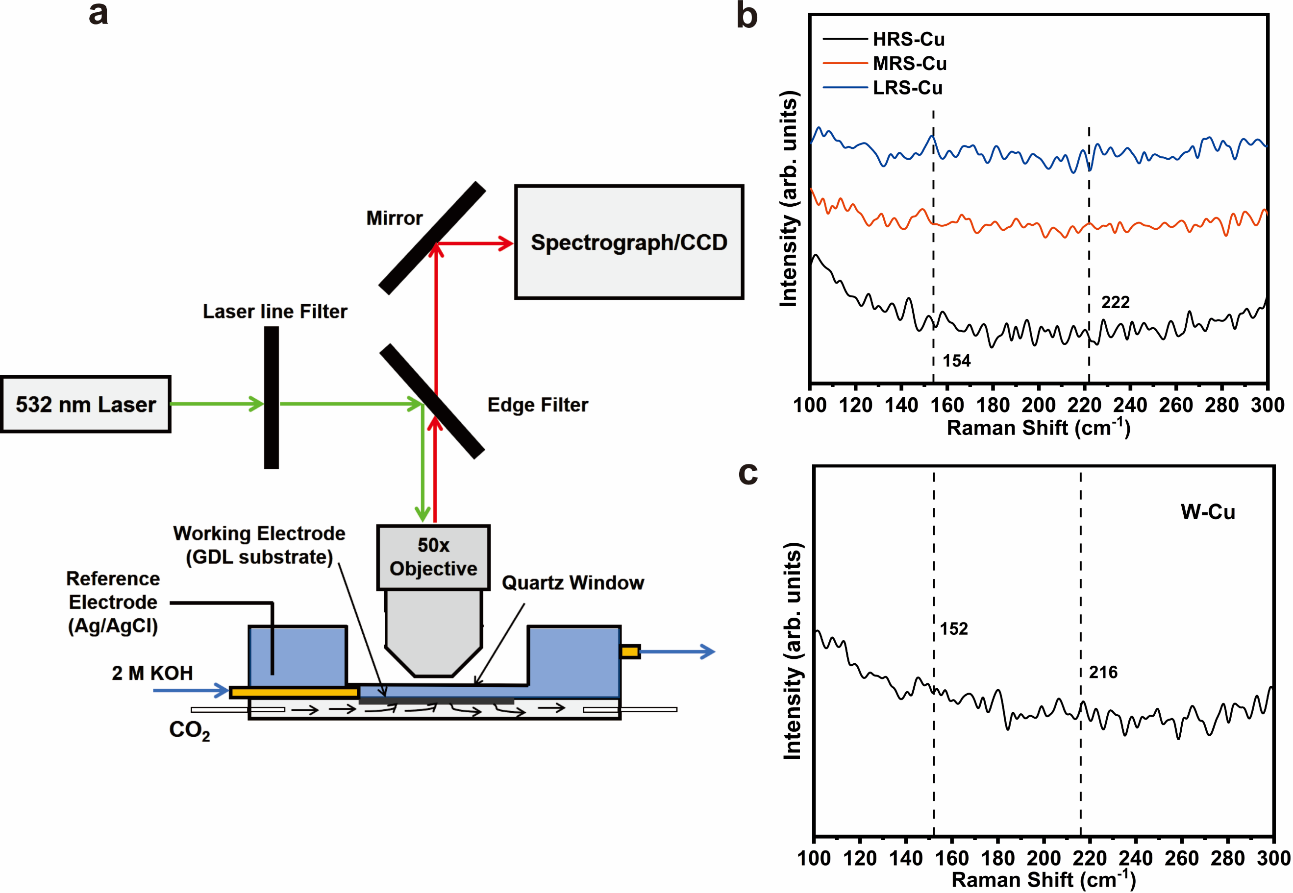


**Supplementary Fig. 12.** **Schematic diagram of in-situ Raman test and Raman spectra of different samples.** **a** Raman flow Cell design with side views of the cathode area; in-situ Raman spectra of the **b** LRS-Cu, MRS-Cu, HRS-Cu and **c** W-Cu.

The black dotted lines show the Raman shifts of the precatalysts (Supplementary Fig. 5d and Supplementary Fig. 10d) corresponding to different samples. According to the results of in-situ Raman test during the pre-reduction process, the catalytic electrode materials (i.e., LRS-Cu, MRS-Cu, HRS-Cu and W-Cu) obtained by pre-reduction for CO_2_ reduction reaction exhibit no characteristic peaks of oxides, which indicates that these catalytic materials are all of metallic nature, with no residual oxides under the reaction conditions.


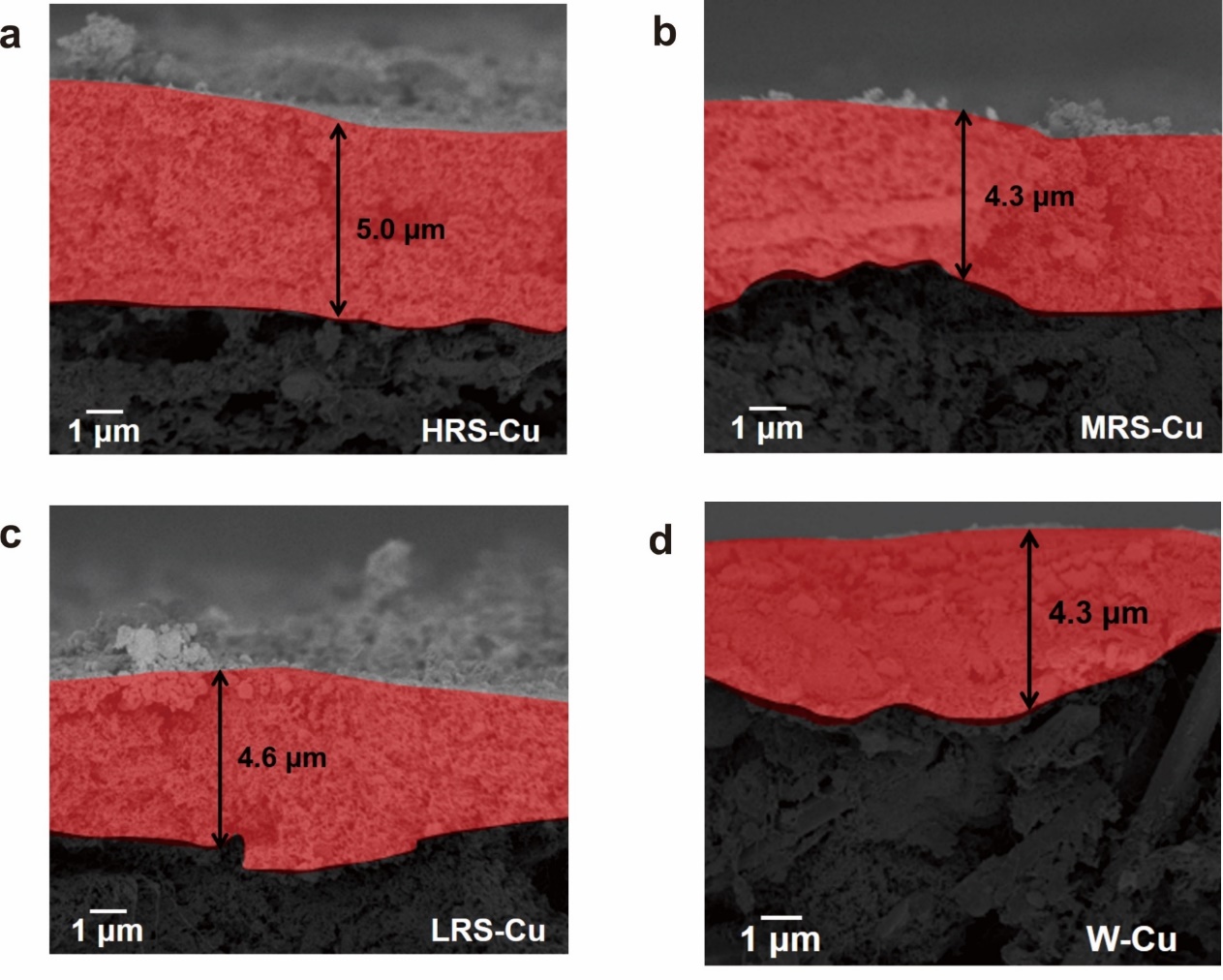


**Supplementary Fig. 13.** **Cross-sectional SEM images of different samples.** **a** HRS-Cu, **b** MRS-Cu, **c** LRS-Cu and **d** W-Cu.

False color was added to aid visualization of the layers. Red represents the catalyst layer, black represents the GDL substrate.


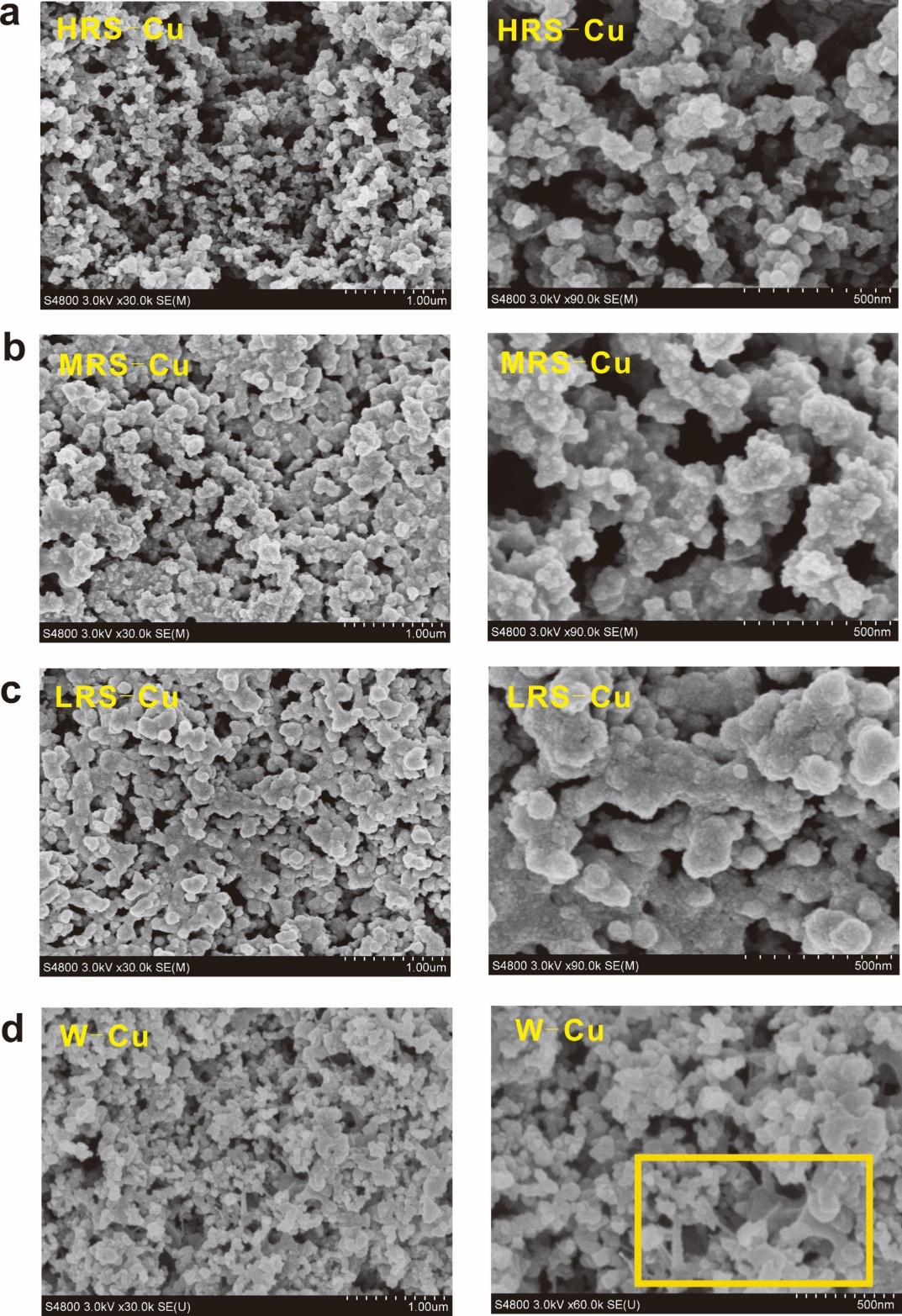


**Supplementary Fig. 14.** **Top-view SEM images of different samples.** **a** HRS-Cu, **b** MRS-Cu, **c** LRS-Cu and **d** W-Cu with different magnifications (30 and 90 k for left and right columns, respectively).

The image in the yellow rectangle in the right column shows the network structure formed by the polymer binder, where some catalyst materials are imbedded in it. In contrast, this phenomenon does not exist in the samples (i.e., HRS-Cu, MRS-Cu and LRS-Cu) obtained by the one-step dynamic deposition-etch-bombardment process.


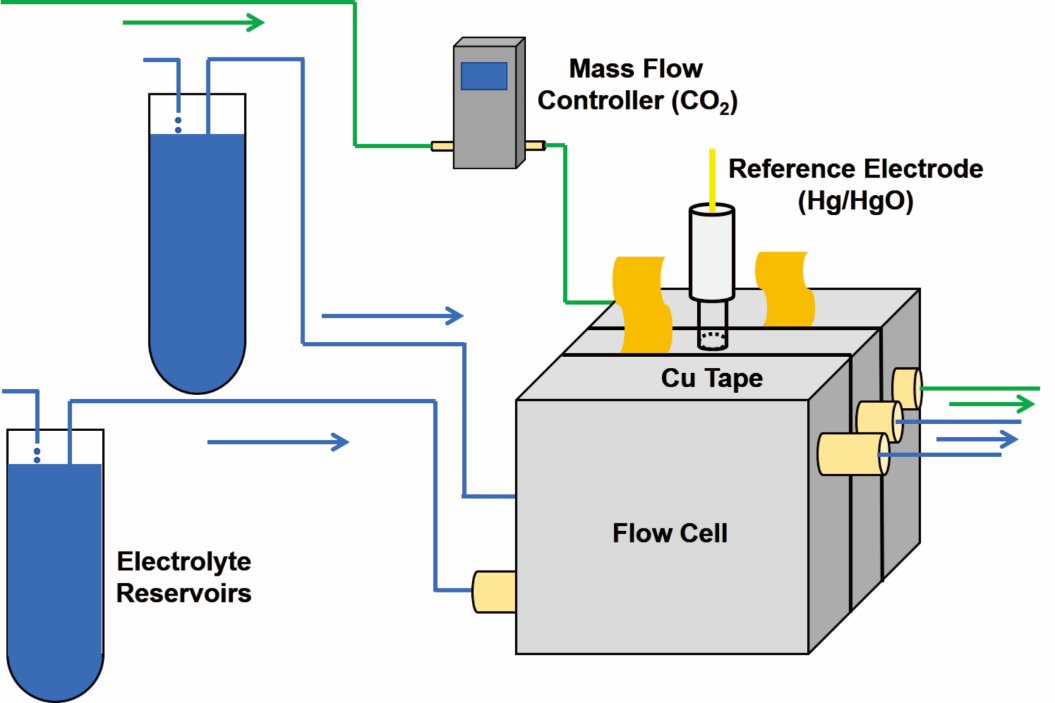


**Supplementary Fig. 15.** **Schematic diagram of CO_2_ reduction flow cell.**


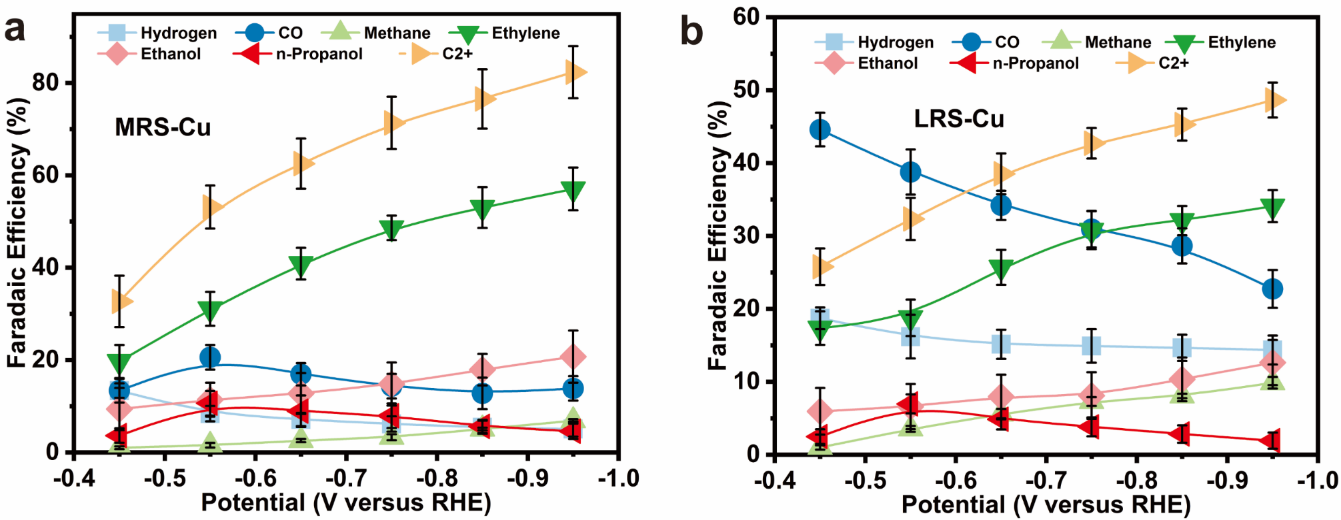


**Supplementary Fig. 16.** **Faradaic efficiencies of CO_2_ reduction products on different samples.** **a** MRS-Cu and **b** LRS-Cu as a function of different applied potentials.

Error bars represent the standard deviation from at least three independent measurements.


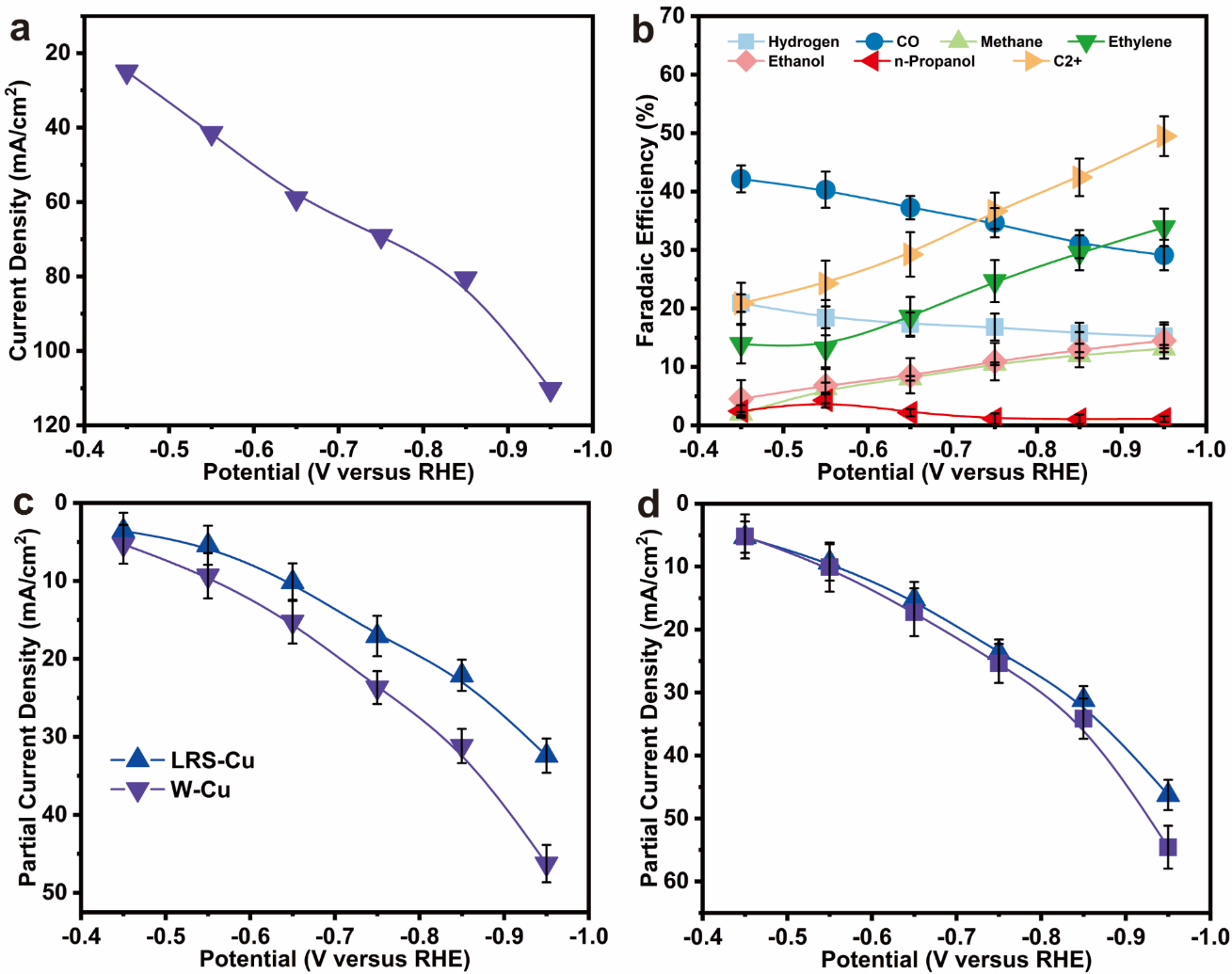


**Supplementary Fig. 17.** **CO_2_ reduction performance of the control sample.** **a** Overall current density, **b** Faradaic efficiencies of CO_2_ reduction products, **c** Ethylene and **d** C2+ products partial current densities on the W-Cu as a function of different applied potentials.

Error bars represent the standard deviation from at least three independent measurements.


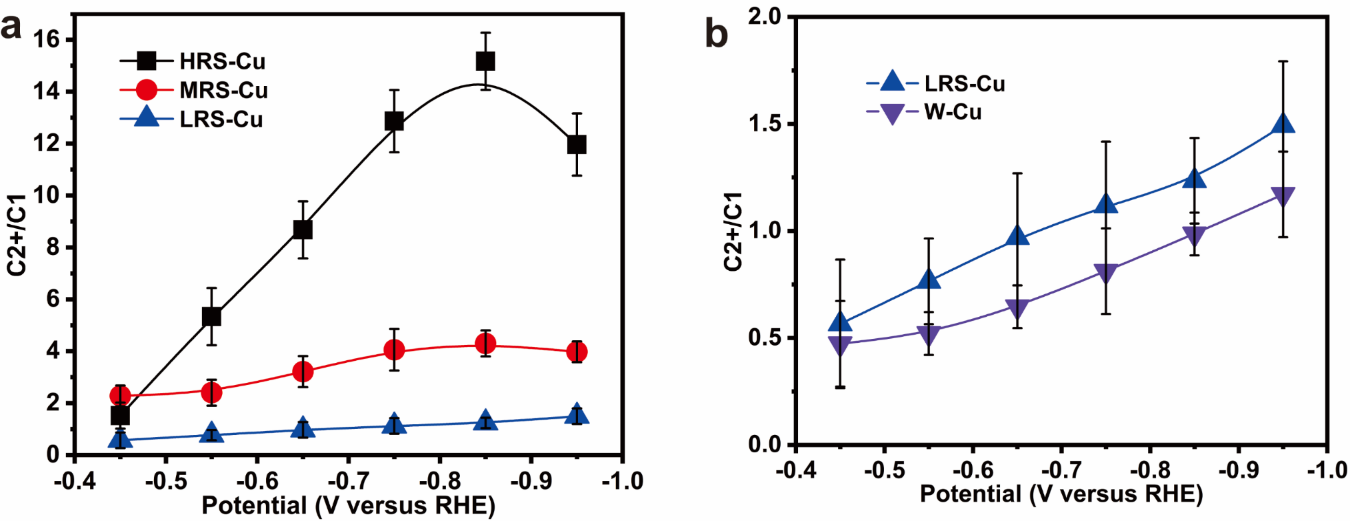


**Supplementary Fig. 18.** **C2+/C1 product selectivity on different samples.** **a** LRS-Cu, MRS-Cu, HRS-Cu and **b** W-Cu.

Error bars represent the standard deviation from at least three independent measurements.


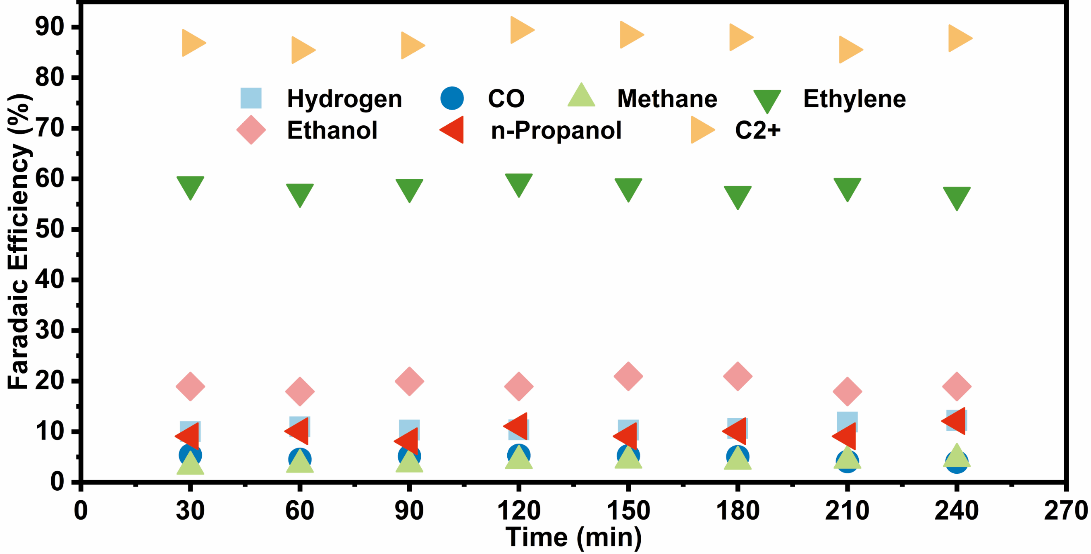


**Supplementary Fig. 19.** **Faradaic efficiencies of all CO_2_ reduction products on the HRS-Cu during the stability test.**


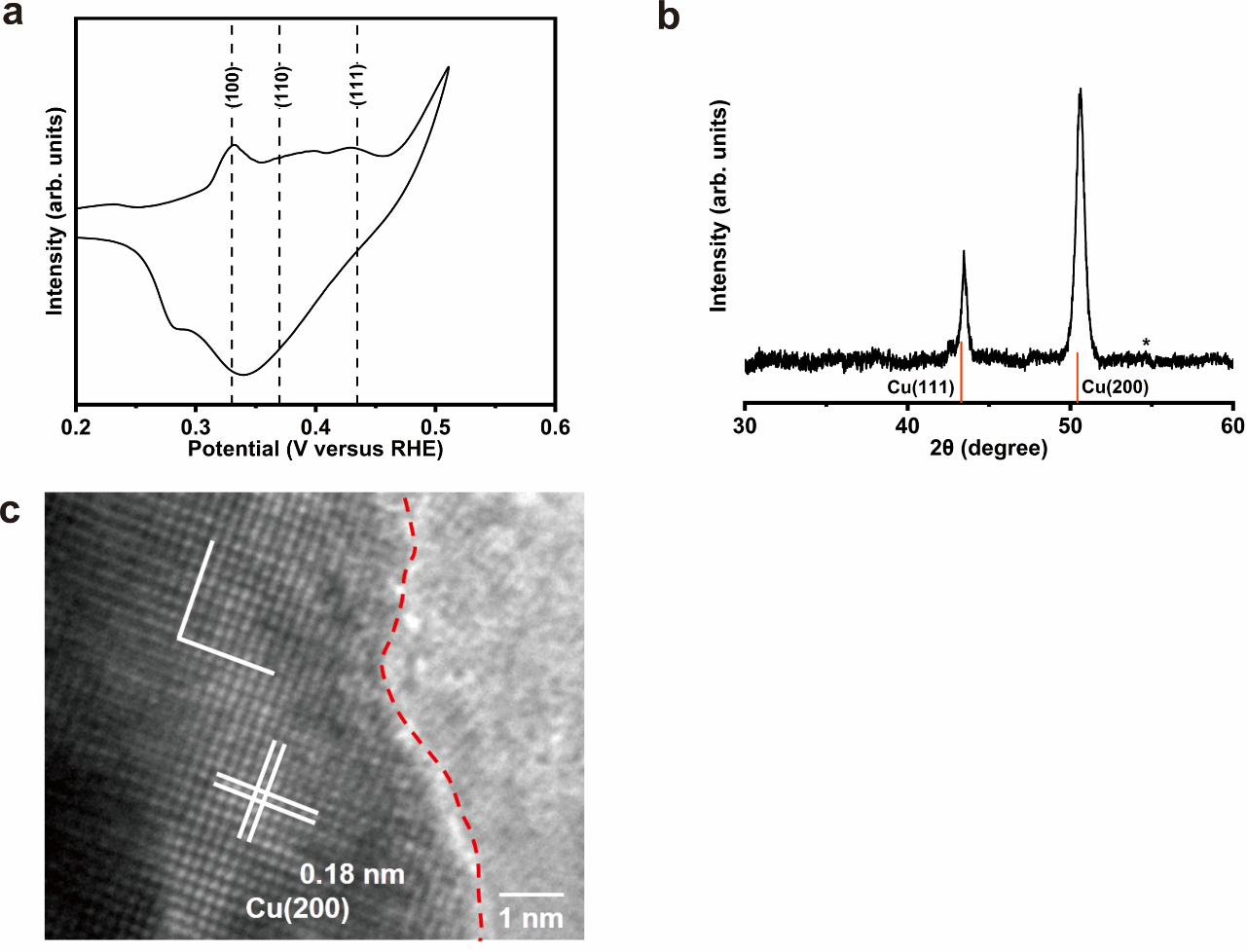


**Supplementary Fig. 20.** **Structural characterization of HRS-Cu after CO_2_ reduction.** **a** CV, **b** XRD pattern and **c** TEM image of HRS-Cu after CO_2_ reduction reaction.

The red dash line indicates the rough surface. The peak marked with an asterisk in the XRD pattern originates from the substrate.

**
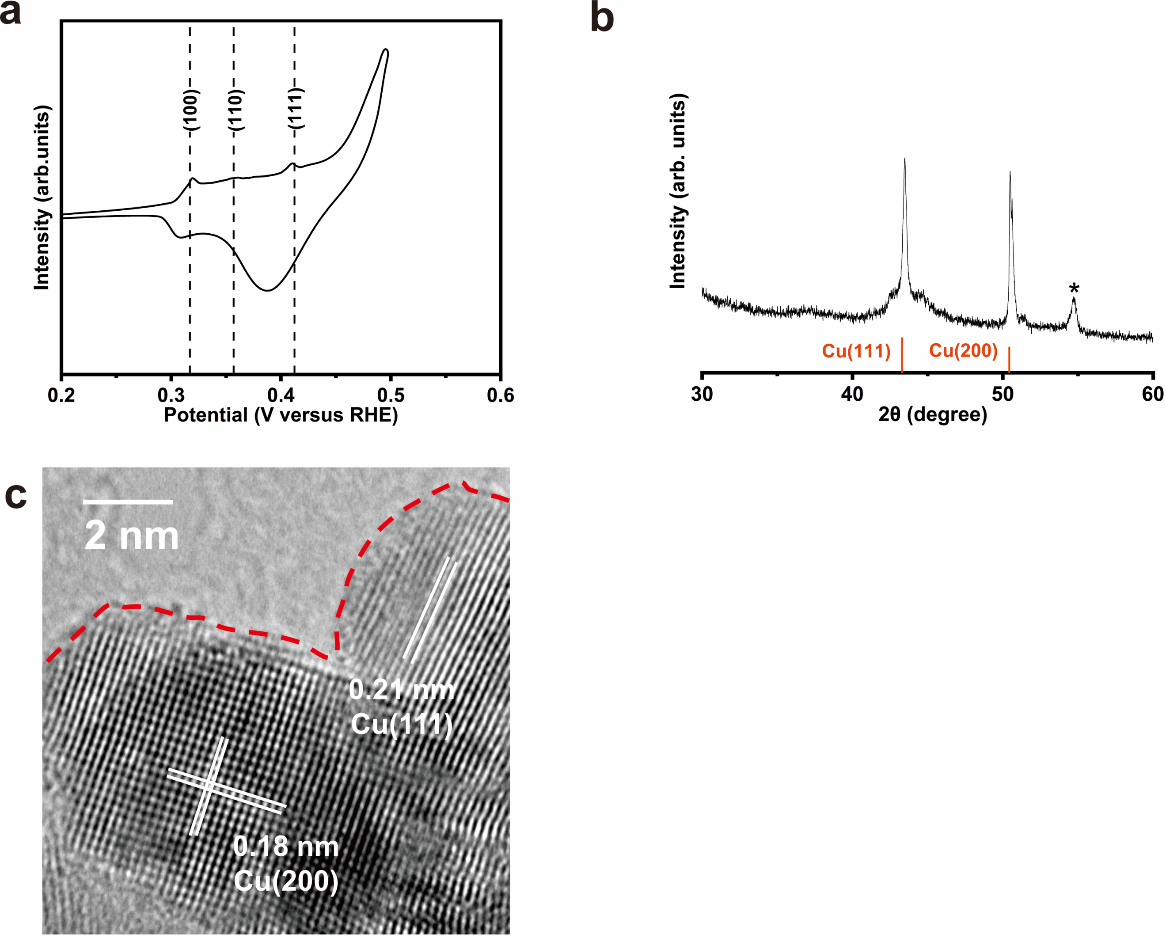
**

**Supplementary Fig. 21.** **Structural characterization of MRS-Cu after CO_2_ reduction. a** CV, **b** XRD pattern and **c** TEM image of MRS-Cu after CO_2_ reduction reaction.

The red dash line indicates the rough surface. The peak marked with an asterisk in the XRD pattern originates from the substrate.

**
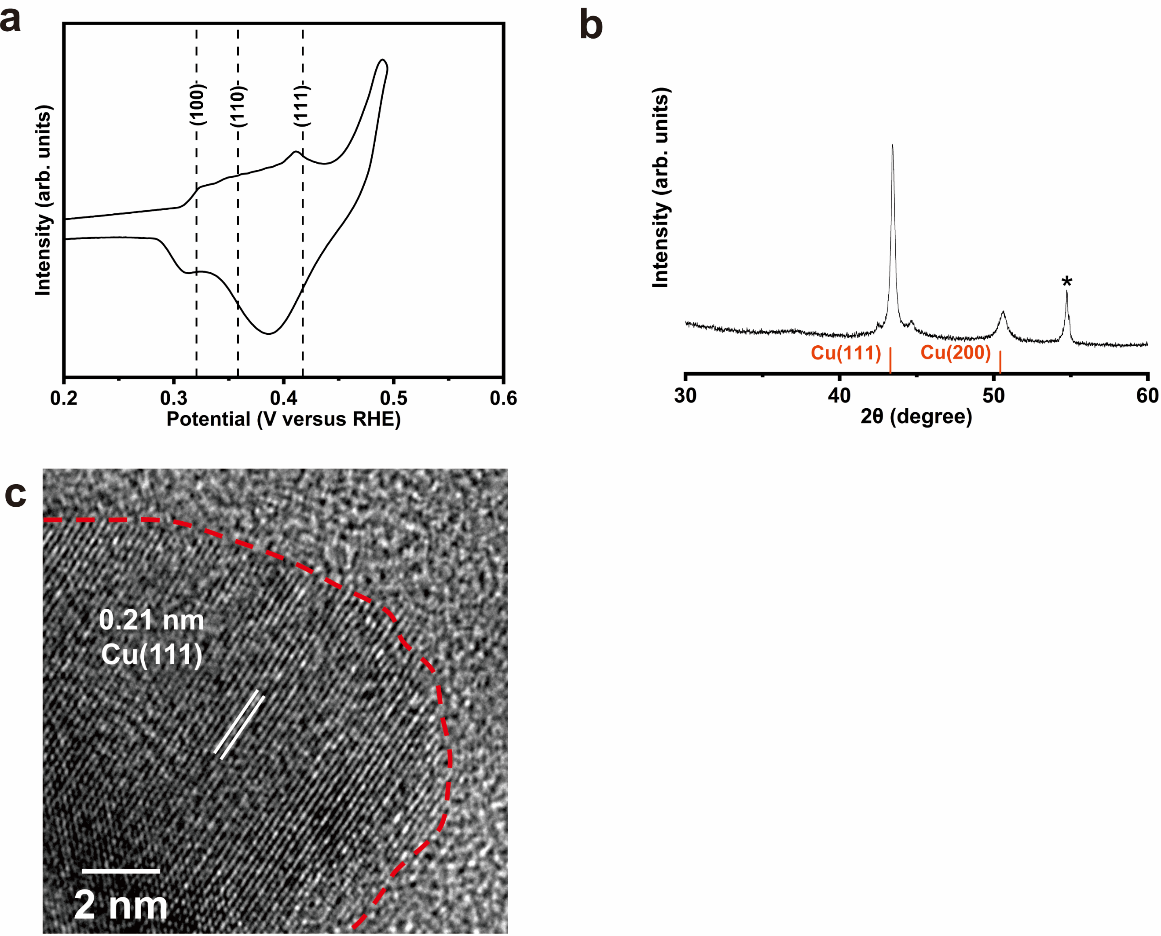
**

**Supplementary Fig. 22.** **Structural characterization of LRS-Cu after CO_2_ reduction. a** CV, **b** XRD pattern and **c** TEM image of LRS-Cu after CO_2_ reduction reaction.

The red dash line indicates the rough surface. The peak marked with an asterisk in the XRD pattern originates from the substrate.


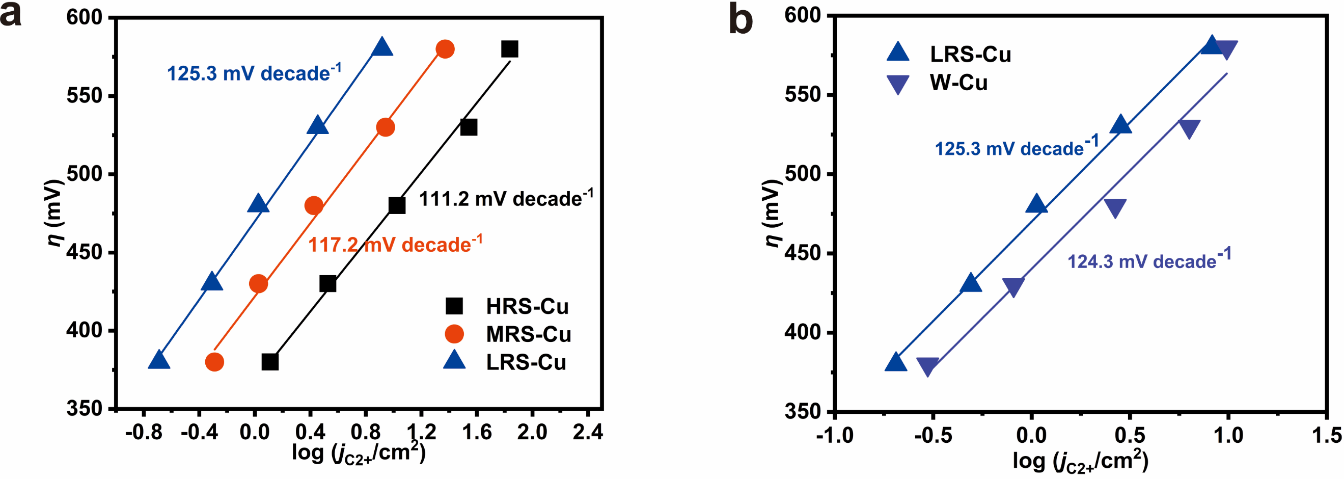


**Supplementary Fig. 23.** **Tafel analysis of different samples.**


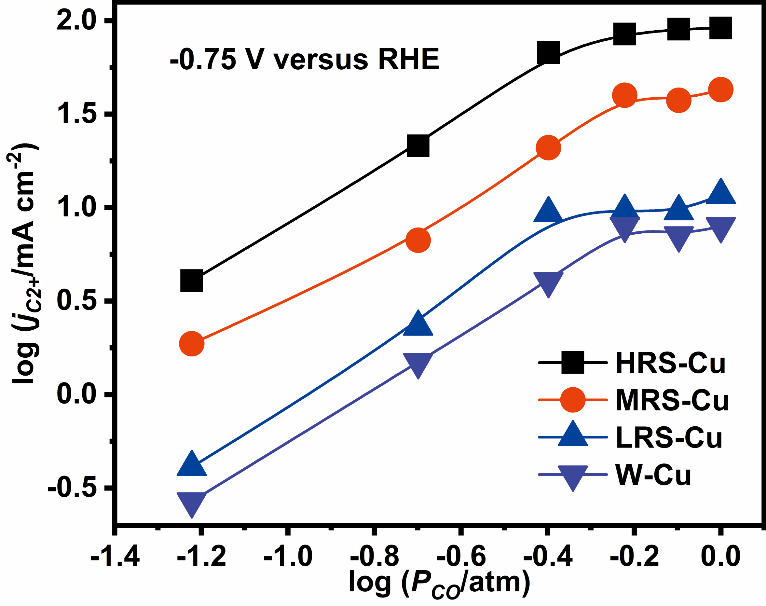


**Supplementary Fig. 24.** **Partial current densities of C2+ products on different samples as a function of CO partial pressure (CO / Ar mixture).**

**
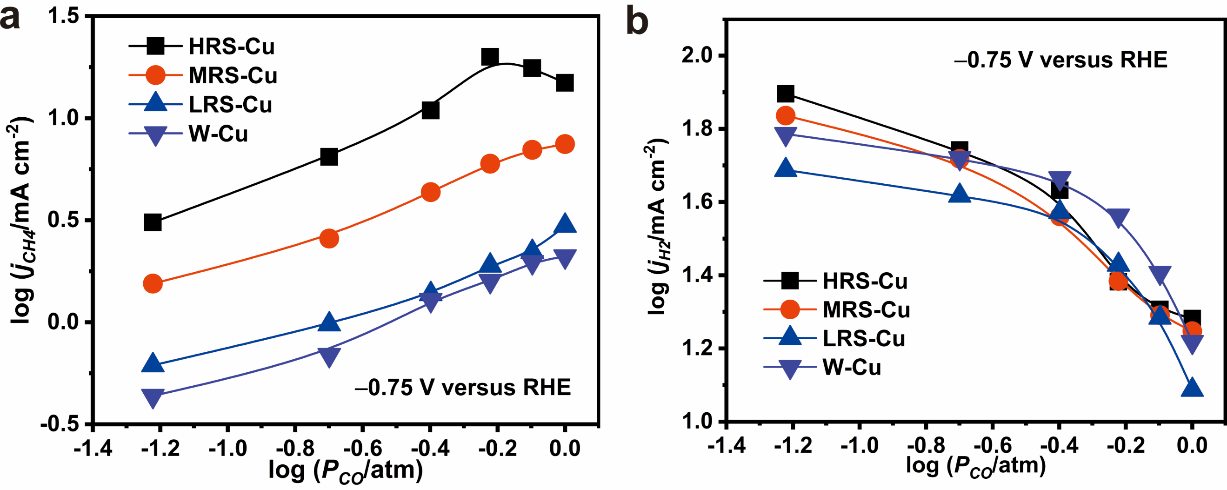
**

**Supplementary Fig. 25.** **Partial current densities of different products at different partial pressures of CO.** Partial current densities of **a** methane and **b** hydrogen on different samples as a function of CO partial pressure (CO / Ar mixture).


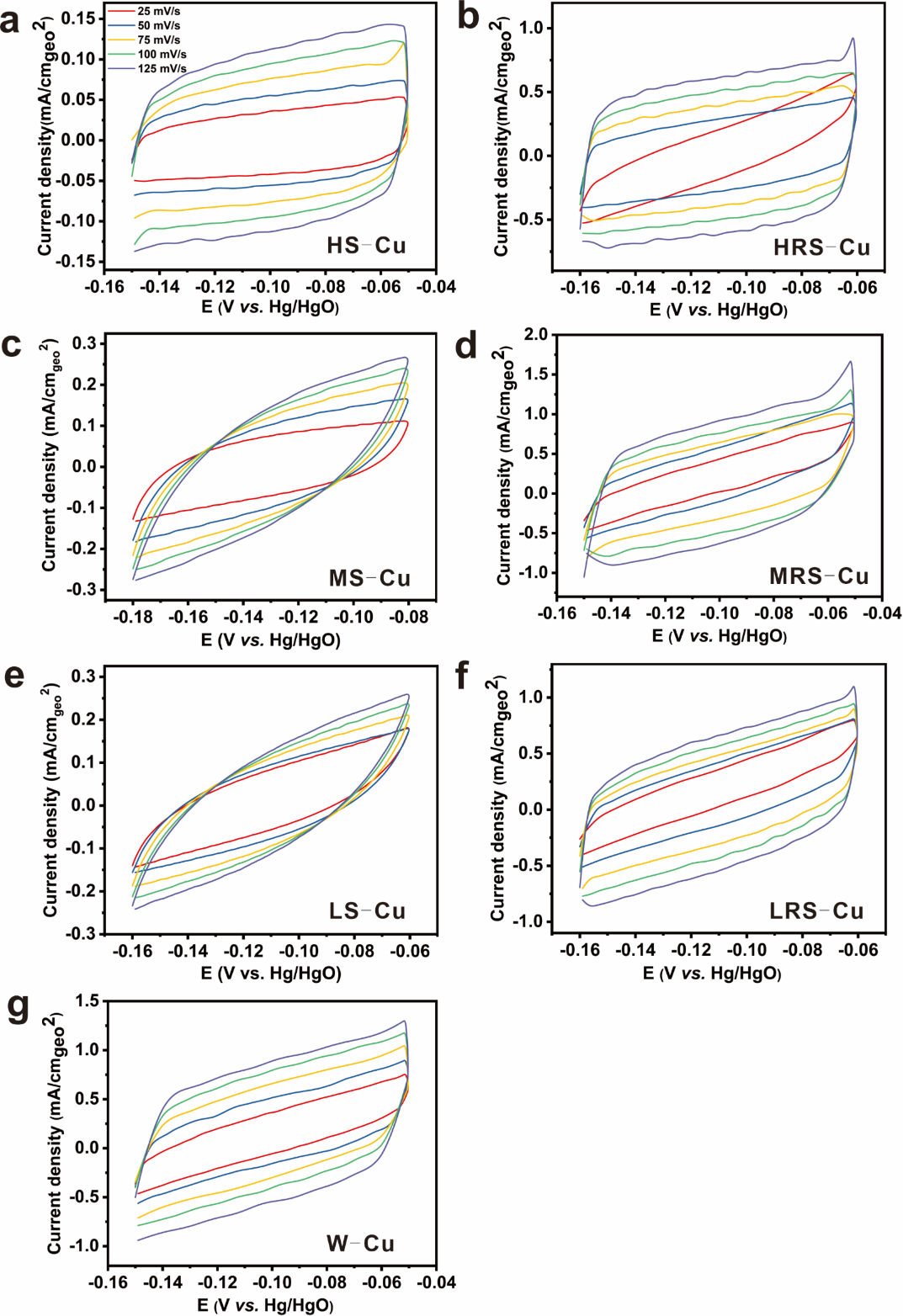


**Supplementary Fig. 26.** **The non-Faraday CV profiles obtained on different samples.** **a** HS-Cu, **b** HRS-Cu, **c** MS-Cu, **d** MRS-Cu, **e** LS-Cu, **f** LRS-Cu and **g** W-Cu at different sweeping rates (25, 50, 75, 100, and 125 mV/s).


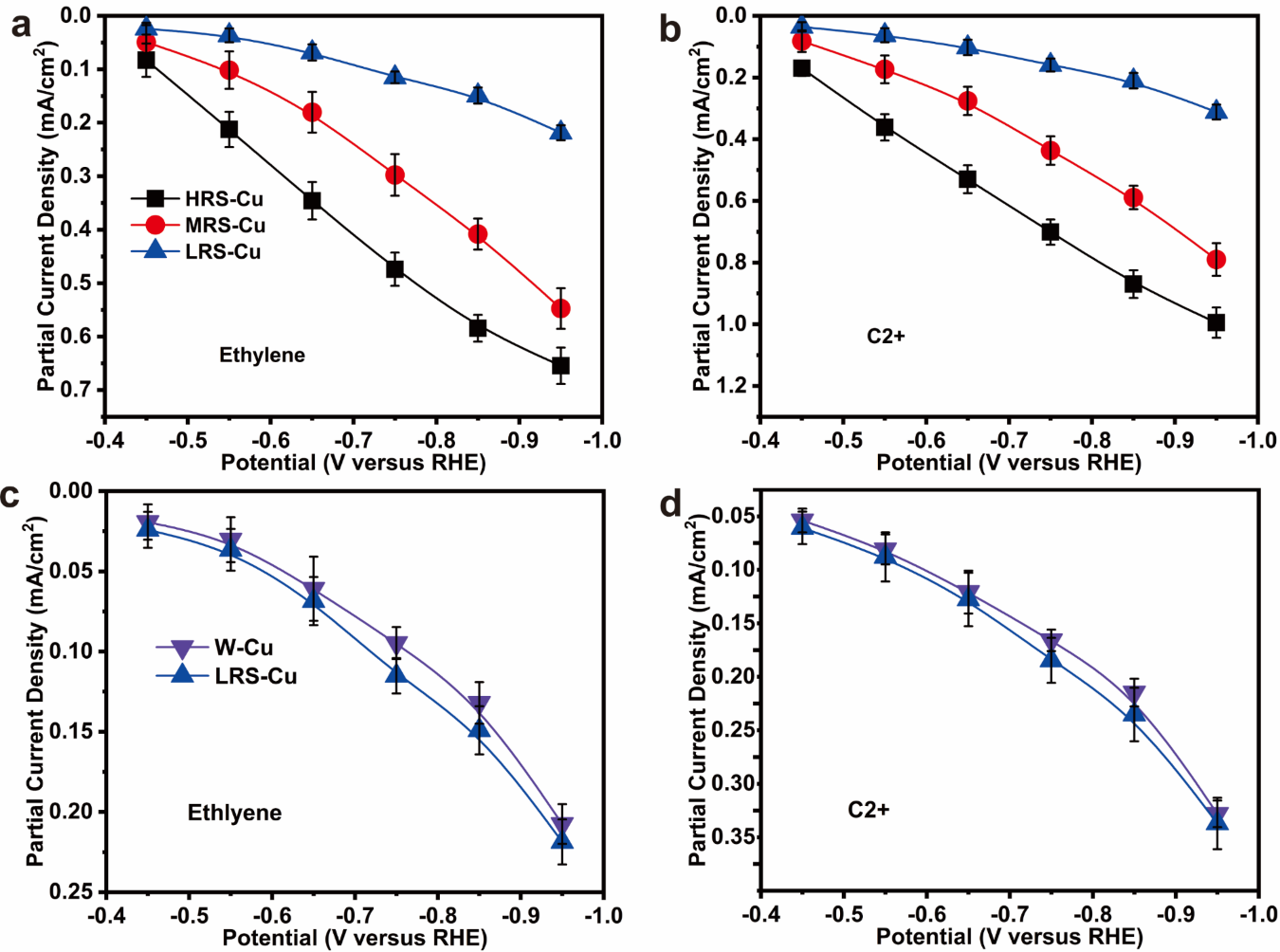


**Supplementary Fig. 27.** **ECSA-corrected ethylene and C2+ products partial current density on different samples.**

Error bars represent the standard deviation from at least three independent measurements.


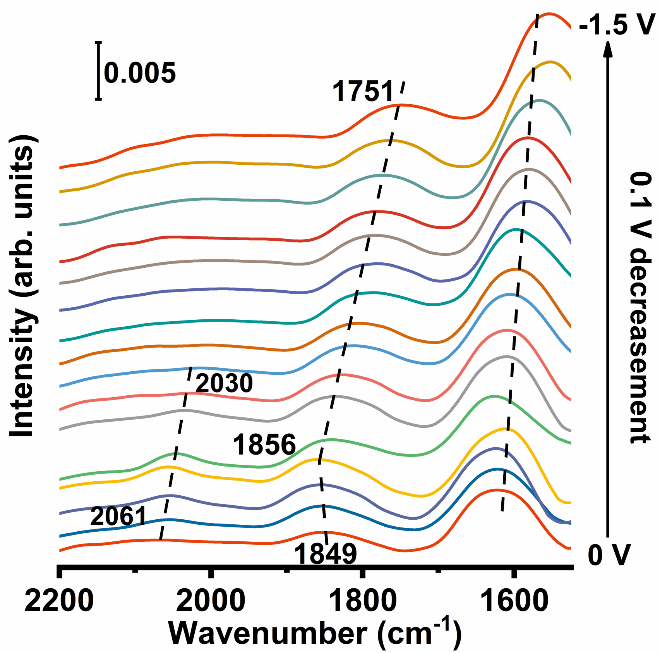


**Supplementary Fig. 28.** **In-situ ATR-SEIRAS spectra of the control sample.**


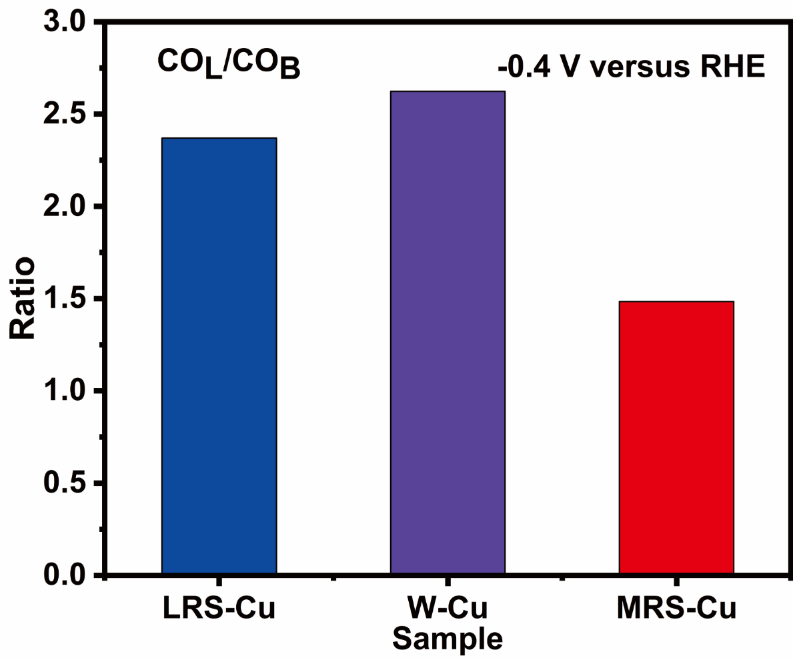


**Supplementary Fig. 29.** **CO_B_/CO_L_ peak area ratio of different samples.**

**
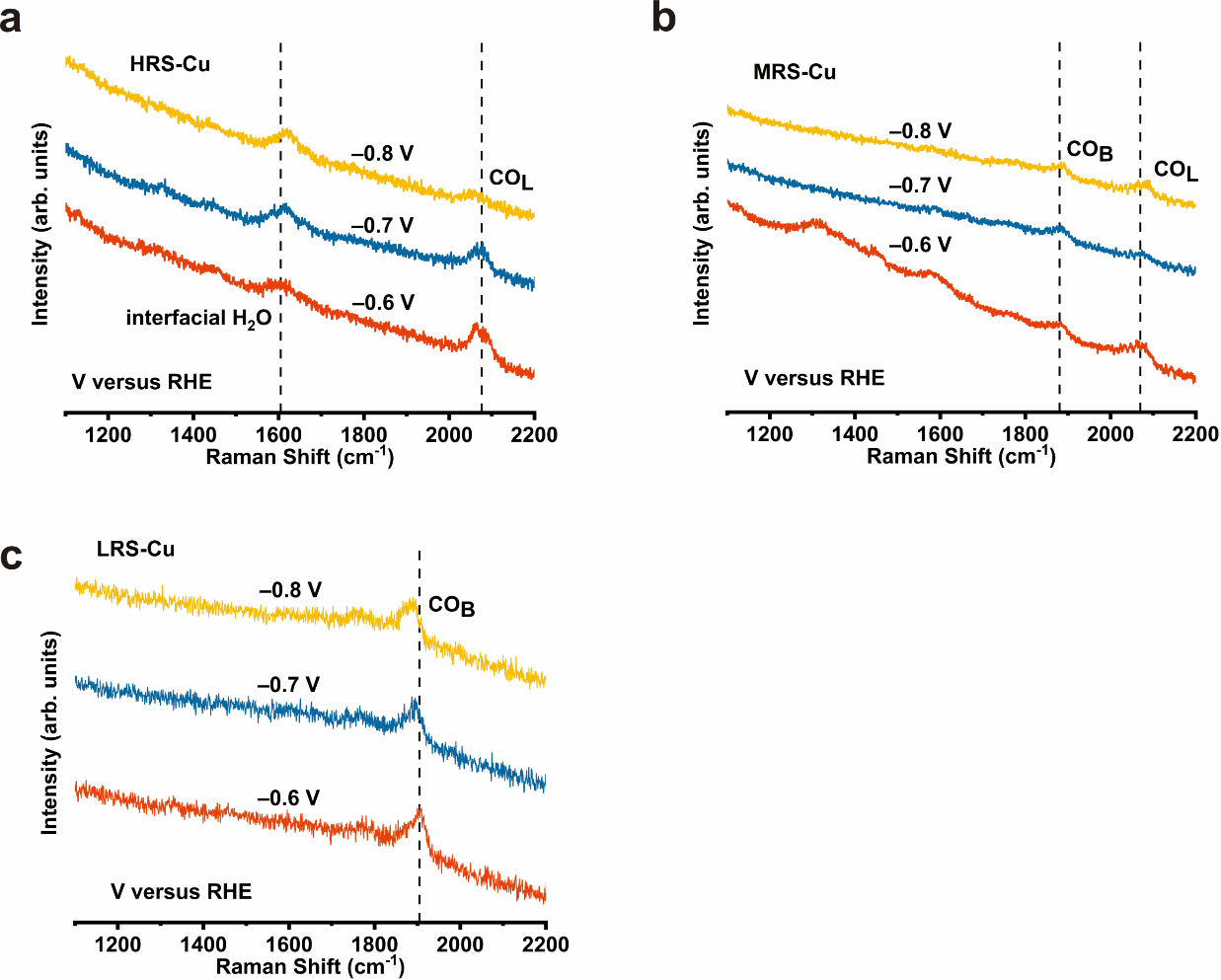
**

**Supplementary Fig. 30.** **In-situ Raman spectra of different samples.**

Unlike the testing method for the results shown in Supplementary Fig. 12, a 785 nm laser was used to obtain the results shown in this figure to avoid fluorescence. In this work, the peak intensity of the *CO detected by Raman is lower compared to the peak intensity of the *CO detected by IR. The use of a more advanced cell design is expected to enhance the signal intensity of Raman.^20^


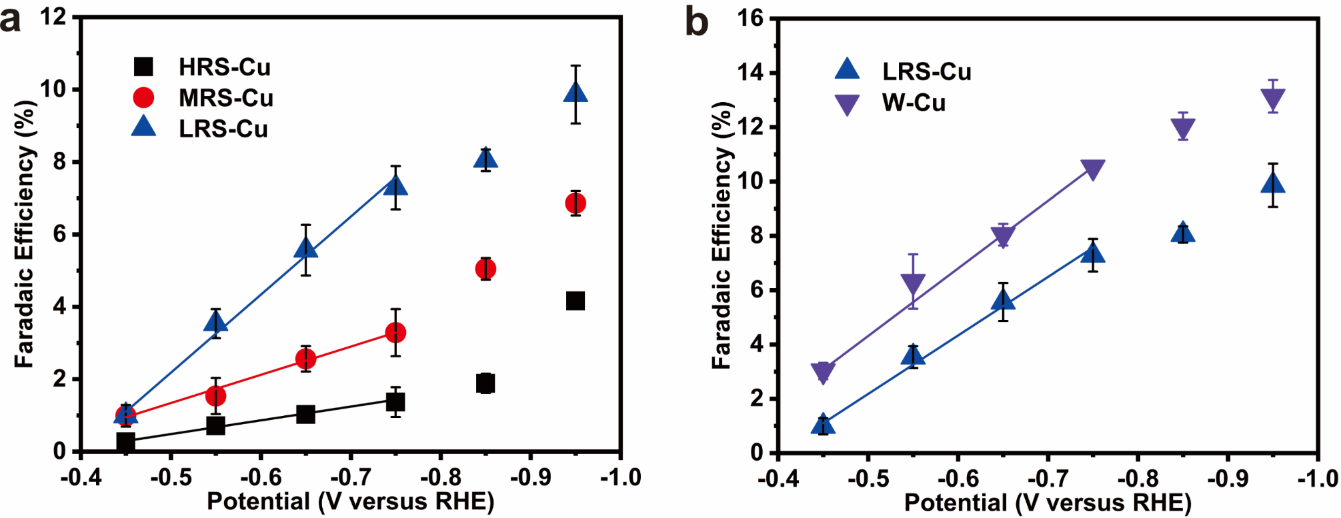


**Supplementary Fig. 31.** **Comparisons of Faradaic efficiency of methane produced by different samples under a range of different potentials.**

The fitted straight line is for easy comparison of the change rate of methane produced by different samples. Error bars represent the standard deviation from at least three independent measurements.


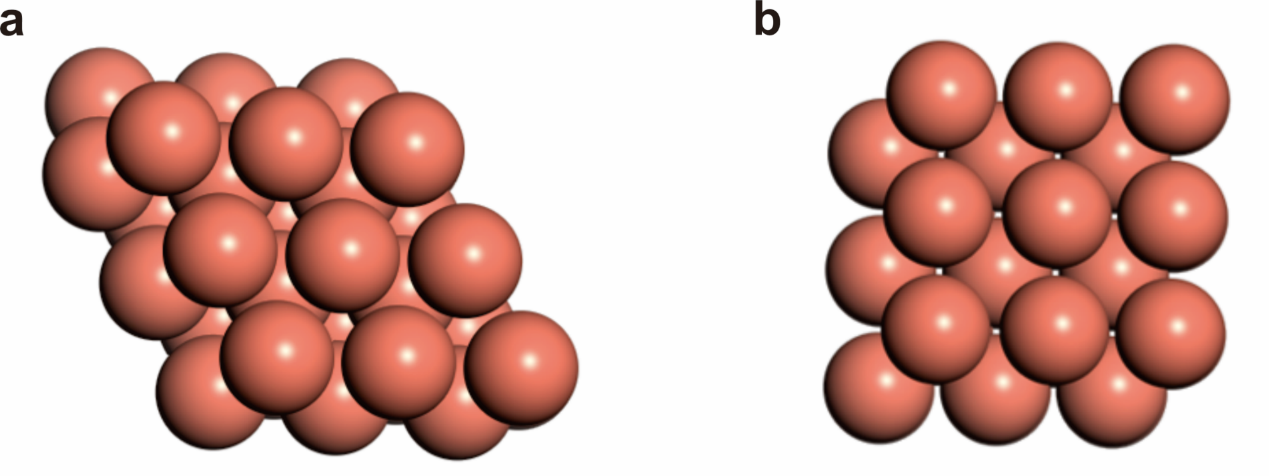


**Supplementary Fig. 32. Models for DFT calculations.** **a** Cu(111) and **b** Cu(100).

**
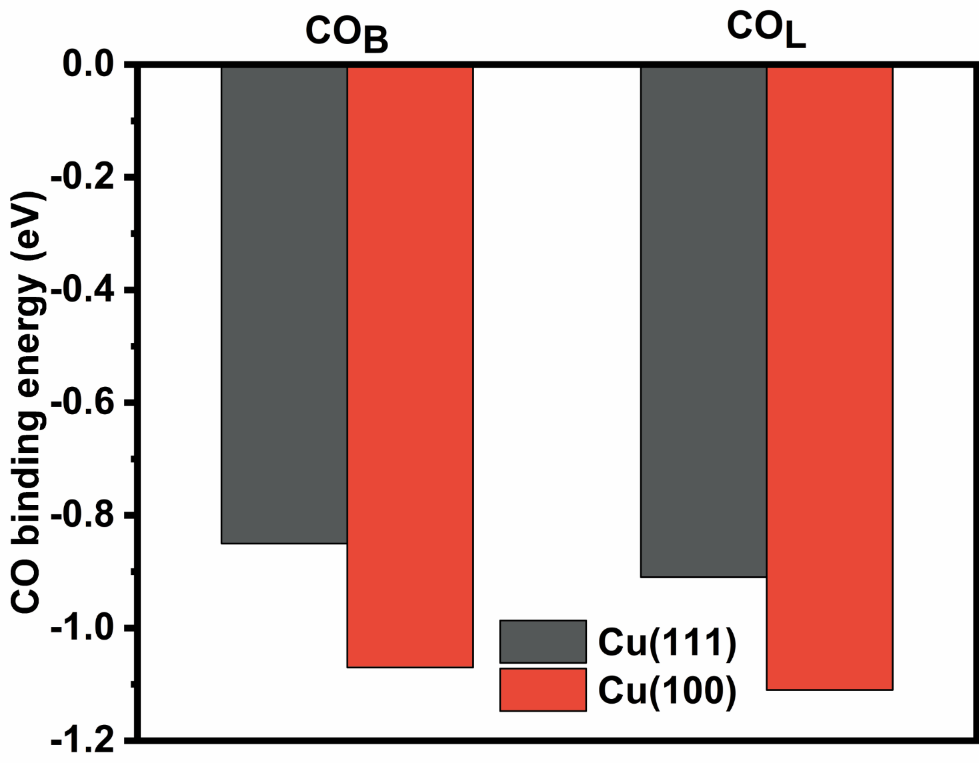
**

**Supplementary Fig. 33.** **The binding energy of CO on different Cu facets.**


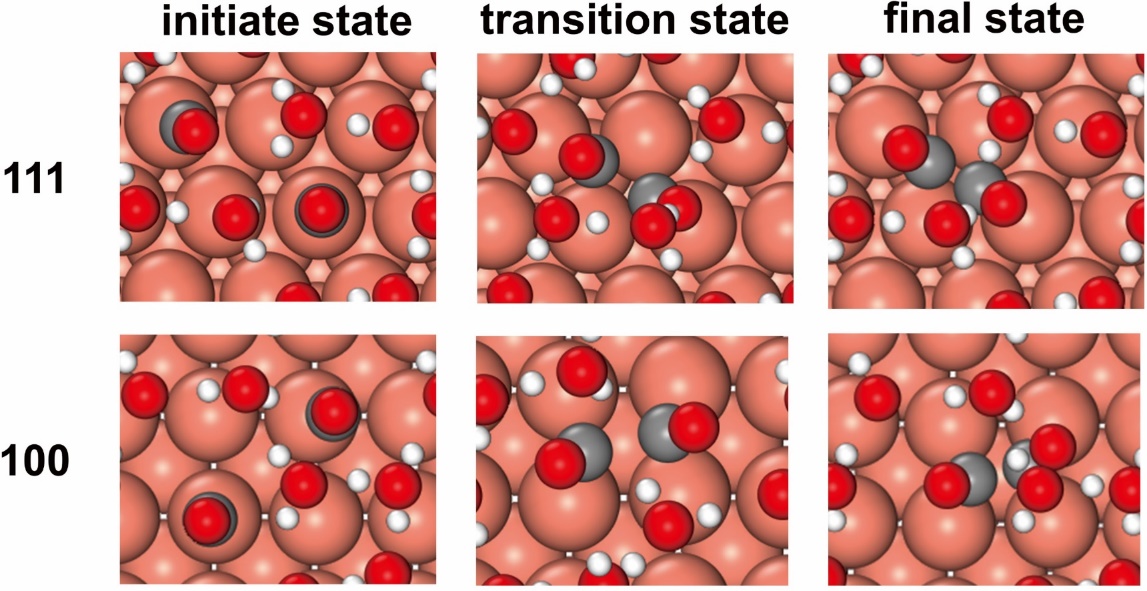


**Supplementary Fig. 34.** **Top views of the geometries of CO_L_-CO_L_dimerization on the Cu(111) and Cu(100) facet.**

Red, grey and orange balls stand for O, C and Cu atoms, respectively. Water molecules are also included.


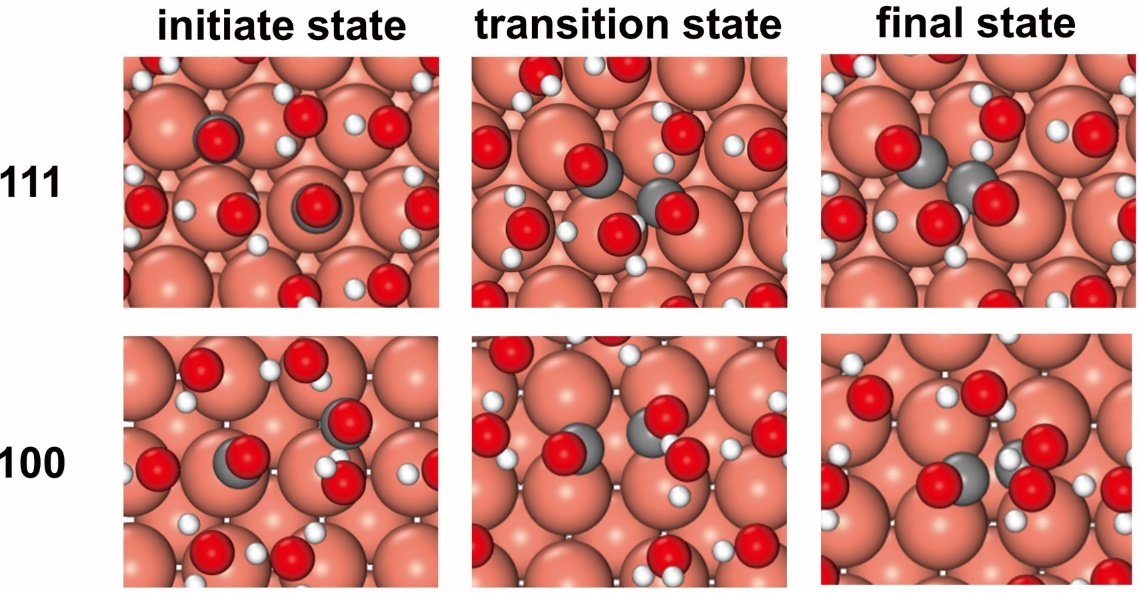


**Supplementary Fig. 35.** **Top views of the geometries of CO_B_-CO_L_ dimerization on the Cu(111) and Cu(100) facet.**

Red, grey and orange balls stand for O, C and Cu atoms, respectively. Water molecules are also included.


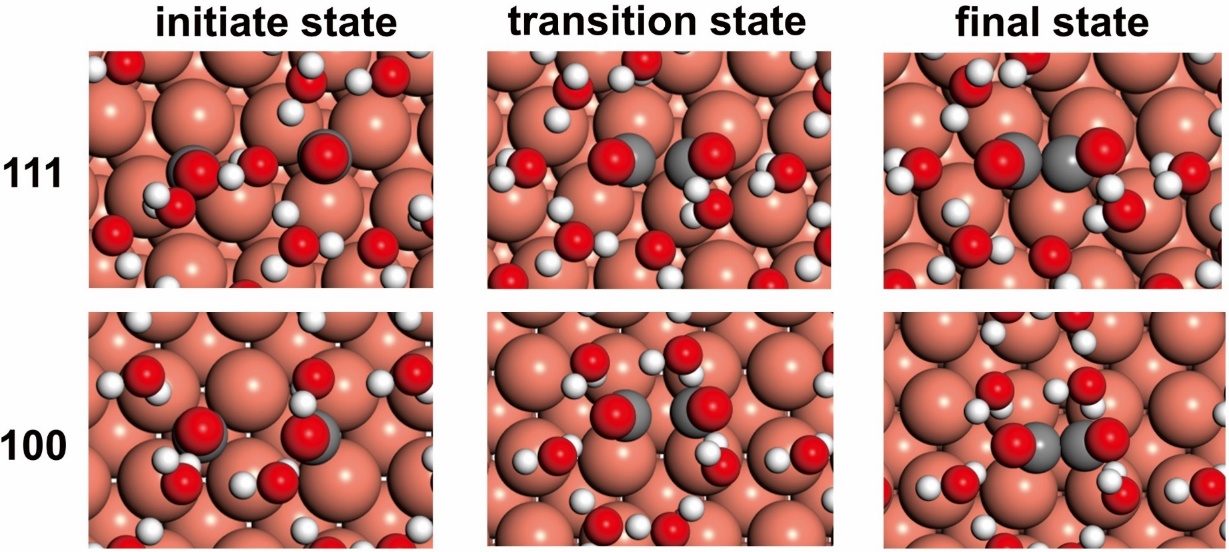


**Supplementary Fig. 36.** **Top views of the geometries of CO_B_-CO_L_ dimerization on the Cu(111) and Cu(100) facet.**

Red, grey and orange balls stand for O, C and Cu atoms, respectively. Water molecules are also included.


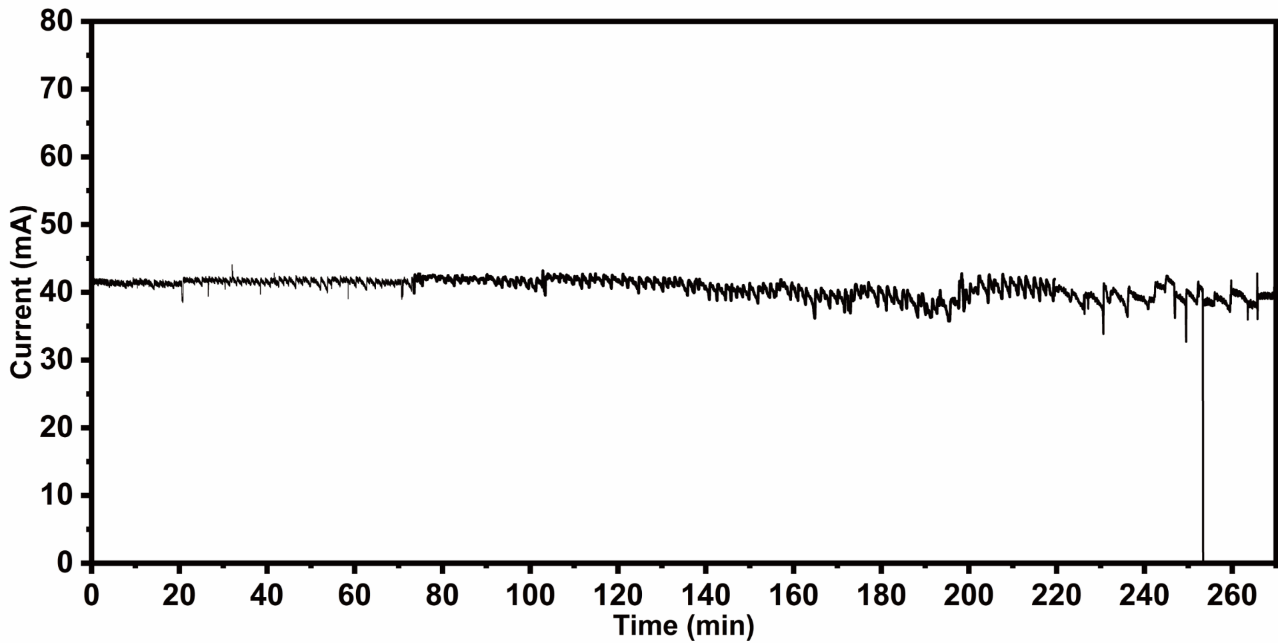


**Supplementary Fig. 37.** **Cathode current of the HRS-Cu in the PV-EC system during the entire stability test.**


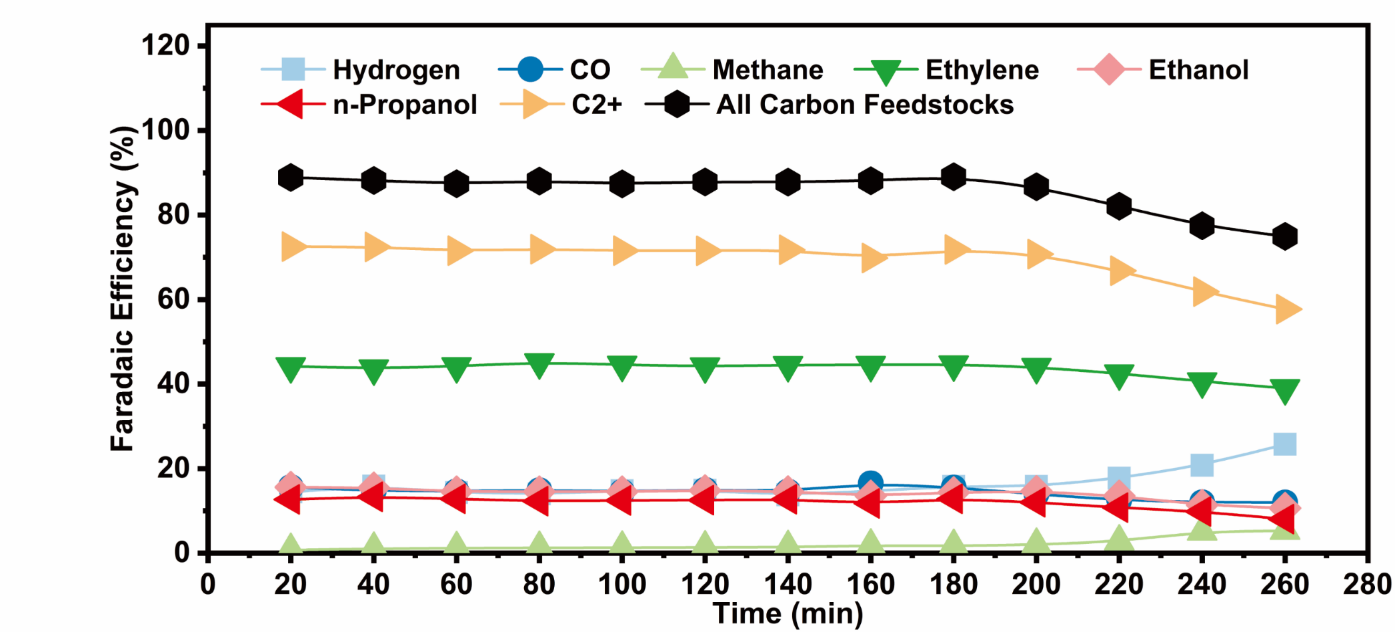


**Supplementary Fig. 38.** **Faradaic efficiencies of all CO_2_ reduction products on the HRS-Cu in the PV-EC system during the stability test.**

Carbon feedstocks include CO, methane, ethylene, ethanol, and n-propanol. C2+ products include ethylene, ethanol and n-propanol. This figure exhibits the performance during the entire stability test, where the stable part of the test is shown as Fig. 4. The gradual decrease in selectivity is mainly due to the loss in the hydrophobicity of the GDL and the formation of carbonate. Excessive water and formed carbonate block the channels in the GDL, which hinders CO_2_ mass transfer and reduces the selectivity of CO_2_ reduction.


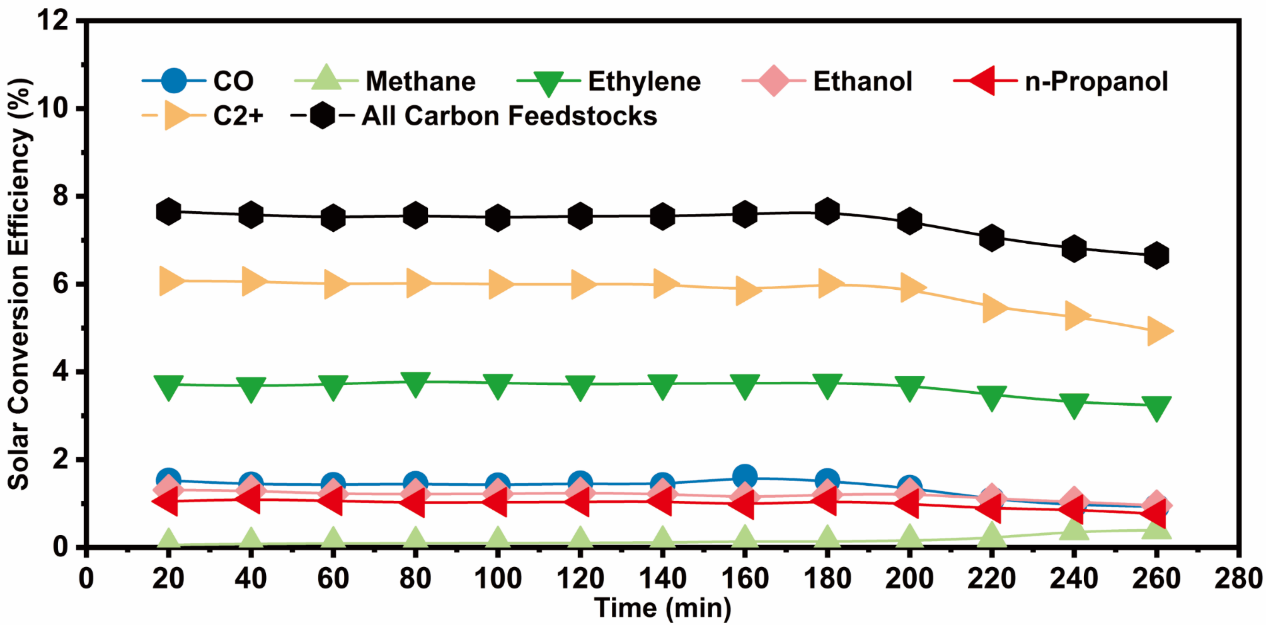


**Supplementary Fig. 39.** **The solar conversion efficiency of all carbon feedstocks on the HRS-Cu in the PV-EC system during the stability test.**

Carbon feedstocks include CO, methane, ethylene, ethanol, and n-propanol. C2+ products include ethylene, ethanol and n-propanol. This figure exhibits the performance during the entire stability test, where the stable part of the test is shown as Fig. 4. The gradual decrease in solar conversion efficiency is mainly due to the loss in the hydrophobicity of the GDL and the formation of carbonate. Excessive water and formed carbonate block the channels in the GDL, which hinders CO_2_ mass transfer and reduces the solar conversion efficiency of CO_2_ reduction.


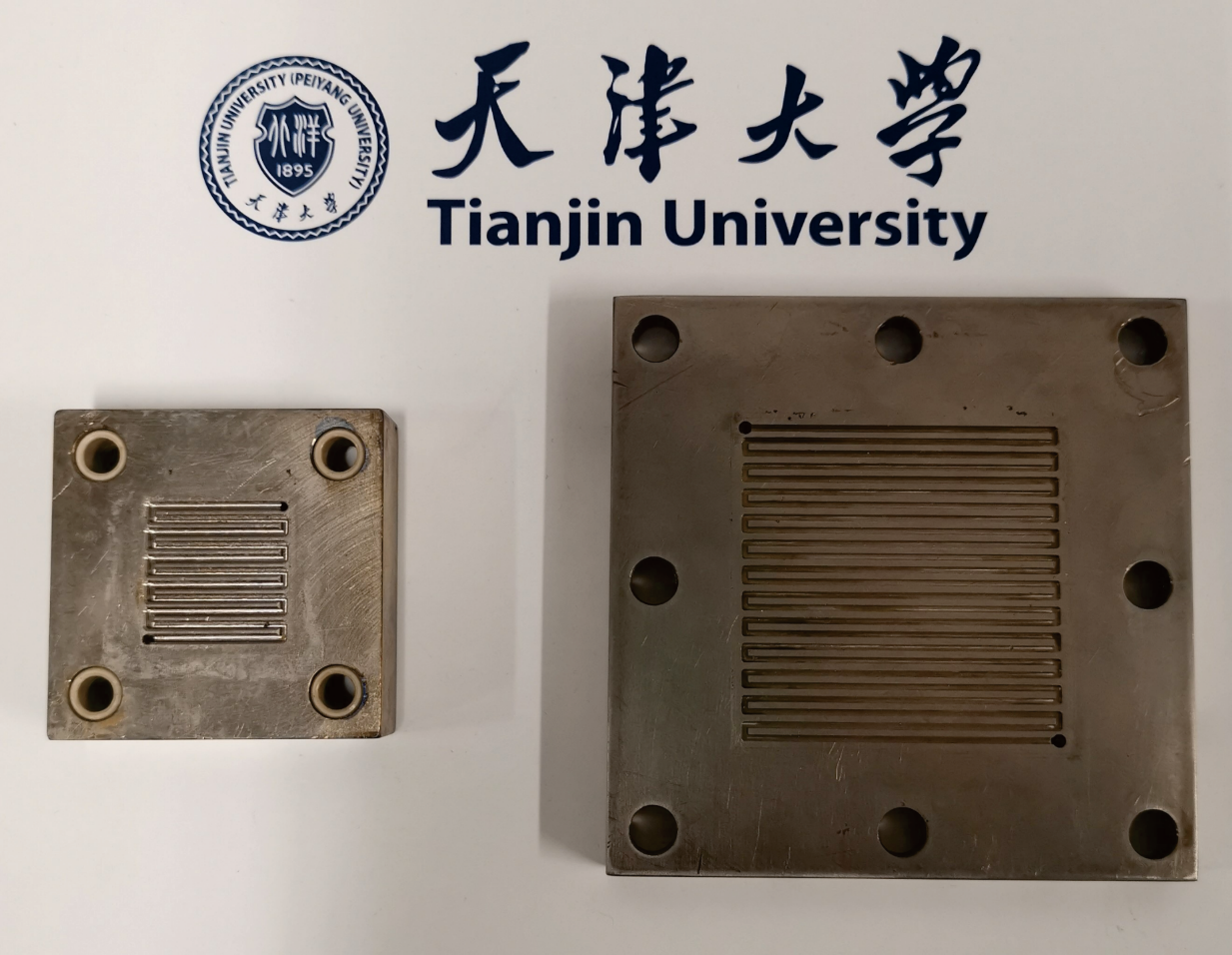


**Supplementary Fig. 40.** **Photographs of bipolar plates with different flow field areas.**

The area of the flow field on the left is 4 cm^2^ (i.e., 4 cm^2^-MEA), and the flow field area on the right is 25 cm^2^ (i.e., 25 cm^2^-MEA).


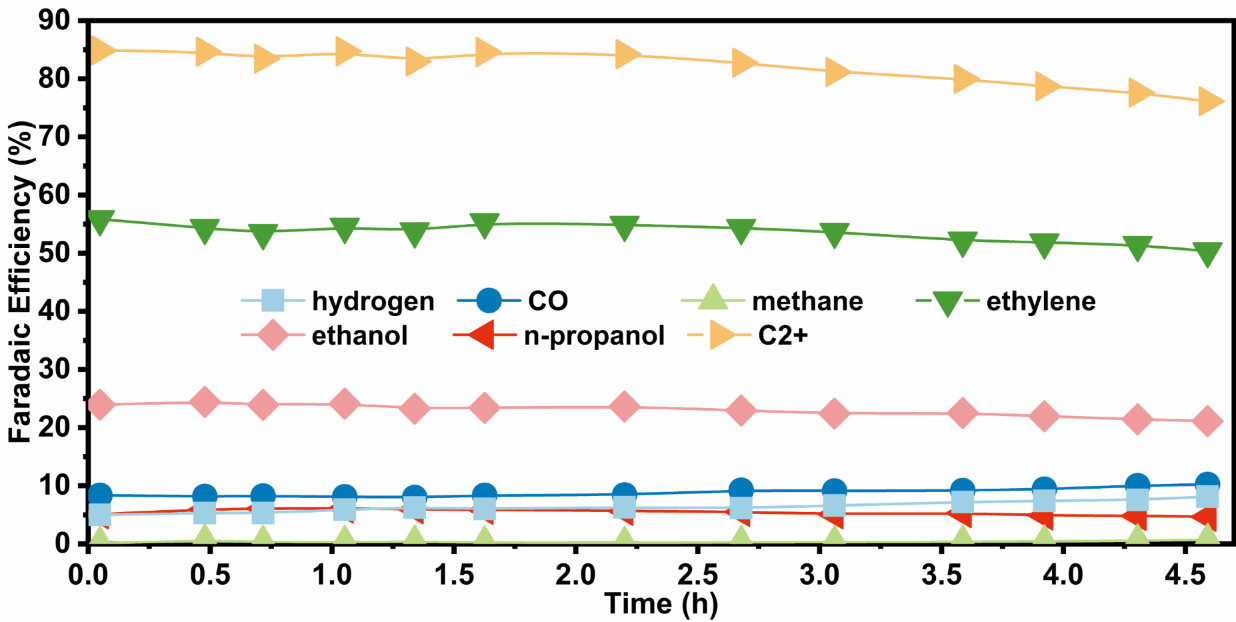


**Supplementary Fig. 41.** **F****aradaic efficiencies of all CO_2_ reduction products on the HRS-Cu in the 4 cm^2^-MEA system during the stability test.**


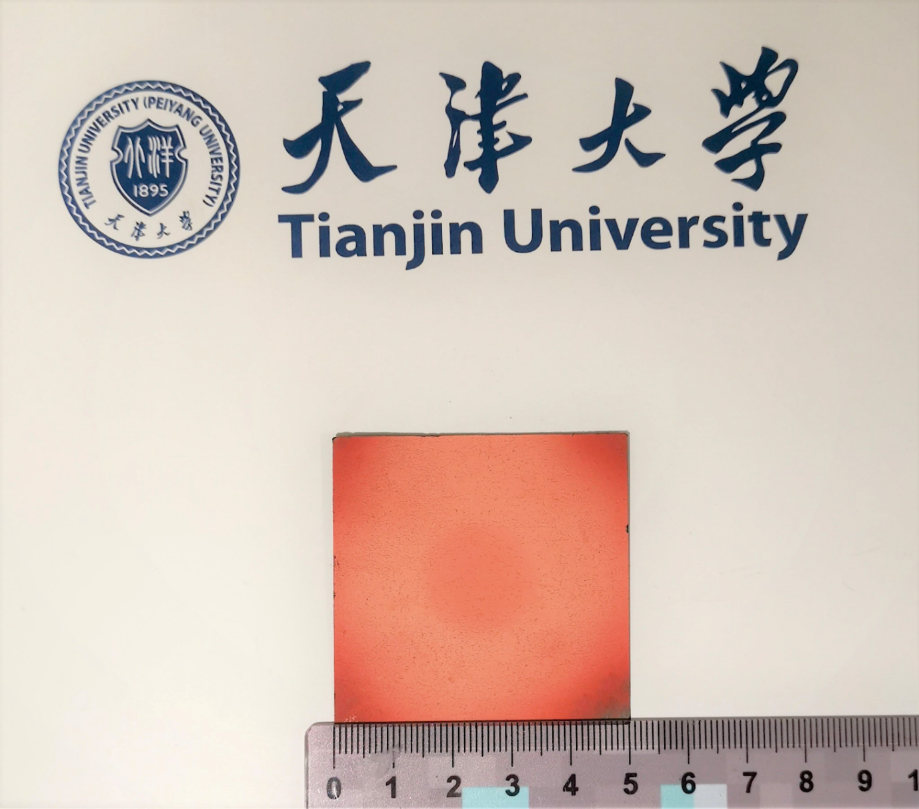


**Supplementary Fig. 42.** **A typical photograph of the HRS-Cu electrode with a 25 cm^2^ active area.**


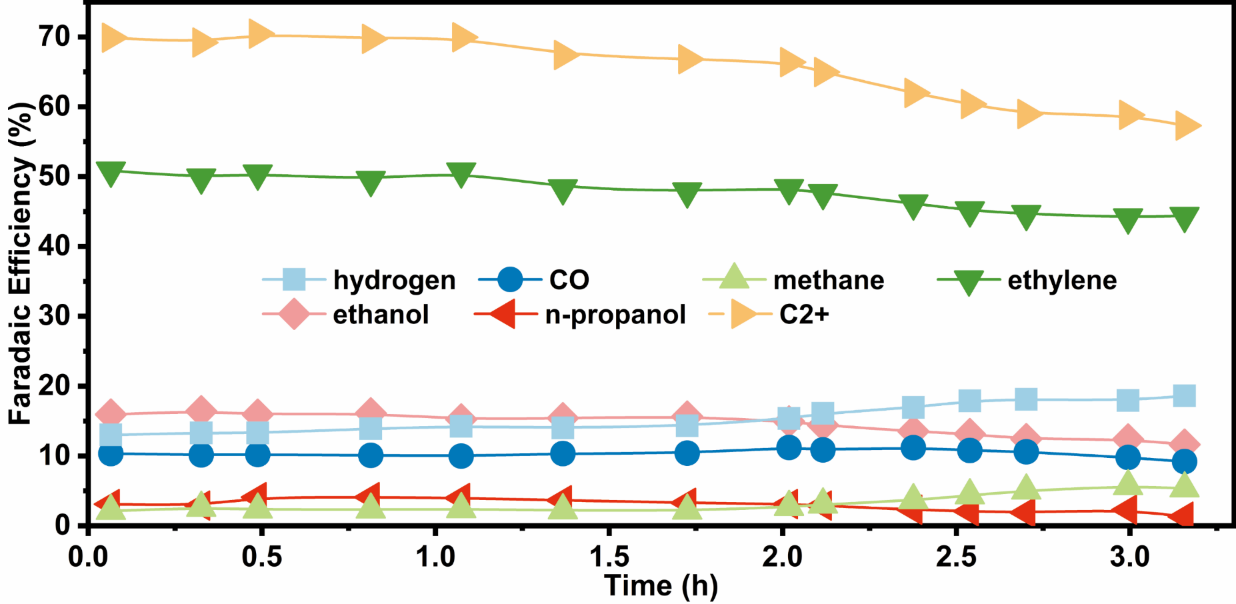


**Supplementary Fig. 43.** **Faradaic efficiencies of all CO_2_ reduction products on the HRS-Cu in the 25 cm^2^-MEA system during the stability test.**

**Supplementary Table 1.**

**Double-layer capacitance and roughness factors of different samples**

| Sample | C_dl_  mF/cm^2^ | SRF |
| --- | --- | --- |
| HRS-Cu | 4.25 | 146.6 |
| HS-Cu | 0.739 | 25.50 |
| MRS-Cu | 4.51 | 155.5 |
| MS-Cu | 0.692 | 23.86 |
| LRS-Cu | 4.30 | 148.3 |
| LS-Cu | 0.594 | 20.48 |
| W-Cu | 5.22 | 180.0 |
| Poly Cu | 0.029^5^ | / |

**Supplementary Table 2.**

**The amount of Cu loaded on different samples before CO_2_ reduction reaction**

| Sample | mass loading/ (mg cm^-2^) |
| --- | --- |
| HRS-Cu | 0.832 |
| MRS-Cu | 0.853 |
| LRS-Cu | 0.891 |
| W-Cu | 0.912 |

**Supplementary Table 3.**

**The amount of Cu loaded on different samples after CO_2_ reduction reaction**

| Sample | mass loading/ (mg cm^-2^) |
| --- | --- |
| HRS-Cu | 0.833 |
| MRS-Cu | 0.855 |
| LRS-Cu | 0.893 |
| W-Cu | 0.863 |

The weight of the HRS-Cu, MRS-Cu and LRS-Cu electrodes increased slightly after the CO_2_ reduction reaction, and the increased weight could be attributed to the carbonate salt blocked in the GDL channels. However, due to the peeling-off effect, the weight of the W-Cu electrode is significantly reduced (reduced by 0.049 mg), indicating that the contact between the supported catalysts and the substrate in the traditional electrode assembly process is weak, which is not beneficial to improving the E.C.E. and stability of the reaction system.

**Supplementary Table 4.** **Summary of optimal F.E., full-cell E.C.E. and C.E. towards C2+ products from different systems ***

| No. | Cathode | Area | Preparation method | F.E. ** | E.C.E. **  at maximum F.E. | Yields **  at maximum F.E. | Ref. |
| --- | --- | --- | --- | --- | --- | --- | --- |
| 1 | Molecular modified Cu film | 1 cm^2^ | Electrodeposition of molecular film on Cu film | 84.1% (71.5%) | 25.1% (21.4%) | 1.96% (1.92%) | *Nature* 2020^6^ |
| 2 | Cu-Al alloy | 1 cm^2^ | Co-sputtering and chemical etching process | 90% (80%) | 26.2% (21.9%) | 1.08% (1.06%) | *Nature* 2020^7^ |
| 3 | polyamine-incorporated Cu | 1 cm^2^ | Electrodeposition of Cu and polyamine on carbon-based GDL | 93.0% (87.0%) | 56.9% (49.5%) | 0.77% (0.75%) | *Nat. Catal.* 2021^8^ |
| 4 | Highly porous OD-Cu particles | 1 cm^2^ | Annealing Cu powder and drop-casting | 76.8% (42.0%) | 23.8% (13.0%) | 4.11% (2.84%) | *Nat. Catal.* 2018^9^ |
| 5 | Cu nanocavities | 0.5 cm^2^ | Colloidal synthesis and drop-casting | 58.8% (38.0%) | 27.0% (17.3%) | 0.04% (0.03%) | *Nat. Catal.* 2018^10^ |
| 6 | Branched CuO particles | 0.5 cm^2^ | Colloidal synthesis and drop-casting | 68.0% (68.0%) | 21.7% (21.7%) | 0.02% (0.02%) | *J. Am. Chem. Soc.* 2019^11^ |
| 7 | Fragmented Cu nanoparticles | 0.5 cm^2^ | Colloidal synthesis and drop-casting | 71.8% (57.3%) | 22.9% (18.3%) | 0.04% (0.04%) | *J. Am. Chem. Soc.* 2019^12^ |
| 8 | Multi-hollow Cu_2_O | 1 cm^2^ | Colloidal synthesis and drop-casting | 70.4% (38.0%) | 26.9% (14.6%) | 0.75% (0.49%) | *J. Am. Chem. Soc* 2020^13^ |
| 9 | Cu(100)-rich film | 0.64 cm^2^ | High energy deposition-etch-bombardment | 86.6% (58.6%) | 36.5% (24.8%) | 0.69% (0.61%) | This work |
| 10 | Cu(100)-rich film | 4 cm^2^ | High energy deposition-etch-bombardment | 84.9% (55.9%) | 40.2% (26.4%) | 2.07% (1.74%) | This work |
| 11 | 25 cm^2^-Cu(100)-rich film | 25 cm^2^ | High energy deposition-etch-bombardment | 69.9% (50.9%) | 14.9% (10.9%) | 13.2% (12.0%) | This work |

* Only the results of studies using single metallic Cu-based catalysts are included.

** The value in parentheses is the F.E., E.C.E and C.E. of ethylene.

**Supplementary Table 5. Summary of different PV-EC systems ***

| No. | S.T.C. ** | F.E. | Stability | Cathode | Anode | Effective illuminated area | Light-harvesting materials | Ref. |
| --- | --- | --- | --- | --- | --- | --- | --- | --- |
| 1 | 1.6% | 34.0% (34.0%) *** | 21600 s | Dendritic nanostructured CuO | The same as cathode | 1.5 cm^2^ | FA_1−_*_x_*MA*_x_*Pb(I_1−_*_y_*Br*_y_*)_3_  perovskite | *Proc. Natl. Acad. Sci. USA* 2019^15^ |
| 2 | 2.4% (AM 1.0) | 46.7% (31.9%) | 7200 s | Cu_2_O derived Cu | IrO*_x_* | 1.9 cm^2^ | p-n^+^ Si | *ACS Sustain. Chem. Eng.* 2017^16^ |
| 3 | 3.0% | 39.0% (22.0) | Not reported | Nanocoral CuAg bimetal | IrO_2_ nanotube | 2.0 cm^2^ | III-V/Si tandem cells  with MPP trackers | *Energy Environ. Sci.* 2017^14^ |
| 4 | 1.0% | 30.0% (20.0%) | 6300 s | Ag-supported dendritic Cu | IrO_2_ nanotube | 1.0 cm^2^ | CH_3_NH_3_PBI_3_ perovskite | *Energy Environ. Sci.* 2019^17^ |
| 5 | 5.4% | 71.1% (54.3%) | 3600 s | Ag decorated Cu nanowires | IrO*_x_* | 0.75 cm^2^ | Cs*_x_*(MA_0.17_FA_0.83_)_(100−_*_x_*_)_Pb(I_0.83_Br_0.17_)_3_ perovskite | *J. Am. Chem. Soc.* 2019^18^ |
| 6 | ~6.0% | 71.6% (44.4%) | 12000 s | Cu(100)-rich film | Ni foam | 5.7 cm^2^ | p-n^+^ Si | This work |

* Only the results of studies using Cu-based catalysts are included.

** The S.T.C. and F.E. calculations include only C2+ products.

*** The value in parentheses is the F.E. of ethylene.

**Supplementary Table 6.**

**CO_2_ reduction reactions and electrochemical data for carbonaceous products^14^**

| Carbon feedstock | Overall reaction | *E^0^* |
| --- | --- | --- |
| CO | CO_2_ ↔ CO +1/2O_2_ | 1.334 |
| methane | CO_2_ + 2H_2_O ↔ CH_4_ + 2O_2_ | 1.059 |
| ethylene | 2CO_2_ + 2H_2_O ↔ C_2_H_4_ + 3O_2_ | 1.150 |
| ethanol | 2CO_2_ + 3H_2_O ↔ C_2_H_5_OH + 3O_2_ | 1.147 |
| n-propanol | 3CO_2_ + 4H_2_O ↔ C_3_H_8_O + 9/2O_2_ | 1.131 |

**Supplementary References**

1. Rosen J, et al. Mechanistic Insights into the Electrochemical Reduction of CO_2_ to CO on Nanostructured Ag Surfaces. *ACS Catal.* **5**, 4293-4299 (2015).

2. Gao S, et al. Atomic Layer Confined Vacancies for Atomic-level Insights into Carbon Dioxide Electroreduction. *Nat. Commun.* **8**, 14503 (2017).

3. Goodpaster JD, Bell AT, Head-Gordon M. Identification of Possible Pathways for C-C Bond Formation during Electrochemical Reduction of CO_2_: New Theoretical Insights from an Improved Electrochemical Model. *J. Phys. Chem. Lett.* **7**, 1471-1477 (2016).

4. Droog JMM, Schlenter B. Oxygen Electrosorption on Copper Single Crystal Electrodes in Sodium Hydroxide Solution. *J.Electroanal. Chem. Interfacial Electrochem.* **112**, 387-390 (1980).

5. Li CW, Kanan MW. CO_2_ Reduction at Low Overpotential on Cu Electrodes Resulting from the Reduction of Thick Cu_2_O Films. *J. Am. Chem. Soc.* **134**, 7231-7234 (2012).

6. Li F, et al. Molecular Tuning of CO_2_-to-ethylene Conversion. *Nature* **577**, 509-513 (2020).

7. Zhong M, et al. Accelerated Discovery of CO_2_ Electrocatalysts Using Active Machine Learning. *Nature* **581**, 178-183 (2020).

8. Chen XY, et al. Electrochemical CO_2_-to-ethylene Conversion on Polyamine-incorporated Cu Electrodes. *Nat. Catal.* **4**, 20-27 (2021).

9. Jouny M, Luc W, Jiao F. High-rate Electroreduction of Carbon Monoxide to Multi-carbon Products. *Nat. Catal.* **1**, 748-755 (2018).

10. Zhuang TT, et al. Steering Post-C-C Coupling Selectivity Enables High Efficiency Electroreduction of Carbon Dioxide to Multi-carbon Alcohols. *Nat. Catal.* **1**, 421-428 (2018).

11. Kim J, Choi W, Park JW, Kim C, Kim M, Song H. Branched Copper Oxide Nanoparticles Induce Highly Selective Ethylene Production by Electrochemical Carbon Dioxide Reduction. *J. Am. Chem. Soc.* **141**, 6986-6994 (2019).

12. Jung H, et al. Electrochemical Fragmentation of Cu_2_O Nanoparticles Enhancing Selective C-C Coupling From CO_2_ Reduction Reaction. *J. Am. Chem. Soc.* **141**, 4624-4633 (2019).

13. Yang PP, et al. Protecting Copper Oxidation State via Intermediate Confinement for Selective CO_2_ Electroreduction to C2+ Fuels. *J. Am. Chem. Soc.* **142**, 6400-6408 (2020).

14. Gurudayal, et al. Efficient Solar-driven Electrochemical CO_2_ Reduction to Hydrocarbons and Oxygenates. *Energy Environ. Sci.* **10**, 2222-2230 (2017).

15. Huan TN, et al. Low-cost High-efficiency System for Solar-driven Conversion of CO_2_ to Hydrocarbons. *Proc. Natl. Acad. Sci. U. S. A.* **116**, 9735-9740 (2019).

16. Ren D, Loo NWX, Gong L, Yeo BS. Continuous Production of Ethylene from Carbon Dioxide and Water Using Intermittent Sunlight. *ACS Sustain. Chem. Eng.* **5**, 9191-9199 (2017).

17. Gurudayal, et al. Si Photocathode with Ag-supported Dendritic Cu Catalyst for CO_2_ Reduction. *Energy Environ. Sci.* **12**, 1068-1077 (2019).

18. Gao J, et al. Selective C-C Coupling in Carbon Dioxide Electroreduction via Efficient Spillover of Intermediates as Supported by Operando Raman Spectroscopy. *J. Am. Chem. Soc.* **141**, 18704-18714 (2019).

19. Zhang DF, Zhang H, Guo L, Zheng K, Han XD, Zhang Z. Delicate Control of Crystallographic Facet-oriented Cu_2_O Nanocrystals and the Correlated Adsorption Ability. *J. Mater. Chem.* **19**, 5220-5225 (2009).

20. Ren D, Gao J, Zakeeruddin SM, Grätzel M. New Insights into the Interface of Electrochemical Flow Cells for Carbon Dioxide Reduction to Ethylene. *J. Phys. Chem. Lett.* **12**, 7583-7589 (2021).
